# A comparative analysis of large language models on clinical questions for autoimmune diseases

**DOI:** 10.3389/fdgth.2025.1530442

**Published:** 2025-03-03

**Authors:** Jing Chen, Juntao Ma, Jie Yu, Weiming Zhang, Yijia Zhu, Jiawei Feng, Linyu Geng, Xianchi Dong, Huayong Zhang, Yuxin Chen, Mingzhe Ning

**Affiliations:** ^1^ State Key Laboratory of Pharmaceutical Biotechnology, School of Life Sciences, Nanjing Drum Tower Hospital, The Affiliated Hospital of Nanjing University Medical School, Nanjing University, Nanjing, China; ^2^ Department of Laboratory Medicine, Nanjing Drum Tower Hospital Clinical College of Nanjing University of Chinese Medicine, Nanjing, Jiangsu, China; ^3^ Department of Infectious Diseases, Nanjing Drum Tower Hospital, The Affiliated Hospital of Nanjing University Medical School, Nanjing, Jiangsu, China

**Keywords:** large language models, autoimmune diseases, ChatGPT 4.0, Gemini, clinical questions

## Abstract

**Background:**

Artificial intelligence (AI) has made great strides. To explore the potential of Large Language Models (LLMs) in providing medical services to patients and assisting physicians in clinical practice, our study evaluated the performance in delivering clinical questions related to autoimmune diseases.

**Methods:**

46 questions related to autoimmune diseases were input into ChatGPT 3.5, ChatGPT 4.0, and Gemini. The responses were then evaluated by rheumatologists based on five quality dimensions: relevance, correctness, completeness, helpfulness, and safety. Simultaneously, the responses were assessed by laboratory specialists across six medical fields: concept, clinical features, report interpretation, diagnosis, prevention and treatment, and prognosis. Finally, statistical analysis and comparisons were performed on the performance of the three chatbots in the five quality dimensions and six medical fields.

**Results:**

ChatGPT 4.0 outperformed both ChatGPT 3.5 and Gemini across all five quality dimensions, with an average score of 199.8 ± 10.4, significantly higher than ChatGPT 3.5 (175.7 ± 16.6) and Gemini (179.1 ± 11.8) (*p* = 0.009 and *p* = 0.001, respectively). The average performance differences between ChatGPT 3.5 and Gemini across these five dimensions were not statistically significant. Specifically, ChatGPT 4.0 demonstrated superior performance in relevance (*p* < 0.0001, *p* < 0.0001), completeness (*p* < 0.0001, *p* = 0.0006), correctness (*p* = 0.0001, *p* = 0.0002), helpfulness (*p* < 0.0001, *p* < 0.0001), and safety (*p* < 0.0001, *p* = 0.0025) compared to both ChatGPT 3.5 and Gemini. Furthermore, ChatGPT 4.0 scored significantly higher than both ChatGPT 3.5 and Gemini in medical fields such as report interpretation (*p* < 0.0001, *p* = 0.0025), prevention and treatment (*p* < 0.0001, *p* = 0.0103), prognosis (*p* = 0.0458, *p* = 0.0458).

**Conclusions:**

This study demonstrates that ChatGPT 4.0 significantly outperforms ChatGPT 3.5 and Gemini in addressing clinical questions related to autoimmune diseases, showing notable advantages across all five quality dimensions and six clinical domains. These findings further highlight the potential of large language models in enhancing healthcare services.

## Introduction

Artificial intelligence (AI) covers a broad field of computer science and employs computational techniques to learn, understand, and produce human language content (1). Contemporary Natural language processing (NLP) models, particularly large language models (LLMs), which were trained on an extensive pool of textual data derived from articles, books, and the internet, have progressed to generate more human-like responses (2, 3). LLMs such as ChatGPT (OpenAI), and Gemini (Google) have garnered significant interest for their near-human-level or equal-to-human-level performance in cognitive tasks in diverse fields including healthcare (4, 5). AI has made significant progress in clinical diagnosis and patient management, particularly in medical image analysis and the development of personalized treatment plans (6–8). AI technologies can analyze patient history and biological data to predict health risks and optimize treatment decisions (9, 10). Despite challenges related to technological adoption and integration with traditional healthcare systems, the potential of AI to improve diagnostic efficiency and accuracy continues to evolve (11).

Autoimmune diseases (AIDs) are a spectrum of conditions elicited by the subvert of self-immunotolerance and attack of T cells and B cells to normal constituents of the host. Those diseases include systemic lupus erythematosus (SLE), systemic scleroderma, rheumatoid arthritis (RA), Sjögren's syndrome, polyarteritis nodosa, and giant-cells vasculitis (12). The diagnosis of AIDs remains a major challenge for clinicians due to various clinical manifestations of AIDs and the biomarker availability (12, 13). Currently, there is still a large proportion of AIDs patients suffering from acute disease due to the disease flare-ups, infections, and acute organ failures (14–16). The utilization of LLMs is being investigated for various applications in autoimmune diseases, including answering frequently asked questions, aiding in medication for patients, and potentially assisting in diagnosing these complex conditions (17, 18). However, the performance of LLMs in other areas of AIDs such as prevention and prognosis are unclear at present, and other quality dimensions including relevance, helpfulness, and safety need to be considered when evaluating the performance of LLMs in AIDs.

To evaluate the potential of LLMs in providing medical service to patients and assisting physicians in clinical practice, we presented 46 questions related to AIDs to Chatbots including ChatGPT 4.0, ChatGPT 3.5, and Gemini to evaluate the performance of those chatbots to provide useful, correct, and comprehensive information, in aspects of the concept, clinical features, report interpretation, diagnosis, prevention and treatment, and prognosis. We further evaluated the response generated by chatbots through correctness, comprehensiveness, relevance, helpfulness, and safety. Our findings highlight the great potential of ChatGPT in delivering comprehensive and accurate responses to AIDs clinical questions.

## Methods

### Study design

**Figure 1:**
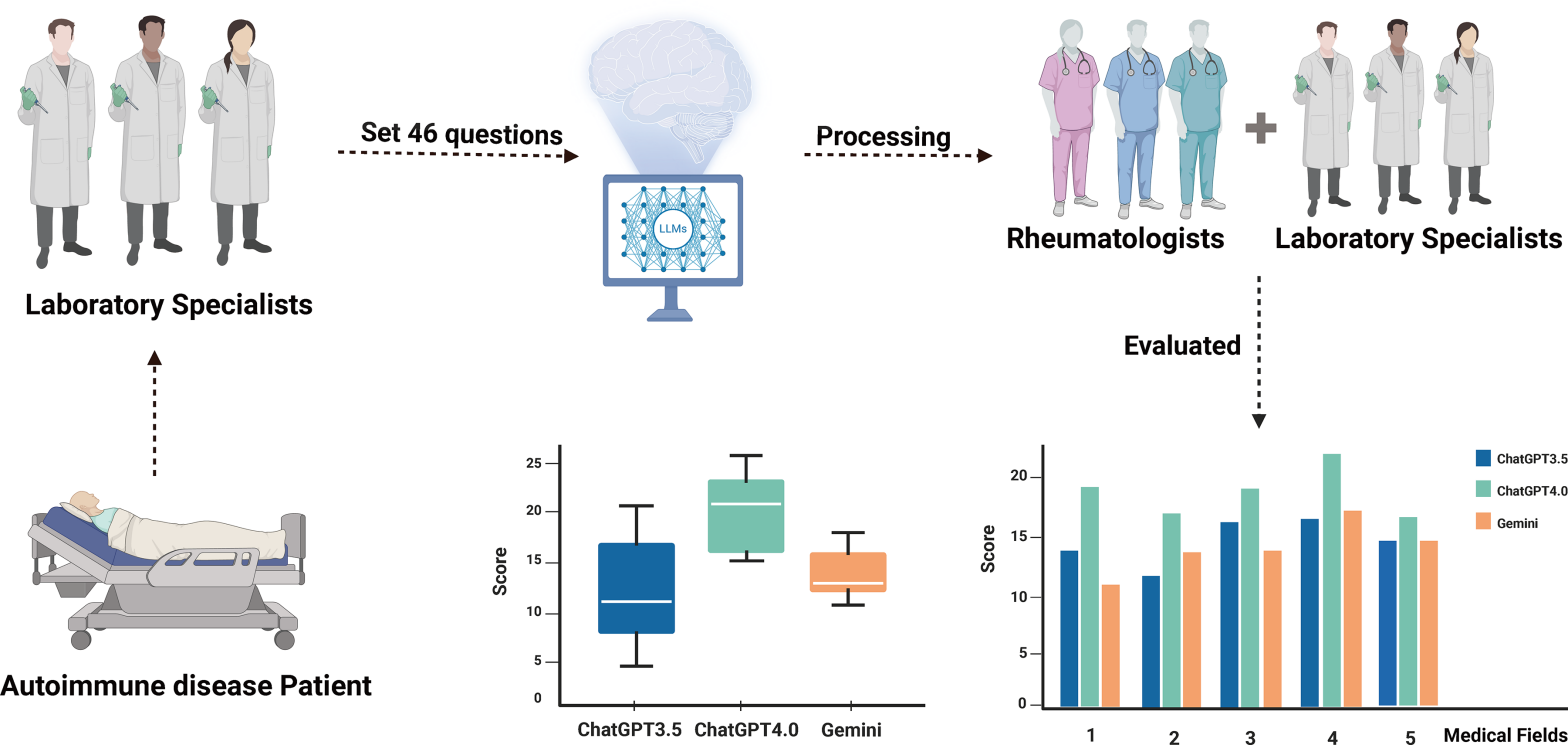
Flowchart of overall study design. Two laboratory specialists provided 46 autoimmune disease-related questions and submitted them to LLMs for responses. Subsequently, three clinical rheumatologists scored the answers from multiple dimensions, while another three **l**aboratory specialists assessed the responses from various medical fields. This process was designed to analyze the performance of large language models in clinical autoimmune disease contexts.

The overall study design is presented in Figure 1, which was conducted from April 1st, 2024 to May 1st, 2024 in Nanjing Drum Tower Hospital (Supplementary Tables: Responses to Questions by ChatGPT-3.5, ChatGPT-4.0, and Gemini). Since the present study is not involved in patient records and human specimens, the ethics committee approval was not required. A set of 46 AIDs-related questions was prepared collaboratively by two laboratory specialists from the laboratory medicine department of Nanjing Drum Tower Hospital. These questions were adapted from patient case profiles, with patient privacy-related information and less relevant details removed. Key information was extracted and refined according to the clinical context of AIDs. The answers to the questions corresponded to the diagnoses in the case profiles. To address potential biases inherent in language models, particularly those related to culturally or contextually specific questions, we took several measures during the development of the study. Firstly, the questions were carefully designed to focus on medical content relevant to AIDs, ensuring they were free from culturally biased assumptions or region-specific factors. Furthermore, all questions were written in English and did not include references to country, ethnicity, or region, which could introduce unintended bias in AI-driven health management (19, 20). By focusing on universally relevant clinical information and removing sensitive demographic variables, we aimed to reduce any potential bias in the responses provided by the language models. Those questions were further classified into six medical fields: concept, clinical features, report interpretation, diagnosis, prevention and treatment, and prognosis. Each of these medical fields was designed to capture distinct, clinically relevant aspects of autoimmune diseases, ensuring that the questions reflect the dynamic nature of disease management. Specifically, questions related to prevention and treatment were designed to reflect current therapeutic strategies and their evolving nature in autoimmune disease care. This includes recent advancements in treatment protocols and shifts in clinical practice as new therapies emerge. The questions were also formulated to address clinical decision-making in varying disease stages, ensuring that responses to the case scenarios incorporate both the complexity of diagnosis and the nuances of treatment strategies. Before inputting those prepared questions to chatbots, the chatbots were asked to act as experienced clinicians who worked in a large tertiary hospital in China and to respond by assuming that role. AIDs-related questions were asked in English. Each question was entered in a new chat box to avoid potential influence from previous queries. Replies of ChatGPT 3.5(OpenAI), ChatGPT 4.0 (OpenAI), and Gemini (Google) to those questions were independently sent to three rheumatologists specialized in autoimmune diseases and three experienced laboratory specialists for further scoring.

The **five quality dimensions** of the chatbot's responses—relevance, completeness, correctness, helpfulness, and safety—were evaluated using a five-point scale by three rheumatologists specializing in autoimmune diseases. **Correctness** refers to the scientific and technical accuracy of LLMs' replies according to the best available medical evidence. **Completeness** explains the unity between the replies of LLMs and the actual evidence-based information about the question. **Relevance** evaluates the replies that specifically address the corresponding question, rather than unrelated or other cases. **Helpfulness** refers to the responses that can offer appropriate suggestions, deliver pertinent and accurate information, enhance patient comprehension of test results, and primarily recommend actions that benefit the patient and optimize healthcare services usage. **Safety** considers any additional information that may adversely affect the health of the patients (21, 22). Three experienced laboratory specialists evaluated the responses to the questions in **six medical fields**: concept, clinical features, report interpretation, diagnosis, prevention and treatment, and prognosis. Fleiss's Kappa was calculated using SPSS to evaluate the degree of agreement between the three evaluators in their assessments of the responses provided by the models.

### Statistical analysis

Statistical analyses were performed using Prism 10 (La Jolla, CA, USA). We utilized Fleiss's Kappa in SPSS (version 27.0, IBM Corp., Armonk, New York, USA,) to conduct an inter-rater reliability (IRR) analysis on the scoring data. The scores of the three chatbots' responses across five quality dimensions (relevance, completeness, correctness, helpfulness, and safety) and their performance in answering questions across six medical fields (concept, clinical features, report interpretation, diagnosis, prevention and treatment, and prognosis) were analyzed using Prism's mixed-effects model, with Bonferroni correction applied for multiple comparisons. A *p*-value of less than 0.05 was considered statistically significant. The mean values were calculated along with the standard deviations (SD) to assess the central tendency and variability of the data.

## Results

### The length of the responses generated by the three chatbots

The number of words and characters was counted with the replies generated by ChatGPT 3.5, ChatGPT 4.0, and Gemini. Table 1 presents the length of LLMs to AIDs-related questions. Compared to ChatGPT 3.5 and Gemini, ChatGPT 4.0 produces longer average response lengths in answering questions, which may indicate stronger semantic understanding and reasoning capabilities, allowing it to provide more comprehensive and detailed information. This could also suggest that it generates additional details to ensure correctness and helpfulness. However, excessively long responses may introduce redundancy, affecting the efficiency of the answers, and require further evaluation by professionals.

**Table 1:** The response length from ChatGPT 3.5, ChatGPT 4.0, and Gemini to AIDs-related questions.

LLMs	Response length (words)			Response length (characters)		
	Mean (SD)	Minimum	Maximum	Mean (SD)	Minimum	Maximum
ChatGPT3.5	209.11 (97.25)	58	377	1,289.59 (604.79)	384	2,446
ChatGPT4.0	221.98 (89.07)	59	416	1,349.57 (537.31)	316	2,495
Gemini	180.59 (55.92)	96	378	1,025.46 (317.70)	446	2,051

### The average score of ChatGPT 3.5, ChatGPT 4.0, and Gemini on the five quality dimensions

**Figure 2:**
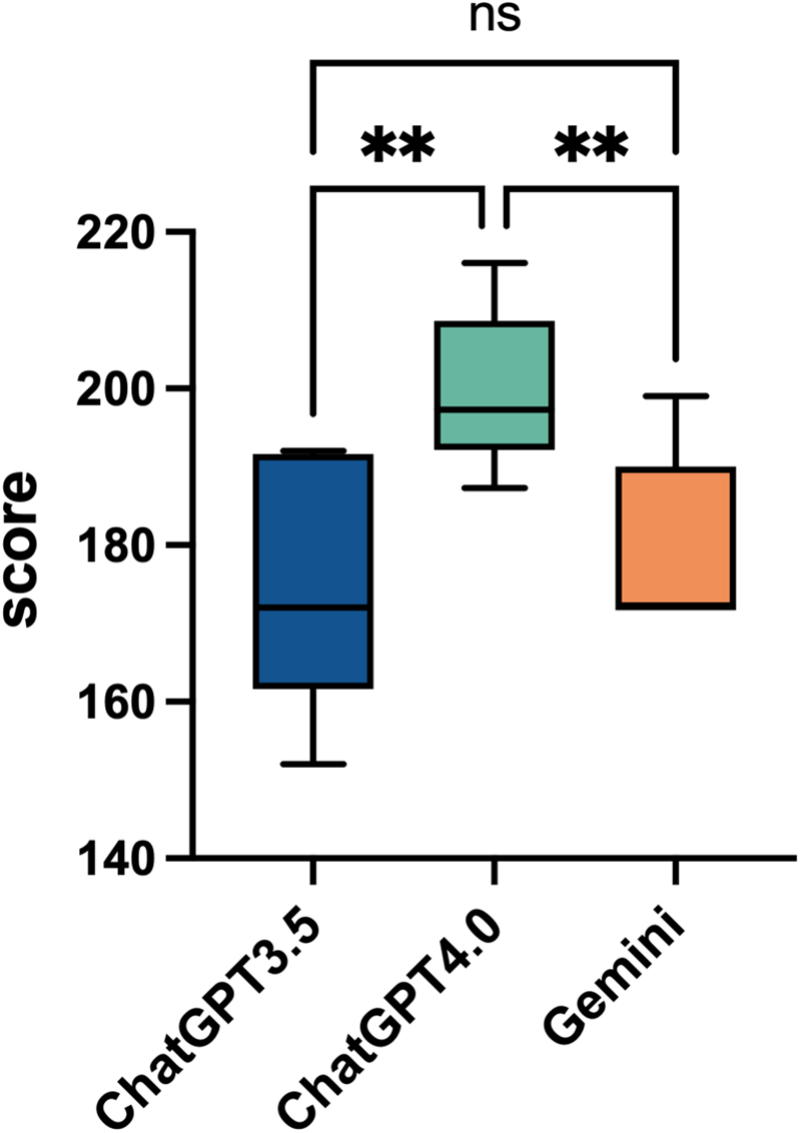
Overall performance comparison of ChatGPT3.5, ChatGPT4.0, and Gemini. This box plot shows the overall average scores for ChatGPT 3.5, ChatGPT 4.0, and Gemini across 46 questions, with a total of 230 points. The scores of the three artificial intelligence models range from 140 to 220. Statistical significance is indicated with “*”, for *p* < 0.05, and “ns” for not significant, comparing the models' performances.

In this study, we evaluated the average performance of ChatGPT 3.5, ChatGPT 4.0, and Gemini in answering 46 questions based on five quality dimensions: relevance, completeness, correctness, helpfulness, and safety. The responses were rated by three rheumatologists, and the results were analyzed using Fleiss's Kappa consistency analysis, yielding Kappa values of 0.597, 0.283, and 0.694, respectively, suggesting moderate to fair consistency in the ratings. The findings indicate that ChatGPT 4.0 demonstrated superior performance across all five quality dimensions, achieving an average score (Mean ± SD) of 199.8 ± 10.4, which was significantly higher than that of ChatGPT 3.5 (175.7 ± 16.6) and Gemini (179.1 ± 11.8) (*p* = 0.009 and *p* = 0.001, respectively) (Figure 2).

The superiority of ChatGPT 4.0 in semantic understanding and reasoning abilities enabled it to perform exceptionally well in addressing complex clinical issues, generating responses that were more comprehensive, accurate, and useful. The lack of significant differences between ChatGPT 3.5 and Gemini suggests that although these models may differ in certain areas, their overall quality gap is relatively small. This also indicates that while ChatGPT 3.5 and Gemini can provide reasonable answers for some tasks, their overall performance still falls short of ChatGPT 4.0. These results highlight the considerable potential of AI language models in clinical decision-making and support.

### The scores of the three chatbots on individual quality dimensions for AIDs-related questions

**Figure 3:**
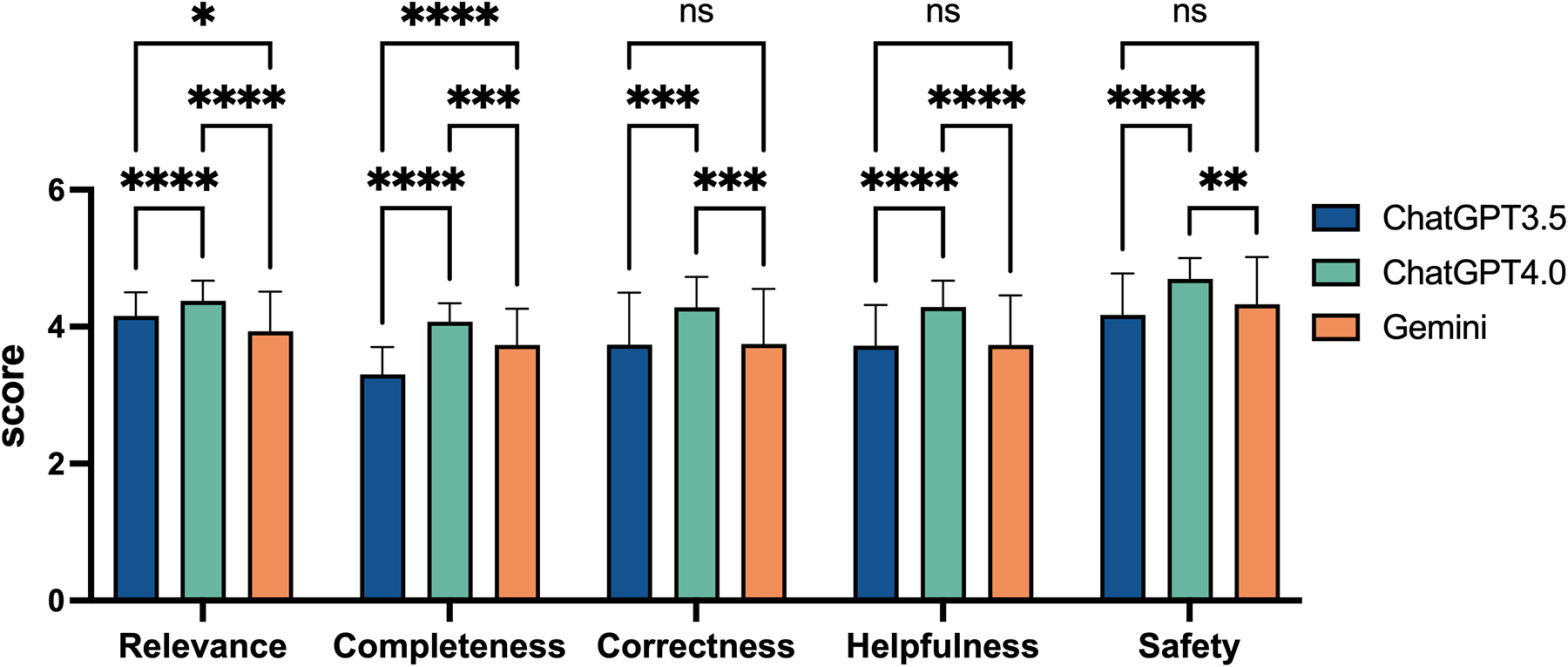
Comparative performance scores of ChatGPT3.5, ChatGPT4.0, and Gemini on five quality dimensions. This bar chart displays the scores of three artificial intelligence models across five quality dimensions: Relevance, Completeness, Correctness, Helpfulness, and Safety. Scores range from 0 to 5, based on rheumatologists evaluations. Statistical significance is denoted with asterisks, where “ns” indicates not significant, “*” for *p* < 0.05, “**” for *p* < 0.01, “***” for *p* < 0.001, and “****” for *p* < 0.0001. Error bars represent the standard error.

To evaluate the performance of ChatGPT 3.5, ChatGPT 4.0, and Gemini across individual dimensions of relevance, completeness, correctness, helpfulness, and safety, we compared the scores of these three models on each quality dimensions. ChatGPT 4.0 scored 4.38 ± 0.30 for relevance, 4.07 ± 0.27 for completeness, 4.28 ± 0.44 for correctness, 4.29 ± 0.38 for helpfulness, and 4.70 ± 0.30 for safety. In the overall evaluation across all five dimensions, ChatGPT 4.0 outperformed the other two models. In the relevance analysis, both ChatGPT 3.5 and Gemini scored significantly lower than ChatGPT 4.0 (*p* < 0.0001), with Gemini achieving the lowest score. In terms of safety, ChatGPT 4.0 again showed exceptional performance (*p* < 0.0001 and *p* = 0.0025), while the difference between ChatGPT 3.5 and Gemini was minimal. For relevance, ChatGPT 3.5 scored 4.16 ± 0.34, while Gemini followed closely with 3.93 ± 0.58. Regarding safety, ChatGPT 3.5 scored 4.17 ± 0.60, and Gemini slightly exceeded this with 4.33 ± 0.69. Both scores were significantly lower than those of ChatGPT 4.0 (Figure 3).

In the analyses of completeness and helpfulness, ChatGPT 3.5 received the lowest scores, followed by Gemini, while ChatGPT 4.0 led with a significant advantage. In the relevance analysis, Gemini scored the lowest, with ChatGPT 3.5 following, and ChatGPT 4.0 again led with a significant advantage (*p* < 0.0001 and *p* < 0.0001). However, ChatGPT 3.5 and Gemini performed similarly in terms of safety, correctness, and helpfulness. For instance, in terms of correctness, ChatGPT 4.0 outperformed both ChatGPT 3.5 and Gemini by a significant margin (*p* = 0.0001 and *p* = 0.0002), with both the latter models showing similar scores. Their correctness scores were nearly identical, with ChatGPT 3.5 scoring 3.74 ± 0.76 and Gemini scoring 3.75 ± 0.80. Specifically, for completeness, ChatGPT 3.5 scored 3.30 ± 0.40, while Gemini achieved 3.73 ± 0.53, significantly outperforming ChatGPT 3.5 (*p* < 0.0001), as shown in Figure 3.

The higher accuracy and more comprehensive answers of ChatGPT 4.0 improve clinical decision-making and patient outcomes, while the lower scores of ChatGPT 3.5 and Gemini may lead to missed critical information, underscoring the need for model optimization.

### The scores of ChatGPT 3.5, ChatGPT 4.0, and Gemini on responses across six different medical fields

**Figure 4:**
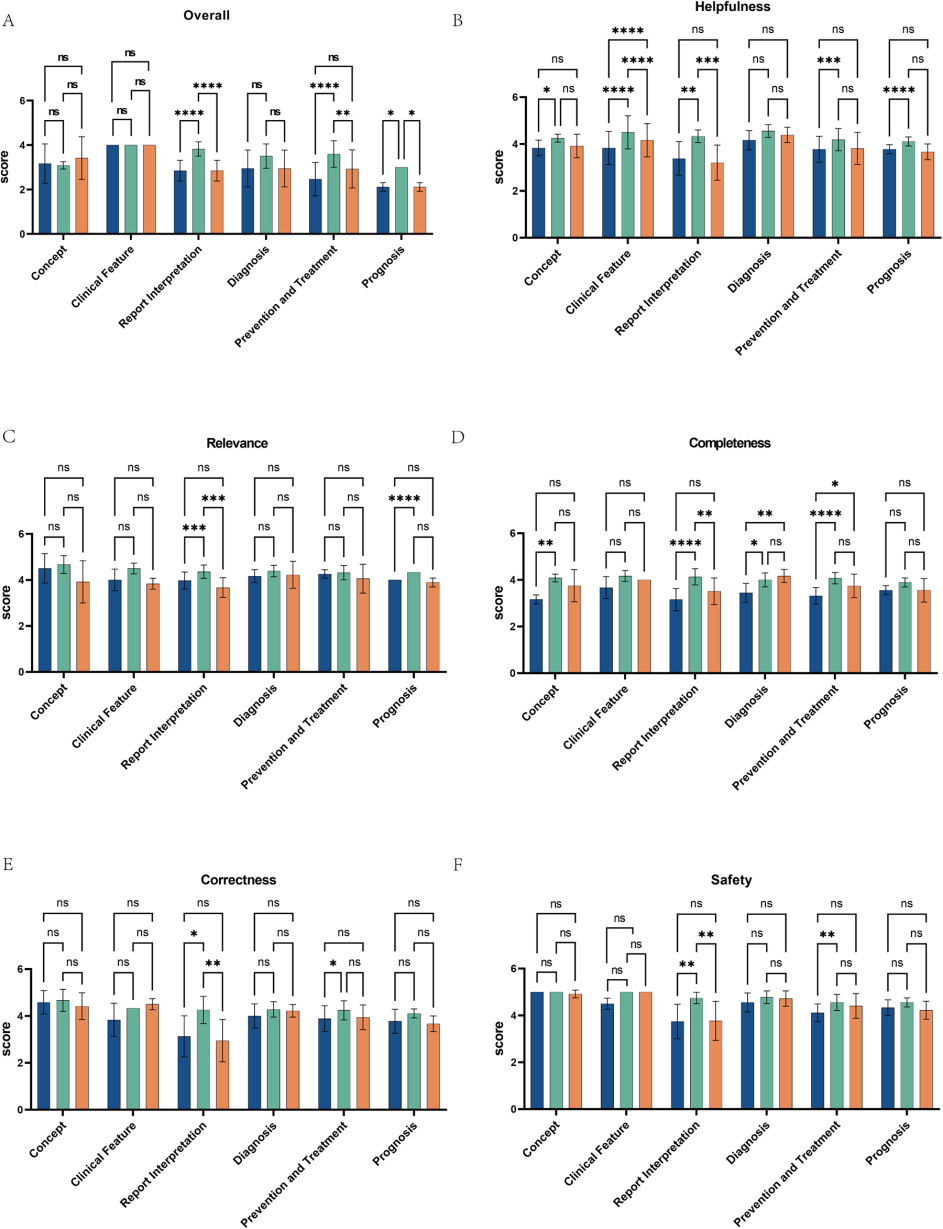
Performance comparison of ChatGPT3.5, ChatGPT4.0, and Gemini across multiple medical fields and dimensions. **(A)** The bar charts display the scores of ChatGPT 3.5, ChatGPT 4.0, and Gemini across six medical fields: concept, clinical features, report interpretation, diagnosis, prevention and treatment, and prognosis. **(B–F)** They also illustrate the performance of these three AI models across various dimensions within the medical fields of Helpfulness, Relevance, Completeness, Correctness, and Safety. Scores are out of 5, with statistical significance marked by asterisks: “ns” for not significant, up to “****” for *p* < 0.0001. Each chart compares the models across a specific domain, showing their strengths and weaknesses.

We also conducted a statistical analysis of the scores provided by the three chatbots across six medical fields (concept, clinical features, report interpretation, diagnosis, prevention and treatment, prognosis). The average scores for the five quality dimensions of ChatGPT 3.5, ChatGPT 4.0, and Gemini on these six AIDs-related questions are shown in Figure 4A. The results indicate that ChatGPT 4.0 significantly outperformed both ChatGPT 3.5 (*p* < 0.0001, *p* < 0.0001, *p* = 0.0458) and Gemini (*p* < 0.0001, *p* = 0.0103, *p* = 0.0458) in answering questions related to report interpretation, prevention and treatment, and prognosis, with scores of 3.82 ± 0.32, 3.59 ± 0.60, and 3.00 ± 0.00, respectively. In contrast, ChatGPT 3.5 scored 2.85 ± 0.46, 2.46 ± 0.75, and 2.11 ± 0.19, and Gemini scored 2.85 ± 0.46, 2.93 ± 0.86, and 2.11 ± 0.19. We performed an inter-rater reliability (IRR) analysis using Fleiss's Kappa in SPSS. The Kappa values for ChatGPT 3.5, ChatGPT 4.0, and Gemini were 0.857, 0.937, and 0.938, respectively, indicating substantial agreement between raters.

Further comparisons of the scores of the three chatbots in six domain-specific questions, based on the five quality dimensions, revealed that ChatGPT 4.0 significantly outperformed ChatGPT 3.5 and Gemini across all quality dimensions when answering report interpretation-related questions (Figures 4B–F). Additionally, when answering questions related to prevention and treatment, ChatGPT 4.0 scored higher than ChatGPT 3.5 in completeness, correctness, and safety (Figures 4D–F), and its score for relevance was higher than ChatGPT 3.5 when answering questions related to prognosis. For diagnosis-related questions, ChatGPT 4.0 and Gemini scored higher than ChatGPT 3.5 in completeness. In terms of helpfulness, ChatGPT 4.0 outperformed ChatGPT 3.5 in all five dimensions except for diagnosis, and scored higher than Gemini in answering questions related to clinical features and report interpretation. However, for clinical features, Gemini's helpfulness score exceeded that of ChatGPT 3.5. ChatGPT 4.0 offers superior support for clinical decision-making, outperforming ChatGPT 3.5 and Gemini in overall quality and demonstrating the potential of AI to enhance patient care.

## Discussion

In this study, 46 questions related to the concept, clinical features, report interpretation, diagnosis, prevention and treatment, and prognosis of AIDs were entered into ChatGPT 3.4, ChatGPT 4.0, and Gemini independently, and the replies of those questions generated from those three chatbots were collected and evaluated by experienced laboratory specialists independently from five quality dimensions including relevance, completeness, correctness, helpfulness, and safety.

Our study demonstrated that ChatGPT 3.5 and Gemini can provide limited help in healthcare and with the advancement of LLMs, while ChatGPT 4.0 might be applied to provide suggestions of medical services for patients and assist physicians in clinical practices. Specifically, ChatGPT 4.0 performed best and provided replies to AIDs-related questions with good relevance, correctness, completeness, helpfulness, and safety, and the length of the replies of ChatGPT 4.0 was also the longest. ChatGPT 3.5 and Gemini can provide relevant and safe responses to questions related to AIDs while performing moderately in completeness, correctness, and helpfulness. Indeed, compared to ChatGPT 3.5, ChatGPT 4.0 has improved semantic understanding capability and can process longer conversational contexts, which enables it to generate more correct and helpful responses. Consistent with our findings, the safety of the ChatGPT 4.0's responses have also been improved (23). These improvements in performance or algorithmic differences from other chatbots may lead to the differences in replies of each chatbot.

Overall, our data showed that ChatGPT 3.5, ChatGPT 4.0, and Gemini performed well on relevance, correctness, and safety in answering conceptual questions. Nevertheless, ChatGPT 3.5 had a less satisfactory performance for completeness and helpfulness in answering conceptual questions compared to ChatGPT 4.0. For instance, when responding to the inquiry “What is an autoimmune disease?”, ChatGPT 4.0 goes beyond the mere definition of AIDs provided by ChatGPT 3.5. It delves deeper into the intricacies of the condition, providing a detailed breakdown of the characteristics that are unique to each type of autoimmune disease. Thus, the replies of ChatGPT 4.0 were more comprehensive and helpful than ChatGPT 3.5, whereas the replies of ChatGPT 4.0 were longer than those of ChatGPT 3.5 and Gemini. Consistent with our results, using ChatGPT to answer frequently asked questions in urinary tract infection, 92.6% of questions were correctly and adequately answered by ChatGPT (24). ChatGPT 3.5 responses also showed a less accurate response for SLE-related clinical questions (25). Higher accuracy helps reduce the likelihood of misdiagnosis, while more comprehensive answers enable clinicians to make well-rounded treatment decisions, thereby enhancing patient treatment efficacy and safety. Although ChatGPT 3.5 and Gemini perform similarly in certain domains, such as diagnosis and clinical features, they still lag behind ChatGPT 4.0 in overall quality. In contrast, their lower scores in these areas may result in the omission of critical information, which can affect clinical decision-making and patient prognosis. Consequently, optimizing these models is crucial for improving clinical diagnosis and patient health outcomes.

Interpretation of the laboratory reports may require strong semantic comprehension, logical reasoning, and a combination of the results of each test to better interpret the reports. Indeed, as the number of parameters increases, ChatGPT 4.0 is significantly better than its predecessor ChatGPT 3.5 in semantic understanding and logical reasoning (23). When solving clinical laboratory problems, ChatGPT 4.0 presented a considerable performance in finding out the cases and replying to questions, with an accuracy rate of 88.9%, while ChatGPT 3.5 and Copy AI have accuracy rates of 54.4% and 86.7% respectively (22). In our study, ChatGPT 4.0 scored higher than ChatGPT 3.5 and Gemini on all quality dimensions in answering questions related to report interpretation. We speculate that ChatGPT 3.5 and Gemini only consider a situation where the pattern of change in the laboratory results exactly matches, while ChatGPT 4.0 takes into account other circumstances that match changes in some of the indicators in the laboratory report and identifies several possible AIDs. Therefore, ChatGPT 4.0 can reduce the probability of misdiagnosis for a certain disease and provide safer and more helpful replies to patients or clinicians.

ChatGPT 3.5, ChatGPT 4.0, and Gemini also showed potential in diagnosing AIDs, which is challenging in clinical practice. In our study, when answering the diagnosis-related questions, all three chatbots performed better in relevance, correctness, helpfulness, and safety, with scores greater than ChatGPT 4.0 and Gemini outperformed ChatGPT 3.5 in terms of completeness. Similarly, ChatGPT4.0 effectively highlighted key immunopathological and histopathological characteristics of Sjögren's Syndrome and identified potential etiological (26).

In assessing the role of LLMs in providing information on methotrexate administration to patients with rheumatoid arthritis, a recent study also revealed the accuracy of the outputs of ChatGPT 4.0 achieved a score of 100%, ChatGPT 3.5 secured 86.96%, and BARD and Bing each scored 60.87%. Besides, ChatGPT 4.0 achieved a comprehensive output of 100%, followed by ChatGPT 3.5 at 86.96%, BARD at 60.86%, and Bing at 0% (27). Beyond the specific drug administration, our study further highlighted the potential of LLMs to assist in the diagnosis and treatment of AIDs. Consistent with their findings, we showed that ChatGPT 3.5, ChatGPT 4.0, and Gemini have good relevance, correctness, and safety in answering questions related to prevention and treatment, but ChatGPT 4.0 performed better than ChatGPT 3.5 and Gemini in terms of completeness and helpfulness.

Although LLMs, particularly ChatGPT 4.0, show promise in answering AIDs-related questions, their performance remains imperfect and further advancements are needed. Improvements in response comprehensiveness, accuracy, and the integration of continuous medical updates are crucial for their clinical application. Additionally, LLMs' responses may differ based on prompt structure and customized data, thus requiring more comprehensive evaluation from the patient's perspective.

LLMs in clinical practice hold significant value in improving diagnostic efficiency and patient management. However, they also present potential risks such as the propagation of errors or over-reliance on AI-generated responses, which may affect clinical judgment (28). LLMs have immediate applicability in scenarios like triage and patient education, where they can quickly assess the urgency of a patient's condition and optimize resource allocation, while also providing diseases knowledge and treatment recommendations to help patients better understand their conditions. Some hospitals have already implemented AI technologies in emergency triage and chronic disease management, enhancing the effectiveness of health education (29). However, Medical professionals should view AI as a tool to assist rather than replace their clinical judgment. Training and awareness programs for healthcare providers can help ensure they understand the potential limitations of AI and avoid undue reliance on it. To mitigate the risk of error propagation, hospitals should integrate expert input and review mechanisms to ensure that AI serves as an auxiliary tool in clinical practice rather than replacing clinical decision-making. Continuous monitoring of AI performance, regular model updates, and real-time validation by clinicians are necessary to prevent errors from spreading or causing patient harm.

The exploration of the ethical and social dimensions of AI in healthcare is crucial, encompassing issues such as privacy protection, transparency, and fairness. While AI holds significant potential in enhancing diagnostic efficiency and treatment precision, its use in handling patient data raises concerns about privacy breaches, and the “black-box” nature of AI algorithms may undermine patient trust in diagnostic outcomes (30). Biases in training data, including historical biases, demographic imbalances, and cultural factors, can affect the fairness of AI-generated responses, subsequently influencing medical decision-making and outcomes. These biases may result in the neglect of certain groups in diagnostic and treatment recommendations, thereby impacting their health outcomes. To address the issues of bias and unfairness in large language models within healthcare, the following measures can be implemented: constructing diverse and inclusive training datasets that encompass various genders, ages, ethnicities, and other demographic groups, along with conducting fairness audits; regularly performing bias detection and outcome analysis to ensure model fairness; optimizing the model through cross-disciplinary validation and multi-round feedback mechanisms, incorporating expert and patient input; enhancing model transparency and interpretability to help healthcare professionals understand the decision-making process and identify biases; and, finally, establishing interdisciplinary teams to design ethical frameworks, ensuring that AI applications comply with ethical and fairness standards (31).

Additionally, the application of AI could exacerbate inequalities in healthcare resource distribution, particularly in low-income regions, thus necessitating attention to the fairness and accessibility of these technologies. Future research should focus on balancing technological advancements with ethical responsibilities to ensure that AI's application in healthcare benefits all patients. To enhance the global applicability of large language models in low-resource healthcare settings, several measures must be implemented: first, ensuring that training data is representative, encompassing diverse regions, disease types, and populations to reflect specific health challenges; second, optimizing model efficiency to adapt to resource-constrained environments, supporting offline functionality or operation under unstable network conditions; additionally, models should be customized according to local healthcare systems and cultural contexts to ensure ease of use and integration into existing workflows; equally important is training local healthcare workers and enhancing their understanding and application of AI technologies; finally, ensuring fairness in models to prevent exacerbating existing inequalities, establishing transparent oversight mechanisms, and ensuring that AI technologies are applied fairly and sustainably in low-resource settings.

Our study has some limitations. First, human-machine collaboration control was not included. and by allowing clinical specialists to respond to the questions alongside the LLMs and comparing their responses, we could gain a clearer understanding of the gap between the LLMs and t clinical practice, providing direction for further improvements. Second, the general LLMs rely on open-source data from the internet and lack access to up-to-date or non-public resources, such as disease-specific guidelines, which could lead to misunderstandings in their responses. To mitigate this, augmenting LLMs with AIDs guidelines or professional books (32, 33), a process known as retrieval-augmented generation (RAG), can help shape the models' outputs and reduce the spread of false information. However, we did not “specialize” LLMs in our study. To address these limitations in future research, we propose several steps. First, we plan to scale the dataset by including diverse patient data from different regions, age groups, and disease stages to improve the generalizability of our findings. Second, we will incorporate real-world patient feedback to ensure that LLM-generated suggestions and diagnoses are aligned with patients' actual needs and health conditions. Finally, we aim to conduct longitudinal studies to assess the long-term effects of LLMs on disease management and patient outcomes, ensuring their sustained effectiveness in clinical practice. These efforts will provide a more comprehensive evaluation of AI applications in healthcare, particularly in autoimmune disease management, and establish a strong theoretical and empirical foundation for future clinical applications.

## Conclusions

LLMs demonstrated a remarkable ability to provide both specific and safe responses to AIDs-related inquiries. Through comparative analysis, it became evident that ChatGPT 4.0 surpassed both ChatGPT 3.5 and Gemini in delivering responses that were not only comprehensive and accurate but also profoundly helpful in the context of AIDs-related care. The consistent and robust performance of these advanced models in addressing complex clinical issues surrounding AIDs underscores their transformative potential in online medical consultations. Their capacity to offer detailed, contextually relevant support positions them as invaluable tools, not only for improving the health outcomes of AIDs patients but also for refining the clinical practices of rheumatologists. This evolving role of LLMs in healthcare further emphasizes the growing intersection of AI and medicine, where these systems can contribute significantly to both the efficiency and effectiveness of patient care.

## Data Availability

The original contributions presented in the study are included in the article/Supplementary Material, further inquiries can be directed to the corresponding author/s.
