# Supplementary material for: A comparative analysis of large language models on clinical questions for autoimmune diseases
Source: Front Digit Health. 2025 Mar 3;7:1530442. doi: 10.3389/fdgth.2025.1530442 (PMC11913117; doi:10.3389/fdgth.2025.1530442)
Supplement: Supplementary file 1 [file Table1.docx]

# Supplementary Table 1a: ChatGPT-3.5 Responses to Questions

| **Question** | **ChatGPT-3.5 Response** |
| --- | --- |
| **Topic: Concept** | |
| 1. What is an autoimmune disease? | An autoimmune disease is a condition where the immune system mistakenly attacks the body's own cells and tissues. Normally, the immune system is tasked with defending the body against harmful invaders like bacteria and viruses. However, in autoimmune diseases, the immune system malfunctions and starts targeting healthy cells instead. This can lead to inflammation, tissue damage, and various symptoms depending on the specific autoimmune condition.  There are over 80 known autoimmune diseases, including rheumatoid arthritis, lupus, type 1 diabetes, multiple sclerosis, and inflammatory bowel disease, among others. These diseases can affect virtually any part of the body, and their exact causes are not fully understood. Factors such as genetics, environmental triggers, and hormonal influences may contribute to the development of autoimmune diseases.  Diagnosis of autoimmune diseases often involves a combination of medical history, physical examination, laboratory tests to detect autoantibodies or inflammation markers, and sometimes imaging studies to assess organ damage. Treatment typically focuses on managing symptoms, controlling inflammation, and suppressing the immune response to prevent further damage to tissues. This may involve medications such as corticosteroids, immunosuppressants, or biologic therapies, as well as lifestyle changes and supportive therapies. |
| 1. What diseases are included in autoimmune diseases? | Autoimmune diseases encompass a broad range of conditions where the immune system mistakenly attacks the body's own tissues. Some common autoimmune diseases include:  1. Rheumatoid arthritis  2. Systemic lupus erythematosus (SLE)  3. Multiple sclerosis (MS)  4. Type 1 diabetes  5. Hashimoto's thyroiditis  6. Graves' disease  7. Inflammatory bowel disease (Crohn's disease and ulcerative colitis)  8. Psoriasis  9. Celiac disease  10. Addison's disease  These are just a few examples, as there are over 80 known autoimmune diseases, each affecting different parts of the body. It's important to note that autoimmune diseases can vary widely in severity and symptoms, and often require specialized medical care for proper management. |
| 1. What are autoantibodies? | Autoantibodies are antibodies produced by the immune system that mistakenly target and attack the body's own tissues and cells. Normally, the immune system produces antibodies to identify and neutralize foreign substances such as bacteria and viruses. However, in autoimmune diseases, the immune system malfunctions and produces autoantibodies that target normal proteins, cells, and tissues of the body.  These autoantibodies can cause inflammation, tissue damage, and dysfunction in various organs and systems, depending on the specific autoimmune disease. For example, in rheumatoid arthritis, autoantibodies target the joints, leading to joint pain and inflammation. In lupus, autoantibodies attack multiple organs and tissues, resulting in a range of symptoms such as joint pain, skin rashes, and fatigue.  Autoantibodies can be detected through laboratory tests, such as blood tests, which help in the diagnosis and management of autoimmune diseases. |
| 1. What are antinuclear antibodies? | Antinuclear antibodies (ANAs) are autoantibodies that target components of the cell nucleus. These antibodies can be detected in the blood of individuals with autoimmune diseases such as systemic lupus erythematosus (SLE), Sjögren's syndrome, rheumatoid arthritis, and other connective tissue diseases.  The presence of ANAs in the blood is often indicative of an autoimmune disorder, but it's important to note that not all individuals with ANAs develop autoimmune diseases, and the presence of ANAs alone does not confirm a specific diagnosis. ANA testing is typically performed using techniques such as indirect immunofluorescence or enzyme-linked immunosorbent assay (ELISA) to detect the presence of these antibodies in a patient's blood sample.  Interpreting ANA test results requires consideration of various factors, including the patient's clinical symptoms, medical history, and other laboratory tests. A positive ANA test result may prompt further evaluation and monitoring to help diagnose and manage autoimmune conditions. |
| **Topic: Clinical Feature** | |
| 1. What clinical symptoms should be considered to exclude autoimmune diseases? | When considering autoimmune diseases, there's a broad range of symptoms and manifestations to keep in mind. Here are some key clinical symptoms that might warrant investigation to exclude autoimmune diseases:  1. **Joint Pain and Swelling**: Conditions like rheumatoid arthritis can present with joint pain, stiffness, and swelling.  2. **Fatigue**: Chronic fatigue is a common symptom in autoimmune diseases such as lupus and multiple sclerosis.  3. **Skin Rashes**: Conditions like lupus often present with characteristic skin rashes, such as a butterfly rash on the face.  4. **Muscle Weakness**: Autoimmune diseases like myasthenia gravis can lead to muscle weakness and fatigue, particularly in the facial muscles.  5. **Fever**: Unexplained fevers can sometimes be a sign of autoimmune activity, especially in systemic autoimmune diseases like lupus.  6. **Digestive Issues**: Conditions like Crohn's disease and ulcerative colitis are autoimmune diseases that affect the digestive system, leading to symptoms such as abdominal pain, diarrhea, and blood in the stool.  7. **Neurological Symptoms**: Autoimmune diseases such as multiple sclerosis and Guillain-Barré syndrome can cause a variety of neurological symptoms, including numbness, tingling, weakness, and difficulty with coordination or balance.  8. **Thyroid Dysfunction**: Autoimmune thyroid diseases like Hashimoto's thyroiditis and Graves' disease can lead to symptoms such as fatigue, weight changes, and changes in heart rate.  9. **Hair Loss**: Conditions like alopecia areata, an autoimmune disease, can cause sudden hair loss.  10. **Swelling and Inflammation**: Inflammatory markers may be elevated in autoimmune diseases, leading to symptoms such as swelling, redness, and warmth in affected areas.  It's important to note that these symptoms can be indicative of various medical conditions, not just autoimmune diseases. Proper diagnosis often requires a comprehensive evaluation by a healthcare professional, including a thorough medical history, physical examination, and possibly laboratory tests or imaging studies. |
| 1. What harm do autoimmune diseases cause? | Autoimmune diseases occur when the body's immune system mistakenly attacks its own tissues, thinking they are foreign invaders. This can lead to various harmful effects depending on the specific autoimmune disease and the tissues/organs affected. Some common harms caused by autoimmune diseases include:  1. Inflammation: The immune system's attack on healthy tissues can trigger inflammation, leading to pain, swelling, redness, and loss of function in the affected areas.  2. Tissue Damage: Continued attack by the immune system can cause significant damage to the targeted tissues or organs. For example, in autoimmune diseases like rheumatoid arthritis, the joints can become damaged, leading to chronic pain and disability.  3. Organ Dysfunction: When autoimmune diseases affect vital organs such as the kidneys, liver, or heart, they can impair their function and potentially lead to organ failure. For instance, autoimmune hepatitis can result in liver damage and eventually liver failure.  4. Systemic Effects: Some autoimmune diseases can have systemic effects, impacting multiple organs or systems in the body. For example, systemic lupus erythematosus (SLE) can affect various organs, including the skin, joints, kidneys, and cardiovascular system.  5. Increased Susceptibility to Infections: In some cases, the immune system's focus on attacking the body's own tissues can weaken its ability to fight off infections, making individuals more susceptible to bacterial, viral, or fungal infections.  6. Risk of Complications: Autoimmune diseases can also increase the risk of developing other health complications. For example, individuals with certain autoimmune diseases may have an increased risk of cardiovascular diseases, osteoporosis, or certain cancers.  Overall, autoimmune diseases can significantly impact an individual's quality of life and may require ongoing management and treatment to control symptoms, prevent complications, and reduce the risk of long-term damage. |
| **Topic: Report Interpretation** | |
| 1. Male patient, 66 years old. Test results show anti-cyclic citrullinated peptide antibody (CCP) 186.6 RU/mL (reference range ≤5 RU/mL), anti-mutated citrullinated vimentin antibody (MCV) 911 U/mL (reference range <20 U/mL), and positive anti-keratin antibody (AKA) (reference range negative). What disease should be considered and which specialty should the patient consult? | Based on the test results showing elevated levels of anti-cyclic citrullinated peptide antibody (CCP), anti-mutated citrullinated vimentin antibody (MCV), and positive anti-keratin antibody (AKA), along with the patient's age of 66, a likely diagnosis to consider would be rheumatoid arthritis (RA). These antibodies are commonly associated with RA, particularly CCP and MCV antibodies, which are highly specific for this condition.  Rheumatoid arthritis is a chronic inflammatory autoimmune disorder that primarily affects the joints, causing pain, stiffness, and swelling. It can also affect other organs and systems in the body.  Given the complexity and chronic nature of rheumatoid arthritis, the patient should consult with a rheumatologist. A rheumatologist is a specialist who is trained in the diagnosis and treatment of autoimmune and musculoskeletal disorders, including RA. They can provide comprehensive care and develop a personalized treatment plan to manage the patient's symptoms and improve their quality of life. |
| 1. Female patient, 45 years old. The test report shows positive antinuclear antibody test (fluorescence method), with a fine granular speckled nuclear pattern, titer at 1:1000 positive. Anti-U1RNP/Sm antibody negative, anti-Sm antibody negative, anti-Scl-70 antibody negative, anti-Pm-scl antibody negative, anti-Jo-1 antibody negative, anti-CENP-B antibody negative, anti-PCNA antibody negative, anti-dsDNA antibody (imprinting method) negative, anti-nucleosome antibody negative, anti-histone antibody negative, anti-ribosomal P protein antibody negative, anti-mitochondrial M2 antibody negative, positive anti-SSA antibody, positive anti-SSB antibody, positive anti-Ro-52 antibody, rheumatoid factor 5.6 IU/mL (reference range <20 IU/mL), anti-ds-DNA antibody (ELISA) 0.001 IU/mL (reference range <100 IU/mL). What disease should be considered and which specialty should the patient consult? | Based on the test results provided, the patient presents with positive antinuclear antibodies (ANA) with a fine granular speckled nuclear pattern at a titer of 1:1000, along with positive anti-SSA, anti-SSB, and anti-Ro-52 antibodies. These findings are indicative of systemic lupus erythematosus (SLE), an autoimmune disease.  The patient should be referred to a rheumatologist for further evaluation and management. Rheumatologists specialize in diagnosing and treating autoimmune and inflammatory conditions, including SLE. They can conduct a comprehensive assessment, including a review of the patient's medical history, physical examination, and additional tests if needed, to confirm the diagnosis and develop an appropriate treatment plan tailored to the patient's needs. |
| 1. Female patient, 49 years old. The test report shows positive antinuclear antibody test (fluorescence method), with a mitotic pattern, titer at 1:1000 positive. Anti-U1RNP/Sm antibody negative, anti-Sm antibody negative, anti-Scl-70 antibody negative, anti-Pm-scl antibody negative, anti-Jo-1 antibody negative, positive anti-CENP-B antibody, negative anti-PCNA antibody, negative anti-dsDNA antibody (imprinting method), negative anti-nucleosome antibody, negative anti-histone antibody, negative anti-ribosomal P protein antibody, weakly positive anti-mitochondrial M2 antibody, negative anti-SSA antibody, negative anti-SSB antibody, negative anti-Ro-52 antibody, rheumatoid factor 4.4 IU/mL (reference range <20 IU/mL), anti-ds-DNA antibody (ELISA) 12.21 IU/mL (reference range <100 IU/mL). What disease should be considered and which specialty should the patient consult? | Based on the test results provided, the patient has a positive antinuclear antibody (ANA) test with a mitotic pattern and a titer of 1:1000. Additionally, they have positive anti-CENP-B antibodies and weakly positive anti-mitochondrial M2 antibodies. However, other specific autoantibodies associated with various autoimmune diseases such as systemic lupus erythematosus (SLE), systemic sclerosis (SSc), polymyositis, dermatomyositis, and Sjögren's syndrome are negative or weakly positive.  Given these findings, the patient's symptoms and medical history would need to be taken into consideration to arrive at a definitive diagnosis. However, based solely on the laboratory results provided, the presence of a high-titer ANA with a mitotic pattern along with positive anti-CENP-B antibodies suggests the possibility of CREST syndrome, also known as limited cutaneous systemic sclerosis (lcSSc). CREST syndrome is a subtype of systemic sclerosis characterized by calcinosis, Raynaud's phenomenon, esophageal dysmotility, sclerodactyly, and telangiectasia. Anti-CENP-B antibodies are frequently associated with this condition.  However, it's important to note that the weakly positive anti-mitochondrial M2 antibodies are more commonly associated with primary biliary cholangitis (PBC) rather than CREST syndrome. Therefore, further clinical evaluation, including a detailed medical history, physical examination, and possibly additional diagnostic tests, would be necessary to confirm the diagnosis and determine the appropriate course of action.  The patient should consult a rheumatologist, as rheumatologists specialize in diagnosing and managing autoimmune and inflammatory conditions like CREST syndrome and systemic sclerosis. Additionally, given the potential involvement of other organs such as the gastrointestinal tract or liver, a multidisciplinary approach involving gastroenterologists or hepatologists may also be warranted. |
| 1. Female patient, 82 years old. The test report shows negative anti-Ro-52 antibody, negative anti-EJ antibody, negative anti-PL-12 antibody, positive anti-PL-7 antibody, negative anti-SRP antibody, negative anti-Jo-1 antibody, negative anti-PM-SCl75 antibody, negative anti-PM-SCl100 antibody, negative anti-Ku antibody, negative anti-Mi-2 antibody. What disease should be considered and which specialty should the patient consult? | Based on the test results provided, the positive anti-PL-7 antibody indicates a possibility of antisynthetase syndrome, which is a rare autoimmune disease characterized by inflammatory myopathy, interstitial lung disease, arthritis, Raynaud's phenomenon, and skin involvement. Given the complexity and multisystem nature of this condition, the patient should be referred to a rheumatologist for further evaluation, management, and treatment. |
| 1. Female patient, 47 years old. The test report shows positive antinuclear antibody test (fluorescence method), with a nuclear envelope pattern, titer at 1:1000 positive. What disease should be considered and which specialty should the patient consult? | A positive antinuclear antibody (ANA) test with a nuclear envelope pattern and a titer of 1:1000 suggests the possibility of an autoimmune disease, particularly systemic lupus erythematosus (SLE). However, it's important to note that an ANA test alone cannot diagnose a specific autoimmune disease; further evaluation, including clinical assessment and additional laboratory tests, is necessary for a definitive diagnosis.  Given these findings, the patient should consult with a rheumatologist. Rheumatologists specialize in the diagnosis and treatment of autoimmune and inflammatory diseases, including SLE and other conditions associated with positive ANA tests. They can conduct a comprehensive evaluation, order additional tests if needed, and develop a treatment plan tailored to the patient's individual needs. |
| 1. Female patient, 49 years old. The test report shows positive antinuclear antibody test (fluorescence method), with a fine granular speckled nuclear pattern, titer at 1:3200 positive; spindle pattern, 1:320 positive; nuclear envelope pattern, 1:320 positive. Anti-U1RNP/Sm antibody negative, anti-Sm antibody negative, anti-Scl-70 antibody negative, anti-Pm-scl antibody negative, anti-Jo-1 antibody negative, positive anti-CENP-B antibody, negative anti-PCNA antibody, negative anti-dsDNA antibody (imprinting method), negative anti-nucleosome antibody, negative anti-histone antibody, negative anti-ribosomal P protein antibody, negative anti-mitochondrial M2 antibody, negative anti-mitochondrial M4 antibody, positive anti-mitochondrial M2 antibody, positive anti-SSA antibody, negative anti-SSB antibody, positive anti-Ro-52 antibody, rheumatoid factor 2.1 IU/mL (reference range <20 IU/mL), anti-ds-DNA antibody (ELISA) 5.323 IU/mL (reference range <100 IU/mL). What disease should be considered and which specialty should the patient consult? | Based on the test results provided, the patient's positive antinuclear antibody (ANA) test with a fine granular speckled nuclear pattern, along with positive anti-CENP-B, anti-mitochondrial M2, anti-SSA, and anti-Ro-52 antibodies, suggests a diagnosis of CREST syndrome, also known as limited systemic sclerosis. CREST syndrome is a subtype of systemic sclerosis characterized by the presence of calcinosis, Raynaud's phenomenon, esophageal dysmotility, sclerodactyly, and telangiectasia.  Given the complexity of CREST syndrome and its multi-organ involvement, the patient should consult a rheumatologist for further evaluation, management, and treatment. Rheumatologists specialize in the diagnosis and treatment of autoimmune and inflammatory diseases, including systemic sclerosis and its various subtypes. They are equipped to provide comprehensive care and coordinate with other specialists as needed to address the diverse manifestations of the disease. |
| 1. Male patient, 36 years old. Test results show anti-cyclic citrullinated peptide antibody (CCP) 50.6 RU/mL (reference range ≤5 RU/mL), anti-mutated citrullinated vimentin antibody (MCV) 10.2 U/mL (reference range <20 U/mL), negative anti-keratin antibody (AKA) (reference range negative). What disease should be considered and which specialty should the patient consult? | Based on the test results showing elevated levels of anti-cyclic citrullinated peptide antibody (CCP) and anti-mutated citrullinated vimentin antibody (MCV), along with a negative result for anti-keratin antibody (AKA), the patient should be considered for a diagnosis of rheumatoid arthritis (RA). These autoantibodies are commonly associated with RA, particularly CCP antibodies, which have a high specificity for the disease. MCV antibodies are also indicative of RA, though they are less specific compared to CCP antibodies.  Given the potential diagnosis of rheumatoid arthritis, the patient should consult a rheumatologist for further evaluation, management, and treatment. Rheumatologists specialize in the diagnosis and treatment of autoimmune and inflammatory conditions, including RA. They can provide comprehensive care tailored to the patient's specific needs, including medication management, lifestyle recommendations, and monitoring for disease progression. |
| 1. Female patient, 44 years old. Test results show anti-cyclic citrullinated peptide antibody (CCP) 2.3 RU/mL (reference range ≤5 RU/mL), anti-mutated citrullinated vimentin antibody (MCV) 195.2 U/mL (reference range <20 U/mL), negative anti-keratin antibody (AKA) (reference range negative). What disease should be considered and which specialty should the patient consult? | Based on the patient's test results, particularly the elevated levels of anti-CCP and anti-MCV antibodies, along with the absence of anti-keratin antibody, rheumatoid arthritis (RA) should be considered as a potential diagnosis. These antibodies are commonly associated with RA, an autoimmune disorder that primarily affects the joints.  Given the complexity and management of RA, the patient should consult with a rheumatologist. Rheumatologists specialize in diagnosing and treating autoimmune and inflammatory conditions like RA. They have the expertise to conduct a thorough evaluation, recommend appropriate treatment options, and provide ongoing care to manage the disease effectively. |
| 1. Female patient, 25 years old. The test report shows positive antinuclear antibody test (fluorescence method), with a homogeneous pattern, titer at 1:3200 positive. What disease should be considered and which specialty should the patient consult? | Based on the positive antinuclear antibody (ANA) test with a homogeneous pattern and a titer of 1:3200, several autoimmune diseases should be considered, including:  1. Systemic Lupus Erythematosus (SLE)  2. Rheumatoid Arthritis (RA)  3. Sjögren's Syndrome  4. Systemic Sclerosis (Scleroderma)  5. Mixed Connective Tissue Disease (MCTD)  6. Autoimmune Hepatitis  Given the complexity and potential multisystem involvement of these conditions, the patient should consult a rheumatologist. Rheumatologists specialize in the diagnosis and treatment of autoimmune and musculoskeletal diseases, including those mentioned above. They can conduct a thorough evaluation, order additional tests if necessary, and develop a comprehensive treatment plan tailored to the patient's specific condition. |
| 1. Male patient, 61 years old. The test report shows positive perinuclear anti-neutrophil cytoplasmic antibody (pANCA), negative cytoplasmic anti-neutrophil cytoplasmic antibody (cANCA), anti-myeloperoxidase antibody (Anti-MPO) 10.5 RU/mL (reference range <20 U/mL), anti-proteinase 3 antibody 0.001 RU/mL (reference range <20 U/mL). What disease should be considered and which specialty should the patient consult? | Based on the test results showing positive perinuclear anti-neutrophil cytoplasmic antibody (pANCA) and negative cytoplasmic anti-neutrophil cytoplasmic antibody (cANCA), along with the levels of anti-myeloperoxidase antibody (Anti-MPO) and anti-proteinase 3 antibody falling within the reference ranges, the condition that should be considered is **microscopic polyangiitis (MPA)**. MPA is a type of small vessel vasculitis that primarily affects the small blood vessels in various organs, including the kidneys, lungs, and skin.  Given the complexity and multi-organ involvement of MPA, the patient should consult a **rheumatologist** for further evaluation, diagnosis, and management. Rheumatologists specialize in diagnosing and treating autoimmune and inflammatory diseases, including vasculitis like MPA. They can conduct a comprehensive assessment, order additional tests if necessary, and develop a personalized treatment plan tailored to the patient's needs. Early intervention and appropriate management are crucial in improving outcomes for patients with MPA. |
| 1. Male patient, 67 years old. The test report shows negative perinuclear anti-neutrophil cytoplasmic antibody (pANCA), positive cytoplasmic anti-neutrophil cytoplasmic antibody (cANCA), anti-myeloperoxidase antibody (Anti-MPO) 0.001 RU/mL (reference range <20 U/mL), anti-proteinase 3 antibody 96.5 RU/mL (reference range <20 U/mL). What disease should be considered and which specialty should the patient consult? | Based on the test results showing positive cANCA and elevated anti-proteinase 3 antibody levels, along with negative pANCA and normal anti-myeloperoxidase antibody levels, the condition that should be considered is Granulomatosis with Polyangiitis (GPA), formerly known as Wegener's granulomatosis. This autoimmune disorder typically affects the small to medium-sized blood vessels and is characterized by inflammation of the blood vessels (vasculitis), which can lead to damage in various organs, particularly the lungs, kidneys, and upper respiratory tract.  Given the complexity and multi-system involvement of GPA, the patient should consult with a Rheumatologist, who specializes in the diagnosis and management of autoimmune and inflammatory disorders. The Rheumatologist will further evaluate the patient's symptoms, perform additional tests if necessary, and develop a treatment plan tailored to the individual's needs. Early diagnosis and appropriate management are crucial in improving outcomes for patients with GPA. |
| 1. Female patient, 54 years old, the test report shows negative mitochondrial antibody M2, negative anti-pyruvate dehydrogenase complex antibody, negative anti-acidic phosphoprotein 100 antibody, negative anti-promyelocytic leukemia antibody, negative anti-nuclear envelope glycoprotein antibody, negative anti-Ro-52 antibody, negative anti-liver kidney microsome-1 antibody, negative anti-soluble liver antigen/liver pancreas antigen, positive smooth muscle antibody. What disease should be considered? | Based on the test results provided, the positive smooth muscle antibody suggests the possibility of autoimmune hepatitis (AIH), particularly type 1 AIH. Smooth muscle antibodies (SMA) are commonly associated with autoimmune liver diseases, including AIH. However, it's important to note that a definitive diagnosis cannot be made solely based on antibody tests. Additional clinical information, such as symptoms, medical history, and further diagnostic evaluations, including liver function tests and imaging studies, would be necessary to confirm the diagnosis of AIH. It's also crucial to consider other differential diagnoses and consult with a hepatologist or gastroenterologist for further evaluation and management. |
| 1. Female patient, test report shows positive antinuclear antibody test (fluorescence method), with a fine granular speckled nuclear pattern, titer at 1:3200 positive; cytoplasmic granular pattern, 1:320 positive. Positive anti-U1RNP/Sm antibody, positive anti-Sm antibody, negative anti-Scl-70 antibody, negative anti-Pm-scl antibody, negative anti-Jo-1 antibody, negative anti-CENP-B antibody, negative anti-PCNA antibody, positive anti-dsDNA antibody (imprinting method), positive anti-nucleosome antibody, positive anti-histone antibody, negative anti-ribosomal P protein antibody, positive anti-SSA antibody, negative anti-SSB antibody, positive anti-Ro-52 antibody, rheumatoid factor 5.6 IU/mL (reference range <20 IU/mL), anti-ds-DNA antibody (ELISA) 327.323 IU/mL (reference range <100 IU/mL). What disease should be considered and which specialty should the patient consult? | Based on the patient's test results, they likely have systemic lupus erythematosus (SLE), given the presence of positive antinuclear antibodies (ANA) with a high titer (1:3200), along with specific autoantibodies such as anti-dsDNA, anti-U1RNP/Sm, anti-Sm, anti-nucleosome, anti-histone, anti-SSA, and anti-Ro-52 antibodies. The positive rheumatoid factor and elevated anti-ds-DNA antibody further support the diagnosis of SLE.  The patient should consult a rheumatologist, who specializes in the diagnosis and management of autoimmune diseases like SLE. A rheumatologist can provide comprehensive care and treatment options tailored to the patient's specific needs. It's important for the patient to seek prompt medical attention and establish a treatment plan to manage their condition effectively and improve their quality of life. |
| **Topic: Diagnose** | |
| 1. What laboratory tests are needed to help doctors diagnose if a patient has an autoimmune disease? | Diagnosing autoimmune diseases often involves a combination of clinical evaluation, medical history, physical examination, and laboratory testing. While specific tests may vary depending on the suspected autoimmune condition, here are some common laboratory tests used in the diagnostic process:  1. **Complete Blood Count (CBC)**: This test provides information about the number and types of blood cells. Changes in the CBC, such as anemia or abnormal white blood cell counts, can indicate an underlying autoimmune disorder.  2. **Inflammatory Markers**: Tests such as C-reactive protein (CRP) and erythrocyte sedimentation rate (ESR) measure inflammation levels in the body, which can be elevated in autoimmune diseases.  3. **Autoantibody Testing**: These tests detect antibodies that target the body's own tissues and organs, which are characteristic of many autoimmune diseases. Examples include:  - Antinuclear Antibody (ANA): Commonly used to screen for systemic autoimmune diseases like lupus.  - Rheumatoid Factor (RF) and Anti-cyclic Citrullinated Peptide (anti-CCP): Associated with rheumatoid arthritis.  - Anti-double-stranded DNA (anti-dsDNA) and anti-Smith (anti-Sm) antibodies: Found in lupus.  - Anti-thyroid antibodies: Thyroid peroxidase antibody (TPOAb) and thyroglobulin antibody (TgAb) are associated with autoimmune thyroid disorders like Hashimoto's thyroiditis and Graves' disease.  4. **Organ-Specific Autoantibodies**: Depending on the suspected autoimmune disease, specific autoantibody tests may be ordered to target organs or tissues affected by the condition. Examples include anti-glomerular basement membrane (anti-GBM) antibodies in Goodpasture syndrome and anti-islet cell antibodies in type 1 diabetes.  5. **Immunoglobulin Levels**: Measurement of immunoglobulin levels, such as IgG, IgA, and IgM, can provide insights into immune system function and may be altered in certain autoimmune disorders.  6. **Complement Levels**: Complement proteins play a role in immune responses and inflammation. Abnormal levels may be indicative of certain autoimmune conditions, such as lupus nephritis.  7. **Specialized Tests**: Depending on the suspected autoimmune disease and clinical presentation, additional tests may be warranted. These could include tests for specific antibodies, cytokines, or genetic markers associated with particular conditions.  It's important to note that the interpretation of these tests often requires clinical correlation and expertise, and results should be interpreted in the context of the patient's overall clinical presentation and medical history. Additionally, not all autoimmune diseases have specific diagnostic tests, and diagnosis may require a combination of tests and evaluation by specialists in rheumatology, endocrinology, immunology, or other relevant fields. |
| 1. Does a positive result in autoantibody testing necessarily mean that the patient has an autoimmune disease? | Not necessarily. While a positive result in autoantibody testing can indicate the presence of autoantibodies in the patient's blood, it does not always mean that the patient has an autoimmune disease. Autoantibodies are antibodies produced by the immune system that mistakenly target and attack the body's own tissues, which is a hallmark of autoimmune diseases. However, autoantibodies can also be found in individuals without autoimmune diseases, such as in healthy individuals or those with other conditions.  Interpreting autoantibody test results requires careful consideration of the patient's clinical symptoms, medical history, and other laboratory findings. Additional diagnostic tests and evaluations may be needed to confirm or rule out the presence of an autoimmune disease. Therefore, while a positive result in autoantibody testing can raise suspicion for an autoimmune disease, it is not sufficient for a definitive diagnosis on its own. |
| 1. Does a positive result in antinuclear antibody testing necessarily mean that the patient has an autoimmune disease? | Not necessarily. While a positive result in antinuclear antibody (ANA) testing is often associated with autoimmune diseases, it doesn't definitively confirm the presence of an autoimmune condition on its own. ANA testing detects the presence of antibodies that target components within the nucleus of cells. These antibodies are commonly found in autoimmune diseases such as systemic lupus erythematosus (SLE), rheumatoid arthritis, and Sjögren's syndrome, among others.  However, ANA positivity can also occur in individuals without autoimmune diseases. Factors such as age, infections, medications, and even genetics can lead to a positive ANA result. Additionally, some people may have positive ANA tests without any apparent health issues, which is known as an "ANA-positive healthy individual."  Therefore, while a positive ANA test can indicate the need for further investigation into autoimmune diseases, it should be interpreted in the context of the patient's clinical presentation, medical history, and other diagnostic tests. Consulting with a rheumatologist or an immunologist can help in making a more accurate diagnosis and determining the appropriate course of action for the patient. |
| 1. Is gout an autoimmune disease? | No, gout is not an autoimmune disease. It's a form of inflammatory arthritis caused by the deposition of uric acid crystals in the joints, leading to inflammation, pain, and swelling. Autoimmune diseases occur when the immune system mistakenly attacks the body's own tissues, whereas gout results from metabolic disturbances, particularly elevated levels of uric acid in the blood, which can occur due to various factors such as diet, genetics, and certain medical conditions. However, some autoimmune diseases can predispose individuals to develop secondary gout due to factors like inflammation and tissue damage. |
| 1. I am a patient with Sjögren's syndrome, why do I need an annual chest CT scan? | As a patient with Sjögren's syndrome, your healthcare provider may recommend an annual chest CT scan for several reasons:  1. **Interstitial Lung Disease (ILD) Monitoring:** Sjögren's syndrome can lead to interstitial lung disease, which involves inflammation and scarring of the lung tissue. Regular chest CT scans can help monitor for any changes in the lungs, such as the progression of ILD or the development of complications like pulmonary fibrosis.  2. **Infection Detection:** People with Sjögren's syndrome may have a weakened immune system, making them more susceptible to infections. A chest CT scan can help detect any signs of infection in the lungs, such as pneumonia, which may require prompt treatment.  3. **Lymphoma Surveillance:** Sjögren's syndrome increases the risk of developing lymphoma, a type of cancer that affects the lymphatic system. Annual chest CT scans can help monitor for any signs of lymphadenopathy (enlarged lymph nodes) or other abnormalities in the chest that may indicate the presence of lymphoma.  4. **Evaluation of Other Organ Involvement:** Sjögren's syndrome can affect multiple organs, including the lungs, heart, and kidneys. Regular chest CT scans can help assess the extent of organ involvement and detect any complications or abnormalities that may arise.  Overall, annual chest CT scans play a crucial role in the ongoing management and monitoring of Sjögren's syndrome, helping to detect and manage potential complications early on, thereby improving long-term outcomes and quality of life. Your healthcare provider will consider your individual medical history and specific risk factors when determining the need for chest CT scans and other monitoring tests. |
| 1. Positive antinuclear antibodies, does a higher titer indicate more severe disease? | As the chief physician of the laboratory department, I can provide some insight into this question. Antinuclear antibodies (ANAs) are autoantibodies that target components of the cell nucleus. They are often used as markers for autoimmune diseases such as systemic lupus erythematosus (SLE) and rheumatoid arthritis (RA).  Regarding the correlation between ANA titers and disease severity, it's important to note that the relationship is not always straightforward. While a higher ANA titer can sometimes indicate a more severe or active autoimmune disease, it's not always the case. ANA titers can fluctuate over time and can vary between individuals with the same condition.  In some cases, individuals with low ANA titers may have more severe symptoms, while others with high titers may have milder symptoms or even be asymptomatic. Additionally, the presence of specific patterns or types of ANAs, along with other clinical and laboratory findings, is often more informative than the titer alone when assessing disease severity and activity.  Therefore, while ANA titers can provide valuable information, they should be interpreted in the context of the patient's overall clinical presentation and other laboratory findings. Consulting with a rheumatologist or another specialist experienced in autoimmune diseases can help in accurately assessing and managing patients with positive ANAs. |
| **Topic: Prevention and Treatment** | |
| 1. What are the high-risk factors for autoimmune diseases? | Autoimmune diseases occur when the body's immune system mistakenly attacks its own tissues. There are various factors that can increase the risk of developing autoimmune diseases. Some of the high-risk factors include:  1. **Genetics**: A family history of autoimmune diseases can predispose individuals to developing similar conditions. Certain genes are associated with an increased susceptibility to autoimmune diseases.  2. **Gender**: Many autoimmune diseases are more common in women than in men. Hormonal differences between genders may play a role in this disparity.  3. **Environmental Factors**: Exposure to certain environmental triggers, such as infections, toxins, or stress, can contribute to the development of autoimmune diseases. For example, infections like Epstein-Barr virus have been linked to the onset of autoimmune conditions like lupus.  4. **Age**: While autoimmune diseases can occur at any age, some conditions tend to develop during specific stages of life. For instance, rheumatoid arthritis often manifests in middle age, while type 1 diabetes is more common in childhood or adolescence.  5. **Hormonal Factors**: Hormonal changes, such as those occurring during pregnancy or menopause, can influence the immune system and contribute to the development or exacerbation of autoimmune diseases.  6. **Chronic Inflammation**: Conditions characterized by chronic inflammation, such as inflammatory bowel disease or chronic infections, may increase the risk of developing autoimmune diseases.  7. **Smoking**: Smoking has been associated with an increased risk of several autoimmune diseases, including rheumatoid arthritis, lupus, and multiple sclerosis.  8. **Obesity**: There is growing evidence linking obesity to an increased risk of autoimmune diseases. Adipose tissue produces inflammatory cytokines that can contribute to autoimmune inflammation.  9. **Diet**: Although the role of diet in autoimmune diseases is complex and not fully understood, certain dietary factors, such as gluten or dairy, may trigger or exacerbate symptoms in individuals with specific autoimmune conditions like celiac disease or rheumatoid arthritis.  It's important to note that while these factors can increase the risk of autoimmune diseases, not everyone with these risk factors will develop an autoimmune condition. Additionally, the interplay between genetics, environment, and other factors in autoimmune disease development is still an area of active research. |
| 1. Should autoantibodies be tested during routine physical examinations? | Autoantibodies are antibodies that mistakenly target and attack the body's own tissues. Testing for autoantibodies during routine physical examinations can be valuable in certain cases, especially if there is a suspicion of autoimmune disease based on symptoms or medical history.  In general, routine testing for autoantibodies may not be necessary for every individual during a routine physical examination unless there are specific symptoms or risk factors suggesting autoimmune conditions. However, in certain situations, such as when there is a family history of autoimmune diseases or when symptoms suggestive of autoimmune disorders are present, it may be prudent to include autoantibody testing as part of the routine examination.  Ultimately, the decision to test for autoantibodies should be made on a case-by-case basis by the healthcare provider, taking into consideration the individual's medical history, symptoms, and risk factors. If there are concerns about autoimmune conditions, discussing these with your healthcare provider can help determine whether autoantibody testing is appropriate for you. |
| 1. Should antinuclear antibodies be tested during routine physical examinations? | Antinuclear antibodies (ANA) testing is not typically included in routine physical examinations unless there are specific indications. ANA testing is primarily used to aid in the diagnosis of autoimmune diseases such as systemic lupus erythematosus (SLE), rheumatoid arthritis, and Sjögren's syndrome. These conditions often present with symptoms such as joint pain, fatigue, and inflammation, prompting a physician to order ANA testing.  Including ANA testing in routine physical examinations without clear symptoms or risk factors for autoimmune diseases can lead to unnecessary testing and potential anxiety for patients due to false positive results. However, if a patient presents with symptoms suggestive of autoimmune disease or has a family history of such conditions, then ANA testing may be warranted and appropriate. It's always best to discuss the necessity of any tests with a healthcare provider based on individual circumstances. |
| 1. Do autoimmune diseases have a hereditary component? My mother has Sjögren's syndrome, how can I prevent myself from getting sick? | Yes, autoimmune diseases often have a hereditary component, meaning they can run in families. If your mother has Sjögren's syndrome, there may be a slightly higher risk for you to develop it as well, although it's not guaranteed. While you can't completely prevent autoimmune diseases, there are some steps you can take to potentially lower your risk or manage symptoms:  1. **Regular Check-ups:** Visit your healthcare provider regularly for check-ups and screenings. Early detection and treatment of autoimmune diseases can help manage symptoms and improve outcomes.  2. **Healthy Lifestyle:** Maintain a healthy lifestyle with regular exercise, a balanced diet rich in fruits and vegetables, and adequate sleep. This can help support your immune system and overall health.  3. **Stress Management:** High levels of stress can potentially trigger or exacerbate autoimmune symptoms. Practice stress-reducing techniques such as meditation, deep breathing exercises, or hobbies you enjoy.  4. **Avoid Smoking:** If you smoke, quitting can reduce your risk of developing autoimmune diseases, as smoking can increase inflammation and disrupt the immune system.  5. **Limit Alcohol:** Excessive alcohol consumption can also negatively impact the immune system. Limiting alcohol intake or avoiding it altogether may be beneficial.  6. **Stay Informed:** Learn about the symptoms and warning signs of autoimmune diseases, and seek medical attention if you experience any concerning symptoms. Early intervention can make a significant difference in managing these conditions.  7. **Genetic Counseling:** Consider speaking with a genetic counselor to better understand your risk factors based on your family history. They can provide personalized recommendations and support.  Remember, while these steps may help lower your risk or manage symptoms, there's no foolproof way to prevent autoimmune diseases entirely. It's essential to stay proactive about your health and work closely with healthcare professionals for personalized guidance and support. |
| 1. I have systemic lupus erythematosus, what should I pay attention to in daily life? | As someone with systemic lupus erythematosus (SLE), there are several key aspects of daily life to pay attention to in order to manage your condition effectively:  1. **Medication Adherence**: It's crucial to take your prescribed medications consistently and as directed by your healthcare provider. This may include immunosuppressants, anti-inflammatory drugs, and other medications to manage symptoms and prevent flare-ups.  2. **Sun Protection**: Sun exposure can trigger lupus flares, so it's important to protect your skin by wearing sunscreen with a high SPF, protective clothing, and hats when outdoors. Additionally, try to avoid prolonged sun exposure, especially during peak hours.  3. **Stress Management**: Stress can exacerbate lupus symptoms, so finding healthy ways to manage stress is essential. This could include relaxation techniques such as deep breathing, meditation, yoga, or engaging in hobbies and activities you enjoy.  4. **Healthy Lifestyle**: Maintaining a balanced diet, getting regular exercise, and prioritizing sufficient sleep can help support overall health and reduce the risk of flare-ups. Aim for a diet rich in fruits, vegetables, whole grains, and lean proteins, and limit processed foods and excessive sugar and sodium intake.  5. **Regular Medical Check-ups**: Stay in close communication with your healthcare provider and attend regular check-ups to monitor your condition, adjust treatment as needed, and address any concerns or changes in symptoms promptly.  6. **Infection Prevention**: Due to the immunosuppressive nature of some lupus medications, it's important to take precautions to prevent infections. Practice good hand hygiene, avoid close contact with sick individuals when possible, and stay up-to-date on vaccinations as recommended by your healthcare provider.  7. **Listen to Your Body**: Pay attention to your body's signals and recognize when you may be experiencing a lupus flare. Rest when needed, pace yourself, and don't hesitate to seek medical attention if you notice worsening symptoms or new concerns.  By prioritizing these aspects of daily life and working closely with your healthcare team, you can better manage systemic lupus erythematosus and improve your overall quality of life. If you have any specific concerns or questions, don't hesitate to discuss them with your healthcare provider. |
| 1. I have systemic lupus erythematosus, and my condition is stable under medication. What indicators should I regularly check? | As a chief physician overseeing laboratory diagnostics for patients with systemic lupus erythematosus (SLE), it's crucial to monitor various indicators to ensure your condition remains stable under medication. Here are some key indicators you should regularly check:  1. **Complete Blood Count (CBC):** This test assesses the levels of different types of blood cells, including red blood cells, white blood cells, and platelets. In SLE patients, abnormalities such as anemia, leukopenia, or thrombocytopenia can occur due to the autoimmune process or medication side effects.  2. **Renal Function Tests:** Monitoring kidney function is essential because lupus nephritis, inflammation of the kidneys caused by SLE, is a common complication. Tests such as serum creatinine, blood urea nitrogen (BUN), and urine protein levels help assess kidney function and detect any signs of renal involvement.  3. **Liver Function Tests:** SLE patients may experience liver involvement, characterized by elevated liver enzymes such as alanine aminotransferase (ALT) and aspartate aminotransferase (AST). Monitoring liver function can help detect liver inflammation or damage caused by the disease or medication.  4. **Autoantibody Testing:** SLE is characterized by the production of autoantibodies, such as antinuclear antibodies (ANA), anti-double-stranded DNA (anti-dsDNA) antibodies, and anti-Smith (anti-Sm) antibodies. Regular monitoring of these autoantibodies can help assess disease activity and guide treatment decisions.  5. **Complement Levels:** SLE can lead to complement system activation and consumption, resulting in decreased levels of complement proteins such as C3 and C4. Monitoring complement levels can provide insights into disease activity and response to treatment.  6. **Inflammatory Markers:** Assessing markers of inflammation, such as erythrocyte sedimentation rate (ESR) and C-reactive protein (CRP), can help evaluate disease activity and monitor response to therapy.  7. **Lipid Profile:** SLE patients may have an increased risk of cardiovascular disease, partly due to the effects of inflammation and medication. Monitoring lipid levels, including total cholesterol, LDL cholesterol, HDL cholesterol, and triglycerides, is important for cardiovascular risk assessment and management.  Regular monitoring of these indicators, along with clinical evaluation and imaging studies as needed, can help ensure timely detection of any disease activity or medication-related complications in patients with systemic lupus erythematosus. It's essential to work closely with your healthcare team to develop a personalized monitoring plan based on your individual needs and medical history. |
| 1. I have systemic lupus erythematosus, and my condition is stable under medication. How often should I go to the hospital for a check-up? | As the chief physician of the laboratory department, I would recommend that you continue regular follow-up appointments with your rheumatologist or primary care physician to monitor your systemic lupus erythematosus (SLE) and ensure that your condition remains stable. The frequency of these check-ups can vary depending on individual factors such as the severity of your SLE, the effectiveness of your current medication regimen, and any other underlying health conditions you may have.  Typically, patients with stable SLE may need to visit the hospital or their healthcare provider for check-ups every three to six months. During these appointments, your healthcare provider will assess your symptoms, monitor any changes in your condition, and adjust your treatment plan if necessary.  However, it's important to note that this recommendation may vary for each individual, so it's crucial to discuss your specific situation with your healthcare provider. They can provide personalized guidance based on your medical history and current health status. Additionally, make sure to adhere to any routine laboratory tests or imaging studies recommended by your healthcare provider to effectively manage your SLE and maintain your overall health and well-being. |
| 1. What are the common medications for autoimmune diseases? | For autoimmune diseases, the choice of medications depends on the specific condition and its severity. However, some common medications used to manage autoimmune diseases include:  1. **Nonsteroidal anti-inflammatory drugs (NSAIDs)**: These are often used to reduce inflammation and relieve pain associated with autoimmune conditions like rheumatoid arthritis and lupus.  2. **Corticosteroids**: These medications, such as prednisone, are powerful anti-inflammatory drugs that can help control inflammation in various autoimmune diseases. They are often used for short-term management due to their potential side effects with long-term use.  3. **Disease-modifying antirheumatic drugs (DMARDs)**: These drugs, including methotrexate, hydroxychloroquine, and sulfasalazine, are commonly used to treat rheumatoid arthritis and other autoimmune conditions. They work by suppressing the immune system to reduce inflammation and slow down the progression of the disease.  4. **Biologic agents**: Biologics are a newer class of medications that target specific components of the immune system involved in autoimmune diseases. Examples include adalimumab, infliximab, etanercept, and rituximab. These drugs are often used when DMARDs are ineffective or poorly tolerated.  5. **Immunosuppressants**: Drugs like azathioprine, cyclosporine, and mycophenolate mofetil suppress the immune system to reduce inflammation and prevent the immune system from attacking the body's own tissues.  6. **Janus kinase (JAK) inhibitors**: These medications, such as tofacitinib, baricitinib, and upadacitinib, target specific enzymes involved in the inflammatory process and are used to treat autoimmune diseases like rheumatoid arthritis and psoriatic arthritis.  7. **Biological response modifiers**: These drugs, like interferons and interleukin inhibitors, target specific molecules involved in the immune response to help regulate the immune system in autoimmune diseases such as multiple sclerosis and psoriasis.  It's important to note that the choice of medication and treatment plan should be tailored to each individual's specific condition, medical history, and response to treatment. Regular monitoring and follow-up with a healthcare provider are essential to ensure the effectiveness and safety of treatment. |
| 1. What are the treatment principles for autoimmune diseases? | The treatment principles for autoimmune diseases generally revolve around managing symptoms, reducing inflammation, and suppressing the immune response to prevent further damage to the body's tissues. Here are some key strategies commonly used:  1. **Immunosuppressive Medications**: These drugs help dampen the overactive immune response responsible for attacking the body's own tissues. Common immunosuppressants include corticosteroids, such as prednisone, and other medications like methotrexate, azathioprine, and cyclosporine.  2. **Anti-inflammatory Medications**: Nonsteroidal anti-inflammatory drugs (NSAIDs) like ibuprofen or naproxen may help alleviate pain and inflammation associated with autoimmune conditions. Additionally, drugs like hydroxychloroquine may be prescribed to reduce inflammation and modulate the immune system.  3. **Biologic Therapies**: Biologic drugs target specific components of the immune system involved in autoimmune diseases. Examples include tumor necrosis factor (TNF) inhibitors (e.g., adalimumab, infliximab), interleukin inhibitors (e.g., tocilizumab, ustekinumab), and B-cell inhibitors (e.g., rituximab).  4. **Disease-Modifying Antirheumatic Drugs (DMARDs)**: These medications can slow the progression of certain autoimmune diseases by suppressing the immune system's attack on the joints and other tissues. Examples include methotrexate, sulfasalazine, and leflunomide.  5. **Corticosteroids**: While primarily used for their anti-inflammatory properties, corticosteroids like prednisone can also suppress the immune system's activity. They are often prescribed to quickly reduce inflammation during disease flares but are usually used at the lowest effective dose for the shortest possible duration due to potential side effects.  6. **Lifestyle Modifications**: Adopting a healthy lifestyle, including regular exercise, a balanced diet, stress management techniques, and adequate sleep, can help support overall health and may improve symptoms in some autoimmune diseases.  7. **Monitoring and Follow-Up**: Regular monitoring of disease activity, medication side effects, and overall health status is crucial. Adjustments to treatment plans may be necessary over time to optimize symptom control while minimizing side effects.  It's important to note that treatment approaches can vary depending on the specific autoimmune disease, its severity, and individual patient factors. Therefore, a personalized treatment plan developed in collaboration with a healthcare provider is essential for effectively managing autoimmune conditions. |
| 1. I have systemic lupus erythematosus and am currently undergoing steroid therapy. What are the side effects of steroids? | As a chief physician specializing in laboratory medicine, I can provide information on the potential side effects of steroid therapy, particularly for someone like you who is undergoing treatment for systemic lupus erythematosus (SLE).  Steroid therapy, commonly used to manage autoimmune conditions like SLE, can indeed be effective, but it also carries a range of potential side effects. These can vary depending on factors such as the dosage, duration of treatment, and individual patient factors. Here are some common side effects to be aware of:  1. **Weight gain**: Steroids can cause fluid retention and increased appetite, leading to weight gain.  2. **Increased risk of infections**: Steroids can suppress the immune system, making individuals more susceptible to infections.  3. **Osteoporosis**: Long-term steroid use can weaken bones, increasing the risk of fractures.  4. **Glaucoma and cataracts**: Steroids can increase the risk of developing eye conditions such as glaucoma and cataracts.  5. **High blood pressure**: Steroids can cause an increase in blood pressure, which may need to be monitored closely.  6. **Diabetes**: Steroids can interfere with blood sugar regulation, leading to elevated blood glucose levels and potentially causing or exacerbating diabetes.  7. **Mood changes**: Some individuals may experience mood swings, irritability, or even psychiatric symptoms such as depression or anxiety.  8. **Adrenal suppression**: Prolonged use of steroids can suppress the body's natural production of cortisol, which can lead to adrenal insufficiency when the medication is discontinued.  9. **Gastrointestinal issues**: Steroids can increase the risk of gastrointestinal problems such as ulcers or gastritis.  10. **Skin changes**: Some individuals may experience acne or thinning of the skin.  It's essential to discuss these potential side effects with your healthcare provider, as they can provide personalized guidance on managing and mitigating these risks. Additionally, regular monitoring through laboratory tests and clinical assessments can help ensure that any adverse effects are detected early and addressed appropriately. |
| 1. I have Sjögren's syndrome and am currently being treated with immunosuppressants (tacrolimus). What are the side effects? | As a chief physician specializing in laboratory medicine, I can provide some insights into the potential side effects of tacrolimus, particularly in the context of managing Sjögren's syndrome.  Tacrolimus is an immunosuppressant medication commonly used to treat various autoimmune conditions, including Sjögren's syndrome. While it can effectively reduce inflammation and suppress the immune system, there are several potential side effects to be aware of:  1. **Increased Risk of Infections:** One of the primary concerns with immunosuppressant therapy is the heightened susceptibility to infections. Tacrolimus can weaken the body's ability to fight off bacteria, viruses, and other pathogens, potentially leading to more frequent or severe infections.  2. **Renal Toxicity:** Long-term use of tacrolimus may impair kidney function, leading to renal toxicity. Monitoring kidney function through regular blood tests is essential to detect any early signs of kidney damage.  3. **Hypertension:** Tacrolimus can elevate blood pressure in some individuals, which may increase the risk of cardiovascular complications. Monitoring blood pressure regularly and managing it with lifestyle modifications or additional medications may be necessary.  4. **Neurological Effects:** Some patients may experience neurological side effects such as headaches, tremors, or confusion while taking tacrolimus. These symptoms should be reported to your healthcare provider for further evaluation.  5. **Gastrointestinal Issues:** Tacrolimus can cause gastrointestinal disturbances, including nausea, vomiting, diarrhea, or abdominal discomfort. These symptoms may improve over time but should be monitored closely.  6. **Hyperglycemia:** Tacrolimus may interfere with glucose metabolism, leading to elevated blood sugar levels. Patients with pre-existing diabetes or those at risk of developing diabetes should monitor their blood sugar levels closely while taking this medication.  7. **Hyperkalemia:** Tacrolimus can also affect potassium levels in the blood, potentially leading to hyperkalemia (high potassium levels). Monitoring electrolyte levels through blood tests is important to prevent complications associated with abnormal potassium levels.  It's crucial to discuss any concerns or potential side effects with your healthcare provider before starting or continuing tacrolimus therapy. Your healthcare team can monitor your condition closely and adjust your treatment regimen as needed to minimize side effects and optimize therapeutic outcomes. Additionally, regular laboratory monitoring, including blood tests, can help detect any early signs of adverse effects and guide treatment decisions. |
| 1. What are the different types of immunosuppressants? | There are several types of immunosuppressants, each with its own mechanisms of action and therapeutic uses. Here are some common types:  1. Corticosteroids: These drugs, such as prednisone and dexamethasone, suppress inflammation and immune responses by inhibiting the production of cytokines and other inflammatory mediators.  2. Calcineurin Inhibitors: Drugs like cyclosporine and tacrolimus inhibit the activity of calcineurin, a protein phosphatase that is crucial for the activation of T cells. By blocking T cell activation, these drugs suppress the immune response.  3. Antimetabolites: Examples include methotrexate, azathioprine, and mycophenolate mofetil. These drugs interfere with the synthesis of DNA and RNA, thereby inhibiting the proliferation of rapidly dividing cells, including activated lymphocytes.  4. Biological Therapies: These drugs are typically monoclonal antibodies or fusion proteins that target specific components of the immune system. Examples include drugs targeting tumor necrosis factor-alpha (e.g., infliximab, adalimumab), interleukin-6 (e.g., tocilizumab), and CD20 on B cells (e.g., rituximab).  5. mTOR Inhibitors: Drugs like sirolimus (rapamycin) and everolimus inhibit the mammalian target of rapamycin (mTOR), a protein kinase involved in cell growth and proliferation. By blocking mTOR signaling, these drugs suppress T cell activation and proliferation.  6. Monoclonal Antibodies: These drugs target specific immune cells or molecules involved in the immune response. Examples include basiliximab and daclizumab, which target the interleukin-2 receptor on T cells to prevent their activation.  These immunosuppressants are used in various combinations and dosages depending on the specific indication, patient factors, and response to treatment. It's important to note that immunosuppressants carry risks of side effects and increased susceptibility to infections, so their use requires careful monitoring and management by healthcare professionals. |
| 1. What dietary considerations should be taken during the use of immunosuppressants? | When it comes to using immunosuppressants, dietary considerations are crucial to ensure the effectiveness of the medication and the overall health of the patient. Here are some key dietary considerations:  1. **Maintain a Balanced Diet**: Emphasize the importance of a balanced diet rich in fruits, vegetables, whole grains, lean proteins, and healthy fats. This provides essential nutrients to support overall health and immune function.  2. **Limit Sodium Intake**: Some immunosuppressants may increase the risk of high blood pressure and fluid retention. Therefore, it's important to advise patients to limit their sodium intake to help manage these risks.  3. **Monitor Potassium Levels**: Certain immunosuppressants can affect potassium levels in the body. Patients should be educated about foods that are high in potassium, such as bananas, oranges, spinach, and potatoes, and advised to consume them in moderation.  4. **Limit Alcohol**: Alcohol can interact with immunosuppressant medications and may increase the risk of liver damage. Patients should be advised to limit or avoid alcohol consumption while taking these medications.  5. **Food Safety**: Patients on immunosuppressants have weakened immune systems, making them more susceptible to infections. Therefore, it's important to emphasize the importance of food safety practices, such as washing hands and thoroughly cooking foods to prevent foodborne illnesses.  6. **Avoid Grapefruit**: Grapefruit and grapefruit juice can interact with certain immunosuppressants, leading to increased levels of the medication in the bloodstream. Patients should be advised to avoid consuming grapefruit or grapefruit products while taking these medications.  7. **Calcium and Vitamin D**: Some immunosuppressants can increase the risk of osteoporosis. Encourage patients to consume adequate amounts of calcium and vitamin D to support bone health. This can be achieved through dietary sources such as dairy products, leafy greens, and fortified foods, or through supplements if necessary.  8. **Consultation with a Dietitian**: For personalized dietary advice, patients can benefit from consulting with a registered dietitian who can help them develop a nutrition plan tailored to their specific needs and medication regimen.  By addressing these dietary considerations, healthcare providers can help optimize the effectiveness of immunosuppressant therapy while promoting the overall health and well-being of patients. |
| 1. What immunosuppressants require monitoring of drug levels during use? | Immunosuppressants are medications used to suppress the immune system, often prescribed to prevent organ rejection after transplantation or to treat autoimmune diseases. Monitoring drug levels is crucial to ensure therapeutic efficacy while minimizing the risk of toxicity. Some common immunosuppressants that require monitoring of drug levels include:  1. **Tacrolimus (Prograf)**: This medication is commonly used to prevent organ rejection after transplantation, particularly in kidney and liver transplants. Therapeutic drug monitoring is essential due to its narrow therapeutic window.  2. **Cyclosporine (Neoral, Sandimmune)**: Similar to tacrolimus, cyclosporine is also used to prevent organ rejection. Monitoring of blood levels is necessary to adjust the dosage and prevent toxicity.  3. **Sirolimus (Rapamune)**: Sirolimus is another immunosuppressant used in transplantation medicine. Monitoring of drug levels is important due to its potential for side effects such as renal dysfunction and hyperlipidemia.  4. **Mycophenolate mofetil (CellCept)**: This medication is often used in combination with other immunosuppressants. Monitoring of mycophenolic acid levels, the active metabolite of mycophenolate mofetil, can help optimize therapy.  5. **Azathioprine (Imuran)**: Azathioprine is commonly used to prevent rejection in organ transplantation and to treat autoimmune diseases. Monitoring of metabolites such as 6-thioguanine nucleotides (6-TGN) and 6-methylmercaptopurine (6-MMP) may be necessary to ensure therapeutic efficacy and safety.  6. **Everolimus (Zortress)**: Like sirolimus, everolimus is an mTOR inhibitor used as an immunosuppressant. Monitoring of blood levels is recommended to adjust dosage and prevent adverse effects.  7. **Belatacept (Nulojix)**: This newer immunosuppressant is used in kidney transplantation. Monitoring of drug levels is important to optimize dosing and minimize rejection risk.  These are just a few examples, and the specific immunosuppressants and monitoring protocols may vary depending on the patient's condition, transplant type, and individual response to therapy. Regular monitoring by healthcare providers is essential to ensure safe and effective immunosuppressive therapy. |
| 1. I was recently diagnosed with systemic lupus erythematosus. How often should I get a check-up during treatment? | As the chief physician overseeing the laboratory department, I'm here to provide guidance regarding your diagnosis of systemic lupus erythematosus (SLE). Treatment and follow-up for SLE vary depending on the severity of your symptoms, the organs affected, and the medications prescribed. Generally, patients with SLE require regular monitoring to assess disease activity, medication efficacy, and potential side effects. Here's a general recommendation for check-up frequency:  1. **Initial Phase:** Following your diagnosis, you may require more frequent visits to closely monitor your condition and adjust treatment accordingly. This phase may involve monthly or bimonthly check-ups initially.  2. **Stable Phase:** Once your symptoms are under control and your condition stabilizes, you may transition to less frequent follow-ups. Typically, patients in a stable phase of SLE may have check-ups every three to six months.  3. **Flare-ups or Changes in Symptoms:** If you experience any flare-ups of symptoms or notice significant changes in your health status, it's essential to contact your healthcare provider promptly. Depending on the severity of the flare-up, additional visits or adjustments to your treatment plan may be necessary.  4. **Medication Monitoring:** Regular monitoring of blood tests, such as complete blood count, kidney function tests, and tests to assess inflammation levels (such as C-reactive protein or erythrocyte sedimentation rate), may be recommended to evaluate the response to treatment and detect any potential side effects.  5. **Individualized Care:** Keep in mind that these recommendations may vary based on your individual circumstances and the recommendations of your healthcare provider. It's essential to maintain open communication with your healthcare team and follow their guidance regarding follow-up care and monitoring.  Remember, managing SLE is often a team effort involving various healthcare professionals, including rheumatologists, primary care physicians, and specialists in other areas as needed. Don't hesitate to reach out to your healthcare provider if you have any questions or concerns about your treatment or follow-up care. |
| 1. What should a patient with gout consider in their daily diet? | For a patient with gout, managing their diet is crucial to reduce the risk of flare-ups and manage symptoms. Here are some dietary considerations:  1. **Limit Purine-Rich Foods:** Purines break down into uric acid, which can exacerbate gout symptoms. Foods high in purines include red meat, organ meats (such as liver and kidneys), shellfish, and certain types of fish (like anchovies, sardines, and mackerel). It's advisable for patients to limit consumption of these foods.  2. **Moderate Alcohol Intake:** Alcohol, particularly beer and spirits, can increase uric acid levels and trigger gout attacks. Encourage patients to drink alcohol in moderation or, ideally, avoid it altogether.  3. **Stay Hydrated:** Drinking plenty of water helps to dilute uric acid in the blood, reducing the risk of crystallization in the joints. Aim for at least 8-10 glasses of water per day.  4. **Choose Low-Purine Alternatives:** Instead of high-purine foods, patients can opt for low-purine alternatives such as low-fat dairy products, tofu, nuts and seeds, fruits, and vegetables. Incorporating more plant-based protein sources can be beneficial.  5. **Control Portion Sizes:** Even low-purine foods should be consumed in moderation. Overeating can still lead to increased uric acid levels.  6. **Maintain a Healthy Weight:** Obesity is a risk factor for gout, as it can lead to higher uric acid levels. Encourage patients to achieve and maintain a healthy weight through a balanced diet and regular exercise.  7. **Be Cautious with Sugary Foods and Beverages:** Some studies suggest a link between high fructose intake and gout. Patients should moderate their consumption of sugary foods and drinks, including soda and processed snacks.  8. **Consider Dietary Supplements:** Certain supplements, such as vitamin C, may help reduce uric acid levels. However, patients should consult with their healthcare provider before starting any new supplements.  It's essential for patients with gout to work closely with their healthcare providers, including dietitians or nutritionists, to develop a personalized dietary plan that suits their individual needs and helps manage their condition effectively. |
| 1. I was recently diagnosed with systemic lupus erythematosus. Should my siblings visit a rheumatology and immunology department? | Yes, it's advisable for your siblings to visit a rheumatology and immunology department, especially if they are experiencing any symptoms or have concerns about their health. Systemic lupus erythematosus (SLE) can have a genetic component, meaning that there may be an increased risk for family members, including siblings, to develop the condition or related autoimmune disorders.  A rheumatologist can assess your siblings' medical history, perform any necessary tests, and provide guidance on whether they should undergo screening or monitoring for autoimmune diseases. Early detection and management are crucial for improving outcomes, so it's essential for them to seek medical advice if they have any concerns. |
| 1. Which patients with positive autoantibody indicators have a higher chance of developing tumors? | Patients with positive autoantibody indicators, particularly those targeting specific tumor-associated antigens, may have a higher likelihood of developing tumors. These autoantibodies can indicate an underlying autoimmune response against cancer cells or precursor cells, which may be present even before the tumor becomes clinically detectable. However, it's essential to note that not all patients with positive autoantibodies will necessarily develop tumors. The risk varies depending on factors such as the specific autoantibodies present, the patient's overall health status, and genetic predispositions. Regular monitoring and further diagnostic evaluations are typically recommended for patients with positive autoantibody indicators to detect and manage any potential tumors at an early stage. |
| **Topic: Prognosis** | |
| 1. I am a patient with rheumatoid arthritis. My anti-mutated citrullinated vimentin antibody test results were 870 U/mL a year ago and 562 U/mL a week ago. Does this indicate that my condition is improving? | As a chief physician specializing in laboratory medicine, I can provide some insights into your question. The anti-mutated citrullinated vimentin (MCV) antibody test is commonly used in the diagnosis and management of rheumatoid arthritis (RA).  A decrease in the MCV antibody levels over time, such as in your case where the levels dropped from 870 U/mL a year ago to 562 U/mL a week ago, can suggest several possibilities:  1. **Improvement in Disease Activity**: A decrease in MCV antibody levels may indicate a reduction in disease activity. In RA, higher antibody levels often correlate with more active disease, so a decrease could suggest that your condition is improving.  2. **Response to Treatment**: It's possible that the decrease in MCV antibody levels is a result of your treatment regimen. If you've been undergoing treatment for RA, such as disease-modifying antirheumatic drugs (DMARDs) or biologic therapies, a decrease in antibody levels could indicate a response to these treatments.  3. **Natural Fluctuations**: Antibody levels can fluctuate naturally over time, so it's essential to consider this possibility. However, a significant decrease like the one you've experienced is more likely to be clinically meaningful rather than just a fluctuation.  4. **Lab Variability**: It's also crucial to consider any potential variability in the laboratory assay used to measure MCV antibody levels. While laboratories strive for accuracy and consistency, there can be slight variations between different assays or even within the same assay over time. However, significant changes in antibody levels like the ones you've described are less likely to be solely due to assay variability.  Overall, a decrease in MCV antibody levels from 870 U/mL to 562 U/mL over the course of a year suggests a positive trend. However, it's essential to interpret these results in conjunction with other clinical and laboratory findings, as well as your overall disease management plan. I would recommend discussing these results with your rheumatologist or healthcare provider to get a comprehensive assessment of your condition and treatment plan. |
| 1. What is the prognosis for autoimmune diseases? | The prognosis for autoimmune diseases can vary widely depending on the specific condition, its severity, and how well it responds to treatment. Some autoimmune diseases can be effectively managed with medication and lifestyle changes, allowing individuals to live relatively normal lives. However, other autoimmune diseases can be more severe and may lead to complications or long-term health issues.  In general, early diagnosis and appropriate treatment are crucial in improving outcomes for individuals with autoimmune diseases. Advances in medical research and therapies have led to better management strategies for many autoimmune conditions, but it's important to recognize that some autoimmune diseases can be chronic and require ongoing care.  Regular monitoring by healthcare providers, adherence to treatment plans, and lifestyle modifications such as maintaining a healthy diet and managing stress can all play important roles in optimizing prognosis and quality of life for individuals with autoimmune diseases. Additionally, ongoing research into the underlying mechanisms of autoimmune diseases may lead to further advancements in treatment and ultimately improve outcomes for affected individuals. |
| 1. Which autoimmune diseases progress rapidly and have a poor prognosis? | Autoimmune diseases vary widely in their progression and prognosis, but some can indeed progress rapidly and carry a poor prognosis. Examples include:  1. **Systemic lupus erythematosus (SLE)**: This autoimmune disease affects multiple organs and can progress rapidly, leading to severe complications such as kidney failure, cardiovascular disease, and central nervous system involvement.  2. **Autoimmune hepatitis**: This condition involves inflammation of the liver due to an autoimmune response. Without proper management, it can progress rapidly to liver failure.  3. **Acute inflammatory demyelinating polyneuropathy (AIDP)**: A subtype of Guillain-Barré syndrome, AIDP can progress rapidly, leading to muscle weakness and paralysis.  4. **Acute disseminated encephalomyelitis (ADEM)**: This autoimmune condition affects the brain and spinal cord, often following an infection or vaccination. It can progress rapidly, causing neurological deficits and even death in severe cases.  5. **Goodpasture's syndrome**: This autoimmune disorder primarily affects the kidneys and lungs, leading to rapid deterioration of kidney function and potentially life-threatening lung hemorrhage.  6. **Fulminant type 1 diabetes**: While diabetes is typically considered a chronic condition, the fulminant type presents suddenly and progresses rapidly, leading to severe insulin deficiency and diabetic ketoacidosis.  Early diagnosis and aggressive treatment are essential in managing these conditions to mitigate their progression and improve outcomes. |

# Supplementary Table 1b: ChatGPT-4.0 Responses to Questions

| **Question** | **ChatGPT-4.0 Response** |
| --- | --- |
| **Topic: Concept** | |
| 1. What is an autoimmune disease? | An autoimmune disease occurs when the body's immune system mistakenly attacks and damages its own tissues and organs. Under normal circumstances, the immune system protects the body from diseases and infections by targeting and destroying foreign invaders like bacteria and viruses. However, in autoimmune diseases, the immune system fails to distinguish between foreign cells and the body’s own cells, leading to an immune response against the body's normal components.  There are many types of autoimmune diseases, and they can affect various parts of the body. Some common examples include type 1 diabetes, where the immune system attacks the insulin-producing cells of the pancreas; rheumatoid arthritis, where it targets the joints; and multiple sclerosis, where it attacks the nervous system. The exact cause of autoimmune diseases is not fully understood, but they are believed to result from a combination of genetic, environmental, and possibly hormonal factors. |
| 1. What diseases are included in autoimmune diseases? | Autoimmune diseases are conditions in which the immune system mistakenly attacks the body's own tissues. There are many autoimmune diseases, varying widely in symptoms and severity. Here are some of the more common and well-known ones:  1. **Rheumatoid Arthritis (RA)**: This affects the joints, causing inflammation, pain, and potential joint damage.  2. **Lupus (Systemic Lupus Erythematosus, SLE)**: A systemic condition that can affect the skin, joints, kidneys, brain, and other organs.  3. **Type 1 Diabetes Mellitus**: This results from the immune system attacking the insulin-producing cells in the pancreas.  4. **Multiple Sclerosis (MS)**: This disease affects the brain and spinal cord, leading to a range of neurological symptoms.  5. **Psoriasis and Psoriatic Arthritis**: These conditions lead to skin issues and, in the case of psoriatic arthritis, joint problems.  6. **Inflammatory Bowel Disease (IBD)**: This includes Crohn's disease and ulcerative colitis, which primarily affect the gastrointestinal tract.  7. **Hashimoto’s Thyroiditis**: This results in hypothyroidism due to the immune system attacking the thyroid gland.  8. **Graves' Disease**: Unlike Hashimoto's, this leads to hyperthyroidism, where the thyroid is overactive.  9. **Myasthenia Gravis**: This affects the voluntary muscles, especially those controlling the eyes, mouth, throat, and limbs.  10. **Scleroderma**: This leads to hardening and tightening of the skin and connective tissues.  11. **Celiac Disease**: This disease triggers an immune response to gluten that damages the small intestine.  Each of these diseases can have significant impacts on the body and may require a variety of treatments to manage symptoms and prevent progression. |
| 1. What are autoantibodies? | Autoantibodies are a type of antibody produced by the immune system that mistakenly targets and reacts with a person's own tissues or organs. Normally, antibodies are part of the immune response to foreign pathogens, such as bacteria and viruses, helping to protect the body by identifying and neutralizing these invaders. However, autoantibodies can lead to autoimmune diseases, where they cause inflammation, damage, and dysfunction in various parts of the body depending on the specific tissues they target.  For example, in rheumatoid arthritis, autoantibodies attack the joints; in systemic lupus erythematosus, they can affect skin, joints, and organs like the kidneys; and in type 1 diabetes, they target insulin-producing cells in the pancreas. Testing for specific autoantibodies can help diagnose these conditions and monitor their progression. |
| 1. What are antinuclear antibodies? | Antinuclear antibodies (ANAs) are a type of autoantibody that target substances within the nucleus of cells. They play a significant role in autoimmune disorders. When the immune system malfunctions, it may produce ANAs that mistakenly target and react with a person's own tissues, potentially leading to inflammation and damage.  ANAs are commonly tested for in the diagnosis of various autoimmune diseases, such as systemic lupus erythematosus (SLE), Sjögren's syndrome, scleroderma, and mixed connective tissue disease, among others. A positive ANA test might suggest an autoimmune process is present, but it is not solely indicative of a specific disease; it must be interpreted in the context of the patient's symptoms and clinical findings. |
| **Topic: Clinical Feature** | |
| 1. What clinical symptoms should be considered to exclude autoimmune diseases? | To properly exclude autoimmune diseases, clinicians consider a wide array of symptoms since these diseases can affect almost any part of the body. However, the absence of certain key symptoms might help to rule out autoimmune diseases. Here are some general guidelines:  1. **Absence of Autoantibodies**: Many autoimmune diseases are characterized by the presence of specific autoantibodies. Their absence, especially those that are highly specific (e.g., anti-nuclear antibodies in systemic lupus erythematosus), might suggest other diagnoses.  2. **Lack of Systemic Symptoms**: Autoimmune diseases often present with systemic symptoms such as fever, fatigue, and weight loss. The absence of these systemic effects might point away from an autoimmune cause.  3. **Organ-Specific Symptoms**: The presence of symptoms linked to specific organs can be indicative of autoimmune diseases. For instance, dry eyes and dry mouth might suggest Sjögren's syndrome, while their absence in a different clinical context might lean away from this diagnosis.  4. **Inflammatory Markers**: Normal levels of inflammatory markers such as C-reactive protein (CRP) and erythrocyte sedimentation rate (ESR) can sometimes suggest a non-autoimmune process, although these markers can be normal in some autoimmune diseases as well.  5. **Response to Immunosuppressive Therapy**: If symptoms improve with immunosuppressive therapy, it might indicate an autoimmune process. Conversely, a lack of response could point towards a different diagnosis, though this isn't always definitive.  6. **Chronicity and Pattern of Symptoms**: Autoimmune diseases typically have a chronic, relapsing-remitting course. A different pattern might suggest another etiology.  It's important to consider these symptoms in conjunction with detailed patient history and comprehensive diagnostic testing. No single symptom can definitively exclude autoimmune diseases, making it crucial to use a combination of clinical judgment and diagnostic results. |
| 1. What harm do autoimmune diseases cause? | Autoimmune diseases can cause a variety of harmful effects, primarily due to the immune system mistakenly attacking the body's own tissues, thinking they are foreign invaders. This can lead to inflammation, damage, and dysfunction in different parts of the body, depending on the specific disease. Here are some common impacts:  1. **Chronic Inflammation**: Most autoimmune diseases cause chronic inflammation, which can damage tissues, organs, and joints over time.    2. **Organ Damage**: Specific organs can be targeted, such as the thyroid in Graves' disease or the pancreas in type 1 diabetes, leading to organ dysfunction and failure.  3. **Joint and Muscle Pain**: Conditions like rheumatoid arthritis and lupus primarily affect joints, causing swelling, pain, and deformity which can limit mobility.  4. **Skin Problems**: Diseases like psoriasis and scleroderma can lead to severe skin rashes, scarring, and other skin changes.  5. **Nervous System Damage**: Conditions like multiple sclerosis and myasthenia gravis affect the nervous system, leading to muscle weakness, coordination problems, and sensory dysfunction.  6. **Autoimmune Reactions**: Can lead to systemic symptoms such as fatigue, fever, and malaise, which can significantly impact quality of life.  The treatment often involves managing symptoms and trying to modulate the immune response without compromising the body's ability to fight actual pathogens. Early diagnosis and intervention can be crucial in preventing severe damage and improving the quality of life for individuals with autoimmune diseases. |
| **Topic: Report Interpretation** | |
| 1. Male patient, 66 years old. Test results show anti-cyclic citrullinated peptide antibody (CCP) 186.6 RU/mL (reference range ≤5 RU/mL), anti-mutated citrullinated vimentin antibody (MCV) 911 U/mL (reference range <20 U/mL), and positive anti-keratin antibody (AKA) (reference range negative). What disease should be considered and which specialty should the patient consult? | The test results you've provided indicate significantly elevated levels of anti-cyclic citrullinated peptide antibody (CCP), anti-mutated citrullinated vimentin antibody (MCV), and a positive anti-keratin antibody (AKA). These findings are highly suggestive of rheumatoid arthritis (RA), an autoimmune disease characterized by chronic inflammation of the joints and other areas of the body.  Given these results:  1. **Disease to Consider**: Rheumatoid arthritis should be strongly considered. The presence of high levels of anti-CCP and anti-MCV antibodies is particularly significant, as these are specific markers that not only help in the diagnosis of RA but also may indicate a more severe form of the disease.  2. **Specialty Consultation**: The patient should consult a rheumatologist. Rheumatologists specialize in arthritis and other diseases of the joints, muscles, and bones. They can provide a comprehensive evaluation and discuss appropriate treatment options to manage symptoms and prevent joint damage.  It would be advisable to start with a rheumatology referral to ensure an accurate diagnosis and to initiate the most effective treatment strategy early, which is crucial in managing rheumatoid arthritis. |
| 1. Female patient, 45 years old. The test report shows positive antinuclear antibody test (fluorescence method), with a fine granular speckled nuclear pattern, titer at 1:1000 positive. Anti-U1RNP/Sm antibody negative, anti-Sm antibody negative, anti-Scl-70 antibody negative, anti-Pm-scl antibody negative, anti-Jo-1 antibody negative, anti-CENP-B antibody negative, anti-PCNA antibody negative, anti-dsDNA antibody (imprinting method) negative, anti-nucleosome antibody negative, anti-histone antibody negative, anti-ribosomal P protein antibody negative, anti-mitochondrial M2 antibody negative, positive anti-SSA antibody, positive anti-SSB antibody, positive anti-Ro-52 antibody, rheumatoid factor 5.6 IU/mL (reference range <20 IU/mL), anti-ds-DNA antibody (ELISA) 0.001 IU/mL (reference range <100 IU/mL). What disease should be considered and which specialty should the patient consult? | The test results for the 45-year-old female patient suggest a high likelihood of Sjögren's syndrome. This autoimmune disease typically presents with symptoms including dry mouth, dry eyes, and other systemic effects. The positive antinuclear antibody (ANA) test with a speckled pattern and high titer (1:1000), along with positive anti-SSA (Ro), anti-SSB (La), and anti-Ro-52 antibodies, are supportive of this diagnosis. These antibodies are commonly associated with Sjögren's syndrome.  Additionally, the fact that the other specific antibodies (such as anti-Sm, anti-Scl-70, anti-dsDNA) are negative helps rule out other connective tissue diseases like systemic lupus erythematosus (SLE) or systemic sclerosis.  Given these findings, the patient should consult a rheumatologist. A rheumatologist specializes in diagnosing and managing autoimmune conditions and will be able to provide a comprehensive evaluation and appropriate management plan for Sjögren's syndrome or any other potential autoimmune disorders suggested by her symptoms and test results. |
| 1. Female patient, 49 years old. The test report shows positive antinuclear antibody test (fluorescence method), with a mitotic pattern, titer at 1:1000 positive. Anti-U1RNP/Sm antibody negative, anti-Sm antibody negative, anti-Scl-70 antibody negative, anti-Pm-scl antibody negative, anti-Jo-1 antibody negative, positive anti-CENP-B antibody, negative anti-PCNA antibody, negative anti-dsDNA antibody (imprinting method), negative anti-nucleosome antibody, negative anti-histone antibody, negative anti-ribosomal P protein antibody, weakly positive anti-mitochondrial M2 antibody, negative anti-SSA antibody, negative anti-SSB antibody, negative anti-Ro-52 antibody, rheumatoid factor 4.4 IU/mL (reference range <20 IU/mL), anti-ds-DNA antibody (ELISA) 12.21 IU/mL (reference range <100 IU/mL). What disease should be considered and which specialty should the patient consult? | Given the results of the test report for the 49-year-old female patient, several points stand out that help narrow down the differential diagnosis:  1. **Positive Antinuclear Antibody (ANA) Test with a Mitotic Pattern, High Titer (1:1000)**: This finding suggests an autoimmune disorder. The mitotic pattern is less common and can sometimes be associated with certain specific conditions.  2. **Positive Anti-CENP-B Antibody**: This is particularly associated with CREST syndrome, a variant of systemic sclerosis (scleroderma). CREST stands for Calcinosis, Raynaud's phenomenon, Esophageal dysfunction, Sclerodactyly, and Telangiectasia.  3. **Negative for Other Specific Autoimmune Markers**: The negativity for anti-Sm, anti-Scl-70, anti-Jo-1, and other specific antibodies, along with the specific positive and negative findings, can help exclude other connective tissue diseases like lupus, dermatomyositis, and mixed connective tissue disease.  4. **Weakly Positive Anti-mitochondrial M2 Antibody**: Although weakly positive, this could suggest some overlap with autoimmune liver conditions like primary biliary cholangitis; however, this would typically require additional liver function tests and clinical correlation for further consideration.  5. **Normal Rheumatoid Factor and Anti-ds-DNA Antibodies**: These findings are helpful to exclude rheumatoid arthritis and systemic lupus erythematosus (SLE), respectively, given their normal ranges.  Based on the lab findings, the most prominent consideration would be a form of systemic sclerosis, possibly the CREST variant, given the positive anti-CENP-B antibody. Therefore, the patient should consult a **rheumatologist**, who specializes in autoimmune and connective tissue diseases. The rheumatologist can provide a comprehensive evaluation and management plan, which may include further diagnostic testing and assessment of organ involvement, which is crucial in systemic sclerosis and related disorders. |
| 1. Female patient, 82 years old. The test report shows negative anti-Ro-52 antibody, negative anti-EJ antibody, negative anti-PL-12 antibody, positive anti-PL-7 antibody, negative anti-SRP antibody, negative anti-Jo-1 antibody, negative anti-PM-SCl75 antibody, negative anti-PM-SCl100 antibody, negative anti-Ku antibody, negative anti-Mi-2 antibody. What disease should be considered and which specialty should the patient consult? | The test results for the 82-year-old female patient indicate the presence of positive anti-PL-7 antibodies while other tested antibodies, including anti-Ro-52, anti-EJ, anti-PL-12, anti-SRP, anti-Jo-1, anti-PM-Scl75, anti-PM-Scl100, anti-Ku, and anti-Mi-2, are negative. The presence of anti-PL-7 antibodies is particularly associated with antisynthetase syndrome, a type of autoimmune myositis.  Antisynthetase syndrome is characterized by muscle weakness, interstitial lung disease, arthritis, fever, and Raynaud's phenomenon. Given the positive anti-PL-7 result, this syndrome should be considered as a possible diagnosis for the patient.  For management and further evaluation, the patient should consult with a rheumatologist, as this specialty is well-versed in autoimmune conditions and systemic diseases like antisynthetase syndrome. Additionally, due to the common involvement of the lungs in this syndrome, coordination with a pulmonologist may also be necessary, especially if there are respiratory symptoms or concerns. |
| 1. Female patient, 47 years old. The test report shows positive antinuclear antibody test (fluorescence method), with a nuclear envelope pattern, titer at 1:1000 positive. What disease should be considered and which specialty should the patient consult? | In a 47-year-old female patient with a positive antinuclear antibody (ANA) test showing a nuclear envelope pattern and a high titer of 1:1000, several conditions can be considered. The ANA test, particularly with the nuclear envelope pattern, is often associated with autoimmune disorders. Specific diseases that may present with this pattern include:  1. **Systemic Lupus Erythematosus (SLE)**: This is a common autoimmune disease associated with ANA positivity, though the nuclear envelope pattern is less typical for SLE.  2. **Primary Biliary Cholangitis (PBC)**: This condition, primarily affecting the liver, can show a nuclear envelope pattern in ANA testing.  3. **Autoimmune Hepatitis**: Similar to PBC, this liver disease can also be associated with ANA positivity.  4. **Mixed Connective Tissue Disease (MCTD)** and other systemic rheumatic diseases: These might also present with various patterns on ANA testing.  Given the nuclear envelope staining pattern and the high titer, it's crucial to look at the patient's clinical presentation, including symptoms and other laboratory findings, to guide further investigation.  The patient should consult with a **rheumatologist**, as this specialty primarily deals with autoimmune diseases and will be able to conduct a thorough assessment, which may include additional autoimmune serology tests, clinical evaluation, and possibly imaging studies to confirm the diagnosis and commence appropriate treatment. |
| 1. Female patient, 49 years old. The test report shows positive antinuclear antibody test (fluorescence method), with a fine granular speckled nuclear pattern, titer at 1:3200 positive; spindle pattern, 1:320 positive; nuclear envelope pattern, 1:320 positive. Anti-U1RNP/Sm antibody negative, anti-Sm antibody negative, anti-Scl-70 antibody negative, anti-Pm-scl antibody negative, anti-Jo-1 antibody negative, positive anti-CENP-B antibody, negative anti-PCNA antibody, negative anti-dsDNA antibody (imprinting method), negative anti-nucleosome antibody, negative anti-histone antibody, negative anti-ribosomal P protein antibody, negative anti-mitochondrial M2 antibody, negative anti-mitochondrial M4 antibody, positive anti-mitochondrial M2 antibody, positive anti-SSA antibody, negative anti-SSB antibody, positive anti-Ro-52 antibody, rheumatoid factor 2.1 IU/mL (reference range <20 IU/mL), anti-ds-DNA antibody (ELISA) 5.323 IU/mL (reference range <100 IU/mL). What disease should be considered and which specialty should the patient consult? | The test results you've described indicate a complex autoimmune profile that requires careful consideration. Here’s a breakdown of the key findings and their implications:  1. **Positive Antinuclear Antibody (ANA) Test**: This is a screening test used to help diagnose autoimmune disorders. A high titer of 1:3200 with a fine granular speckled nuclear pattern often suggests a systemic autoimmune rheumatic disease.  2. **Specific Antibody Patterns**:  - **Positive Anti-CENP-B Antibody**: Typically associated with systemic sclerosis (scleroderma), particularly the limited cutaneous form.  - **Positive Anti-Mitochondrial M2 Antibody**: Commonly linked to primary biliary cholangitis, but this is less likely without other supporting clinical symptoms or liver enzyme abnormalities.  - **Positive Anti-SSA and Anti-Ro-52 Antibodies**: These antibodies are often seen in Sjögren’s syndrome and systemic lupus erythematosus (SLE), and less commonly in systemic sclerosis and myositis.  Given these antibodies and patterns, and the absence of other specific antibodies like anti-Sm, anti-dsDNA (which are more specific for Sjögren’s syndrome or SLE), the patient's results suggest a possible overlap of connective tissue diseases. The presence of Anti-CENP-B is particularly suggestive of a scleroderma spectrum disorder, but the presence of anti-SSA and anti-Ro-52 broadens the differential diagnosis to include other rheumatic diseases.  ### Suggested Consultation:  The patient should consult a **rheumatologist**. A rheumatologist specializes in autoimmune and rheumatic diseases and will be able to conduct further assessments, possibly including clinical evaluation, imaging studies, and additional lab tests to confirm the diagnosis and tailor the treatment plan. This specialist can also determine if there is an overlap of autoimmune conditions, which seems possible in this case, and manage treatment accordingly. |
| 1. Male patient, 36 years old. Test results show anti-cyclic citrullinated peptide antibody (CCP) 50.6 RU/mL (reference range ≤5 RU/mL), anti-mutated citrullinated vimentin antibody (MCV) 10.2 U/mL (reference range <20 U/mL), negative anti-keratin antibody (AKA) (reference range negative). What disease should be considered and which specialty should the patient consult? | The test results indicate a significant elevation in anti-cyclic citrullinated peptide antibody (CCP) levels with a reading of 50.6 RU/mL, which is much higher than the reference range of ≤5 RU/mL. Elevated levels of CCP are strongly associated with rheumatoid arthritis (RA), a chronic inflammatory disorder affecting the joints. The anti-mutated citrullinated vimentin antibody (MCV) level is within normal limits, and the anti-keratin antibody (AKA) is negative, which further supports the consideration of RA, as CCP is a more specific marker for this disease.  Given these results and the clinical context, the patient should be referred to a rheumatologist. Rheumatologists specialize in the diagnosis and management of rheumatic diseases such as rheumatoid arthritis and can provide a comprehensive evaluation and appropriate management plan based on the full spectrum of symptoms and test results. |
| 1. Female patient, 44 years old. Test results show anti-cyclic citrullinated peptide antibody (CCP) 2.3 RU/mL (reference range ≤5 RU/mL), anti-mutated citrullinated vimentin antibody (MCV) 195.2 U/mL (reference range <20 U/mL), negative anti-keratin antibody (AKA) (reference range negative). What disease should be considered and which specialty should the patient consult? | Based on the laboratory test results you provided, here's a detailed analysis:  1. **Anti-Cyclic Citrullinated Peptide (CCP) Antibody**: The level of 2.3 RU/mL is within the normal reference range (≤5 RU/mL). Anti-CCP antibodies are specific markers often associated with rheumatoid arthritis (RA). The normal level in this test usually suggests a lower likelihood of RA, but it does not completely rule it out.  2. **Anti-Mutated Citrullinated Vimentin (MCV) Antibody**: The significantly elevated level of 195.2 U/mL (with a reference range <20 U/mL) is noteworthy. High levels of anti-MCV antibodies are also associated with rheumatoid arthritis, and they can be indicative of disease activity and severity.  3. **Anti-Keratin Antibody (AKA)**: This test result is negative, which is typical for the general population. AKA is less commonly used in clinical practice today and has lower specificity for rheumatoid arthritis compared to anti-CCP and anti-MCV antibodies.  Given the high anti-MCV level despite a normal anti-CCP level, rheumatoid arthritis should still be considered as a potential diagnosis. The variability in antibody presentations can sometimes occur based on individual differences and the specific characteristics of the disease in each patient.  **Recommendation for Specialty Consultation**:  The patient should consult a rheumatologist. Rheumatology is the specialty dealing with arthritis and other rheumatic conditions. A rheumatologist will be able to further evaluate these findings in the context of the patient's clinical symptoms, perform a physical examination, and possibly order additional tests (like imaging studies or joint fluid analysis) to clarify the diagnosis and discuss appropriate treatment options. |
| 1. Female patient, 25 years old. The test report shows positive antinuclear antibody test (fluorescence method), with a homogeneous pattern, titer at 1:3200 positive. What disease should be considered and which specialty should the patient consult? | Based on the positive antinuclear antibody (ANA) test result with a homogeneous pattern and a high titer of 1:3200, we should consider the possibility of systemic lupus erythematosus (SLE). A homogeneous pattern on ANA testing is commonly associated with SLE, among other autoimmune diseases.  Systemic lupus erythematosus is a chronic autoimmune disease that can affect various organs and tissues in the body, leading to inflammation and damage. Common symptoms include joint pain, fatigue, skin rashes, fever, and organ involvement such as in the kidneys, heart, lungs, or central nervous system.  Given the complexity and potential multi-organ involvement of SLE, the patient should consult a rheumatologist for further evaluation, management, and ongoing care. Rheumatologists specialize in diagnosing and treating autoimmune and inflammatory conditions like SLE, providing comprehensive care to address the various aspects of the disease. |
| 1. Male patient, 61 years old. The test report shows positive perinuclear anti-neutrophil cytoplasmic antibody (pANCA), negative cytoplasmic anti-neutrophil cytoplasmic antibody (cANCA), anti-myeloperoxidase antibody (Anti-MPO) 10.5 RU/mL (reference range <20 U/mL), anti-proteinase 3 antibody 0.001 RU/mL (reference range <20 U/mL). What disease should be considered and which specialty should the patient consult? | Based on the test results showing a positive perinuclear anti-neutrophil cytoplasmic antibody (pANCA) and negative cytoplasmic anti-neutrophil cytoplasmic antibody (cANCA), along with normal levels of anti-myeloperoxidase antibody (Anti-MPO) and anti-proteinase 3 antibody, the patient may be indicative of having microscopic polyangiitis (MPA).  MPA is a systemic necrotizing vasculitis that primarily affects small vessels, typically involving the kidneys, lungs, skin, joints, and nerves. It often presents with features such as renal impairment, pulmonary involvement, and constitutional symptoms.  Given the complexity and multi-system involvement of MPA, the patient should consult with a rheumatologist for further evaluation, management, and treatment. A rheumatologist is specialized in diagnosing and treating autoimmune and inflammatory conditions like vasculitis. They will assess the patient comprehensively, conduct further investigations if needed, and develop a suitable treatment plan, which may involve immunosuppressive therapy to control inflammation and prevent organ damage. |
| 1. Male patient, 67 years old. The test report shows negative perinuclear anti-neutrophil cytoplasmic antibody (pANCA), positive cytoplasmic anti-neutrophil cytoplasmic antibody (cANCA), anti-myeloperoxidase antibody (Anti-MPO) 0.001 RU/mL (reference range <20 U/mL), anti-proteinase 3 antibody 96.5 RU/mL (reference range <20 U/mL). What disease should be considered and which specialty should the patient consult? | Based on the test results provided, the patient is showing positive cytoplasmic anti-neutrophil cytoplasmic antibody (cANCA) and elevated levels of anti-proteinase 3 antibody, while perinuclear anti-neutrophil cytoplasmic antibody (pANCA) is negative. These findings are indicative of a systemic autoimmune condition known as Granulomatosis with Polyangiitis (GPA), previously called Wegener's granulomatosis.  Granulomatosis with Polyangiitis (GPA) is a rare autoimmune disorder characterized by inflammation of blood vessels (vasculitis), leading to damage in various organs, particularly the lungs, kidneys, and upper respiratory tract.  Specialists who commonly manage patients with GPA include rheumatologists, nephrologists, and pulmonologists. Given the systemic nature of GPA and potential involvement of multiple organs, it would be advisable for the patient to consult with a rheumatologist, who can coordinate care and determine the most appropriate treatment plan based on the patient's individual symptoms and disease severity. |
| 1. Female patient, 54 years old, the test report shows negative mitochondrial antibody M2, negative anti-pyruvate dehydrogenase complex antibody, negative anti-acidic phosphoprotein 100 antibody, negative anti-promyelocytic leukemia antibody, negative anti-nuclear envelope glycoprotein antibody, negative anti-Ro-52 antibody, negative anti-liver kidney microsome-1 antibody, negative anti-soluble liver antigen/liver pancreas antigen, positive smooth muscle antibody. What disease should be considered? | Based on the test results provided, the patient is negative for a range of antibodies typically associated with autoimmune diseases such as primary biliary cirrhosis (PBC), autoimmune hepatitis, and primary sclerosing cholangitis. However, the positive result for smooth muscle antibody (SMA) is significant.  Positive SMA is commonly associated with autoimmune hepatitis (AIH), particularly type 1 autoimmune hepatitis. Therefore, considering the patient's age and the pattern of antibody positivity, autoimmune hepatitis would be a primary consideration. However, it's important to note that the diagnosis should be made in conjunction with other clinical findings and possibly additional tests, as autoimmune hepatitis is a complex disease with varying presentations. A liver biopsy might also be warranted to confirm the diagnosis and assess the degree of liver damage. |
| 1. Female patient, test report shows positive antinuclear antibody test (fluorescence method), with a fine granular speckled nuclear pattern, titer at 1:3200 positive; cytoplasmic granular pattern, 1:320 positive. Positive anti-U1RNP/Sm antibody, positive anti-Sm antibody, negative anti-Scl-70 antibody, negative anti-Pm-scl antibody, negative anti-Jo-1 antibody, negative anti-CENP-B antibody, negative anti-PCNA antibody, positive anti-dsDNA antibody (imprinting method), positive anti-nucleosome antibody, positive anti-histone antibody, negative anti-ribosomal P protein antibody, positive anti-SSA antibody, negative anti-SSB antibody, positive anti-Ro-52 antibody, rheumatoid factor 5.6 IU/mL (reference range <20 IU/mL), anti-ds-DNA antibody (ELISA) 327.323 IU/mL (reference range <100 IU/mL). What disease should be considered and which specialty should the patient consult? | Based on the test results provided, the patient presents with a complex autoimmune profile indicative of systemic lupus erythematosus (SLE), a chronic autoimmune disease that can affect multiple organs and tissues. The positive antinuclear antibody (ANA) test with a fine granular speckled nuclear pattern and high titer of 1:3200, along with the presence of specific autoantibodies such as anti-dsDNA, anti-nucleosome, anti-histone, anti-Sm, anti-U1RNP/Sm, anti-SSA, and anti-Ro-52 antibodies, strongly suggest SLE.  Additionally, the patient's symptoms and clinical history would be crucial for confirming the diagnosis and determining the severity and extent of organ involvement. These may include symptoms such as joint pain, skin rashes, fatigue, fever, and involvement of organs like the kidneys, heart, lungs, and central nervous system.  Given the complexity and multisystem involvement of SLE, the patient should be referred to a rheumatologist for further evaluation, management, and ongoing care. Rheumatologists specialize in the diagnosis and treatment of autoimmune and inflammatory diseases, including SLE. They are equipped to coordinate care with other specialists as needed to address the various manifestations of the disease. Early detection and appropriate management are key in improving outcomes and quality of life for patients with SLE. |
| **Topic: Diagnose** | |
| 1. What laboratory tests are needed to help doctors diagnose if a patient has an autoimmune disease? | Diagnosing autoimmune diseases often requires a combination of clinical evaluation, medical history assessment, physical examination, and laboratory tests. Here are some common laboratory tests that can aid in diagnosing autoimmune diseases:  1. **Autoantibody tests**: These tests detect antibodies that target the body's own tissues. Examples include:  - Antinuclear antibodies (ANA): Commonly associated with systemic lupus erythematosus (SLE) and other connective tissue diseases.  - Anti-double-stranded DNA (anti-dsDNA) antibodies: Seen in SLE.  - Anti-cyclic citrullinated peptide (anti-CCP) antibodies: Associated with rheumatoid arthritis.  - Anti-thyroid antibodies (e.g., anti-thyroid peroxidase, anti-thyroglobulin): Indicative of autoimmune thyroid diseases such as Hashimoto's thyroiditis and Graves' disease.  - Anti-neutrophil cytoplasmic antibodies (ANCA): Linked to vasculitis and other autoimmune conditions.  2. **Inflammatory markers**: Elevated levels of certain markers indicate ongoing inflammation, which is common in autoimmune diseases. Examples include:  - C-reactive protein (CRP)  - Erythrocyte sedimentation rate (ESR)  - Serum complement levels (C3 and C4): Reduced levels may suggest complement consumption in diseases like lupus.  3. **Rheumatoid factor (RF)**: This antibody is often present in rheumatoid arthritis but can also be found in other autoimmune conditions.  4. **Complete blood count (CBC)**: Abnormalities such as anemia, leukopenia, or thrombocytopenia may occur in autoimmune diseases.  5. **Organ-specific tests**: Depending on the suspected autoimmune disease, additional tests may be necessary. For example:  - Thyroid function tests (TSH, T4, T3) for thyroid disorders.  - Liver function tests (ALT, AST, bilirubin) for autoimmune hepatitis.  - Anti-glomerular basement membrane (anti-GBM) antibodies for Goodpasture's syndrome.  6. **Immunoglobulin levels**: Measurement of immunoglobulin levels (IgG, IgM, IgA) can provide insights into the immune system's activity.  7. **Genetic testing**: In some cases, genetic testing may be warranted, especially for diseases with a strong genetic component like certain types of arthritis.  It's important to note that no single test can definitively diagnose all autoimmune diseases, and results should be interpreted in the context of the patient's clinical presentation and medical history. Additionally, not all autoimmune diseases have specific diagnostic markers, so a comprehensive evaluation by a healthcare provider is essential for accurate diagnosis and management. |
| 1. Does a positive result in autoantibody testing necessarily mean that the patient has an autoimmune disease? | Not necessarily. A positive result in autoantibody testing indicates the presence of autoantibodies in the patient's bloodstream, which are antibodies that target the body's own tissues or cells. While the presence of autoantibodies is often associated with autoimmune diseases, it's important to remember that autoantibodies can also be found in individuals without autoimmune diseases.  There are several reasons for this:  1. **False positives**: Autoantibody tests may sometimes produce false positive results due to technical issues or cross-reactivity with other antibodies.  2. **Transient autoantibodies**: Some individuals may have transient autoantibodies that don't necessarily indicate an ongoing autoimmune disease. These may arise due to infections, medications, or other factors.  3. **Asymptomatic carriers**: Some people may have autoantibodies but never develop symptoms of an autoimmune disease. This could be due to genetic factors or environmental triggers that haven't yet manifested into a clinical condition.  4. **Overlap syndromes**: In some cases, autoantibodies associated with one autoimmune disease may be present in individuals with a different autoimmune condition or even in healthy individuals.  Therefore, while a positive result in autoantibody testing can provide valuable diagnostic information and may suggest the presence of an autoimmune disease, it's not definitive proof on its own. Further clinical evaluation, including consideration of symptoms, medical history, and other diagnostic tests, is usually necessary to confirm the presence of an autoimmune condition. |
| 1. Does a positive result in antinuclear antibody testing necessarily mean that the patient has an autoimmune disease? | Not necessarily. While a positive result in antinuclear antibody (ANA) testing can indicate the presence of autoantibodies, which are antibodies that mistakenly target the body's own tissues, it doesn't always mean the patient has an autoimmune disease. ANA testing is a screening test, and a positive result could also be seen in various other conditions, including infections, chronic inflammatory diseases, and sometimes even in healthy individuals.  The interpretation of ANA results requires clinical correlation with the patient's symptoms, medical history, and other laboratory tests. Further diagnostic tests and evaluation by a healthcare provider are often necessary to determine if an autoimmune disease is present and, if so, which specific condition it may be. |
| 1. Is gout an autoimmune disease? | No, gout is not an autoimmune disease. It is a form of inflammatory arthritis that develops when uric acid crystals accumulate in the joints, leading to pain, swelling, and inflammation. Autoimmune diseases occur when the immune system mistakenly attacks the body's own tissues, whereas gout is primarily driven by metabolic factors related to uric acid levels in the blood. |
| 1. I am a patient with Sjögren's syndrome, why do I need an annual chest CT scan? | As a patient with Sjögren's syndrome, you may be recommended to undergo an annual chest CT scan for several reasons:  1. **Interstitial Lung Disease (ILD) Monitoring:** Sjögren's syndrome can involve various organs, including the lungs. Interstitial lung disease is a known complication of Sjögren's syndrome, characterized by inflammation and scarring of the lung tissue. Annual chest CT scans help monitor the progression of ILD and detect any changes early on.  2. **Pulmonary Manifestations:** Sjögren's syndrome can also lead to other pulmonary manifestations such as bronchiectasis, pulmonary nodules, or lymphadenopathy. Regular chest CT scans can help identify these conditions and monitor their progression over time.  3. **Detection of Complications:** Sjögren's syndrome increases the risk of developing certain complications such as pulmonary infections or lymphoma. Chest CT scans can aid in the early detection of these complications, allowing for prompt intervention and management.  4. **Assessment of Treatment Response:** If you are undergoing treatment for Sjögren's syndrome-related lung complications, annual chest CT scans can help assess the effectiveness of the treatment and make any necessary adjustments.  5. **Baseline Evaluation:** An initial chest CT scan can serve as a baseline for comparison in subsequent scans, providing valuable information about any changes or developments in your lung health over time.  It's important to discuss the specific reasons for undergoing an annual chest CT scan with your healthcare provider, as recommendations may vary based on individual factors such as disease severity, symptoms, and overall health status. |
| 1. Positive antinuclear antibodies, does a higher titer indicate more severe disease? | In general, the level or titer of antinuclear antibodies (ANA) in the blood does not necessarily correlate with the severity of disease. ANA are antibodies that mistakenly target the body's own cells' nuclei, and they are often present in autoimmune diseases such as systemic lupus erythematosus (SLE) and rheumatoid arthritis (RA).  While a higher ANA titer can sometimes indicate a greater likelihood of autoimmune disease, it's important to note that many healthy individuals can also have low levels of ANA. Additionally, some autoimmune diseases may have fluctuating ANA titers over time, so a single measurement may not provide a complete picture of disease severity.  Other factors, such as clinical symptoms, other laboratory tests, and imaging studies, are typically considered alongside ANA titers to help diagnose and monitor autoimmune diseases. Therefore, while ANA testing is an important tool in the evaluation of autoimmune diseases, interpreting the results requires careful consideration of the entire clinical context. |
| **Topic: Prevention and Treatment** | |
| 1. What are the high-risk factors for autoimmune diseases? | Autoimmune diseases are conditions where the body's immune system mistakenly attacks its own tissues. While the exact cause of autoimmune diseases is not fully understood, there are several factors that are known to increase the risk of developing these conditions:  1. **Genetics**: Family history plays a significant role in autoimmune diseases. If you have a close relative with an autoimmune disease, you are at a higher risk of developing one yourself.  2. **Gender**: Many autoimmune diseases, such as lupus, rheumatoid arthritis, and multiple sclerosis, are more common in women than in men. The reasons for this gender difference are not entirely clear but may involve hormonal factors.  3. **Age**: Some autoimmune diseases, like rheumatoid arthritis and multiple sclerosis, often develop during the childbearing years (ages 15 to 44). Others, such as lupus and type 1 diabetes, can occur at any age but are more common in young adults.  4. **Environmental Factors**: Certain environmental factors, such as infections, exposure to certain chemicals or pollutants, and even diet, may trigger autoimmune responses in genetically susceptible individuals.  5. **Hormonal Factors**: Hormonal changes, such as those that occur during puberty, pregnancy, and menopause, can affect the immune system and may increase the risk of developing autoimmune diseases.  6. **Chronic Stress**: Prolonged stress can weaken the immune system and may contribute to the development or exacerbation of autoimmune diseases.  7. **Smoking**: Smoking has been linked to an increased risk of several autoimmune diseases, including rheumatoid arthritis and lupus.  8. **Obesity**: Obesity is associated with inflammation and immune system dysregulation, which may increase the risk of developing autoimmune diseases.  9. **Previous Infections**: Some autoimmune diseases may be triggered by previous infections, as the immune system may become confused and start attacking the body's own tissues after fighting off an infection.  It's important to note that having one or more of these risk factors does not necessarily mean you will develop an autoimmune disease, but it does increase the likelihood. If you have concerns about your risk factors or symptoms suggestive of an autoimmune condition, it's essential to consult with a healthcare professional for proper evaluation and management. |
| 1. Should autoantibodies be tested during routine physical examinations? | Autoantibodies are antibodies produced by the immune system that target the body's own tissues or organs. Testing for autoantibodies is not typically included in routine physical examinations unless there are specific clinical indications or risk factors present. However, there are certain conditions where screening for autoantibodies may be warranted, such as in individuals with a family history of autoimmune diseases, symptoms suggestive of autoimmune disorders, or certain demographic groups known to have a higher prevalence of autoimmune conditions.  The decision to test for autoantibodies should be made on a case-by-case basis by a healthcare provider, taking into consideration the individual's medical history, symptoms, and risk factors. In some cases, screening for autoantibodies may be part of a broader diagnostic workup to investigate suspected autoimmune diseases. It's important to note that the presence of autoantibodies does not necessarily indicate the presence of an autoimmune disease, as these antibodies can sometimes be found in healthy individuals or in association with other conditions.  Overall, while autoantibody testing may not be routine in every physical examination, it can be a valuable tool in diagnosing and managing autoimmune diseases when clinically indicated. |
| 1. Should antinuclear antibodies be tested during routine physical examinations? | Antinuclear antibodies (ANAs) are autoantibodies that can target the body's own tissues, particularly the nucleus of cells. Testing for ANAs is typically done to aid in the diagnosis of autoimmune diseases, such as systemic lupus erythematosus (SLE), rheumatoid arthritis (RA), and Sjögren's syndrome, among others.  While ANA testing can be valuable in diagnosing autoimmune conditions, it's not typically included in routine physical examinations for everyone. However, there are certain situations where ANA testing may be considered:  1. **Symptoms suggestive of autoimmune disease**: If a patient presents with symptoms such as joint pain, fatigue, skin rashes, or other signs indicative of autoimmune disorders, ANA testing may be appropriate.  2. **Family history of autoimmune disease**: Individuals with a family history of autoimmune diseases may be at higher risk themselves, warranting ANA testing if symptoms arise.  3. **Monitoring**: For patients already diagnosed with autoimmune diseases, periodic monitoring of ANA levels may be part of their ongoing management.  4. **Certain medications**: Some medications can induce autoimmune-like symptoms or even trigger autoimmune diseases. ANA testing may be considered in such cases.  However, it's important to note that ANA testing alone is not diagnostic of a specific autoimmune disease. Further evaluation, including clinical examination, other laboratory tests, and sometimes biopsy, may be necessary to confirm a diagnosis.  In summary, while ANA testing is valuable in certain clinical contexts, it's not typically included in routine physical examinations for everyone. Its use should be guided by clinical suspicion and the presence of symptoms or risk factors suggestive of autoimmune disease. |
| 1. Do autoimmune diseases have a hereditary component? My mother has Sjögren's syndrome, how can I prevent myself from getting sick? | Yes, autoimmune diseases can have a hereditary component. While the exact cause of autoimmune diseases is still not fully understood, genetics do play a role in predisposing individuals to develop these conditions. If your mother has Sjögren's syndrome, there may be an increased risk for you to develop it as well.  However, it's important to note that genetics is just one factor, and environmental factors also play a significant role in the development of autoimmune diseases. While you can't change your genetic predisposition, there are steps you can take to potentially reduce your risk or manage the condition if you do develop it:  1. **Regular Health Check-ups:** Stay vigilant with regular health check-ups and screenings. Early detection and treatment can help manage the symptoms and slow down the progression of autoimmune diseases.  2. **Healthy Lifestyle:** Maintain a healthy lifestyle by eating a balanced diet, getting regular exercise, managing stress levels, and avoiding smoking and excessive alcohol consumption. A healthy lifestyle can help support your immune system and reduce inflammation in the body.  3. **Sun Protection:** For certain autoimmune diseases like lupus, exposure to sunlight can trigger flare-ups. Therefore, practicing sun protection measures, such as wearing sunscreen, protective clothing, and seeking shade, can be beneficial.  4. **Stay Informed:** Stay informed about your family medical history and discuss it with your healthcare provider. They can provide personalized recommendations and guidance based on your individual risk factors.  5. **Consult a Specialist:** If you have concerns about your risk for developing autoimmune diseases, consider consulting a rheumatologist or immunologist. They can assess your risk factors, provide recommendations for prevention, and monitor your health closely.  While there's no guaranteed way to prevent autoimmune diseases, adopting a proactive approach to your health and staying informed can help you manage your risk and maintain overall well-being. |
| 1. I have systemic lupus erythematosus, what should I pay attention to in daily life? | As a chief physician specializing in laboratory medicine, I would advise you to pay close attention to several aspects of your daily life if you have systemic lupus erythematosus (SLE). Here are some key points to consider:  1. **Medication Adherence**: It's crucial to take your prescribed medications regularly and as directed by your healthcare provider. This may include immunosuppressants, anti-inflammatory drugs, and medications to manage specific symptoms.  2. **Sun Protection**: People with SLE are often photosensitive, meaning they are more sensitive to sunlight. Protect yourself by wearing sunscreen with a high SPF, protective clothing, and hats when outdoors. Try to avoid direct sunlight, especially during peak hours.  3. **Stress Management**: Stress can exacerbate lupus symptoms. Incorporate stress-reduction techniques into your daily routine, such as meditation, yoga, deep breathing exercises, or hobbies that you enjoy.  4. **Regular Exercise**: Physical activity can help improve overall health and well-being. However, it's essential to find a balance and avoid overexertion, as fatigue and joint pain are common symptoms of SLE. Consult with your healthcare provider to develop a safe and suitable exercise plan.  5. **Healthy Diet**: Maintain a balanced and nutritious diet to support your immune system and overall health. Some people with SLE may benefit from dietary adjustments, such as reducing inflammation-promoting foods and incorporating more anti-inflammatory foods like fruits, vegetables, whole grains, and omega-3 fatty acids.  6. **Regular Medical Monitoring**: Keep up with regular check-ups and follow-up appointments with your healthcare provider. Monitoring your condition allows for early detection of any changes or complications and enables adjustments to your treatment plan as needed.  7. **Avoiding Illness**: People with SLE may have a weakened immune system due to the disease itself or immunosuppressive medications. Take precautions to minimize your risk of infections, such as practicing good hand hygiene, avoiding close contact with sick individuals, and getting recommended vaccinations.  8. **Emotional Support**: Living with a chronic illness like SLE can be challenging emotionally. Seek support from friends, family, support groups, or a mental health professional if needed. It's essential to address any emotional or psychological concerns and maintain a positive outlook.  Remember that managing SLE involves a personalized approach tailored to your specific needs and circumstances. Be proactive in communicating with your healthcare team and advocating for your well-being. |
| 1. I have systemic lupus erythematosus, and my condition is stable under medication. What indicators should I regularly check? | As a chief physician in the laboratory department, I would recommend monitoring several indicators regularly for someone with systemic lupus erythematosus (SLE) to ensure their condition remains stable. Here are some key indicators to keep an eye on:  1. **Complete Blood Count (CBC):** This test helps monitor levels of red blood cells, white blood cells, and platelets. Changes in these levels can indicate inflammation, infection, or medication side effects.  2. **Kidney Function Tests:** Tests such as serum creatinine, blood urea nitrogen (BUN), and urine protein can help assess kidney function. SLE can affect the kidneys, so it's important to monitor for any signs of renal involvement.  3. **C-reactive Protein (CRP):** Elevated levels of CRP can indicate inflammation in the body, which is common in SLE. Monitoring CRP levels can help track disease activity.  4. **Erythrocyte Sedimentation Rate (ESR):** Similar to CRP, ESR is a marker of inflammation. It can be useful in monitoring disease activity in SLE patients.  5. **Autoantibody Testing:** Testing for specific autoantibodies commonly associated with SLE, such as antinuclear antibodies (ANA), anti-double stranded DNA (anti-dsDNA) antibodies, and anti-Smith (anti-Sm) antibodies, can help in diagnosing and monitoring the disease.  6. **Lipid Profile:** SLE patients may be at increased risk of cardiovascular disease, so monitoring lipid levels (cholesterol and triglycerides) is important for assessing cardiovascular risk.  7. **Liver Function Tests:** SLE and some medications used to treat it can affect liver function, so regular monitoring of liver enzymes such as ALT, AST, and bilirubin is important.  8. **Electrolyte Levels:** SLE patients may experience electrolyte imbalances due to kidney involvement or medication side effects. Monitoring electrolyte levels such as sodium, potassium, and calcium is important for overall health.  9. **Bone Density Testing:** Long-term use of corticosteroids, which are often used to manage SLE, can increase the risk of osteoporosis. Bone density testing can help assess bone health and guide management strategies.  It's important to work closely with your healthcare provider to determine the frequency of monitoring for these indicators based on your individual health status and treatment plan. Regular monitoring can help detect any changes early and ensure timely intervention to manage your SLE effectively. |
| 1. I have systemic lupus erythematosus, and my condition is stable under medication. How often should I go to the hospital for a check-up? | For patients with systemic lupus erythematosus (SLE), the frequency of hospital visits for check-ups can vary depending on individual factors such as the severity of the disease, the effectiveness of the current treatment regimen, and any additional health conditions. However, as a general guideline, patients with stable SLE typically require routine follow-up visits every 3 to 6 months.  During these check-up appointments, your healthcare team will assess your overall health, monitor for any changes in your symptoms, evaluate the effectiveness of your current medications, and address any concerns or questions you may have. They may also perform laboratory tests to monitor disease activity and assess for any potential complications or side effects of treatment.  It's essential to maintain open communication with your healthcare provider and adhere to the recommended follow-up schedule to ensure optimal management of your condition and to prevent any potential flare-ups or complications. Additionally, if you experience any new symptoms or changes in your health between scheduled appointments, it's important to contact your healthcare provider promptly for further evaluation and management. |
| 1. What are the common medications for autoimmune diseases? | Common medications for autoimmune diseases vary depending on the specific condition and its severity. Here are some categories of medications commonly used:  1. **Nonsteroidal Anti-Inflammatory Drugs (NSAIDs)**: These are often used to alleviate pain and inflammation in autoimmune diseases such as rheumatoid arthritis and lupus.  2. **Corticosteroids**: Prednisone and similar medications are potent anti-inflammatory drugs used to suppress the immune system's response in conditions like rheumatoid arthritis, lupus, and multiple sclerosis during flare-ups.  3. **Disease-Modifying Antirheumatic Drugs (DMARDs)**: These drugs, such as methotrexate, hydroxychloroquine, and sulfasalazine, work to slow down the progression of autoimmune diseases like rheumatoid arthritis by suppressing the immune system.  4. **Biologic Response Modifiers (Biologics)**: These medications target specific parts of the immune system involved in autoimmune diseases. Examples include adalimumab, etanercept, infliximab, and rituximab. Biologics are commonly used in conditions like rheumatoid arthritis, psoriasis, and inflammatory bowel disease.  5. **Immunosuppressants**: Drugs like azathioprine, cyclosporine, and mycophenolate mofetil suppress the immune system's activity and are used to manage autoimmune diseases like lupus, rheumatoid arthritis, and organ transplant rejection.  6. **Janus Kinase (JAK) Inhibitors**: These newer medications, such as tofacitinib, inhibit Janus kinases involved in the immune response and are used to treat conditions like rheumatoid arthritis and psoriatic arthritis.  7. **Monoclonal Antibodies**: These drugs target specific cells or proteins involved in autoimmune diseases. For example, ustekinumab targets interleukin-12 and interleukin-23 and is used to treat conditions like psoriasis and psoriatic arthritis.  It's important to note that these medications can have side effects and may require careful monitoring by healthcare professionals. Treatment plans should be personalized based on factors such as the specific autoimmune disease, its severity, the patient's overall health, and any other medications they may be taking. |
| 1. What are the treatment principles for autoimmune diseases? | The treatment principles for autoimmune diseases aim to suppress the abnormal immune response while minimizing side effects and maintaining overall health. Here are some common strategies:  1. **Immunosuppression**: Medications like corticosteroids, immunosuppressants (such as methotrexate, azathioprine, mycophenolate), and biologic agents (like TNF inhibitors, interleukin inhibitors) are used to dampen the overactive immune response.  2. **Anti-inflammatory drugs**: Nonsteroidal anti-inflammatory drugs (NSAIDs) can help reduce inflammation and alleviate symptoms like pain and swelling.  3. **Disease-modifying antirheumatic drugs (DMARDs)**: These drugs, such as methotrexate, hydroxychloroquine, and sulfasalazine, target the underlying disease process and may prevent long-term damage to tissues and organs.  4. **Biologic therapies**: Biologic drugs, including tumor necrosis factor (TNF) inhibitors, interleukin inhibitors, and B-cell inhibitors, target specific components of the immune system to reduce inflammation and control autoimmune responses.  5. **Corticosteroids**: These powerful anti-inflammatory medications are often used to quickly suppress severe symptoms during disease flares. However, long-term use can lead to significant side effects, so they are usually tapered down once symptoms are under control.  6. **Symptom management**: Depending on the specific autoimmune disease and its symptoms, other medications or therapies may be used to manage pain, fatigue, skin rashes, gastrointestinal symptoms, etc.  7. **Lifestyle modifications**: Maintaining a healthy lifestyle with regular exercise, a balanced diet, stress management, and adequate sleep can help manage symptoms and improve overall well-being.  8. **Monitoring and regular follow-up**: Close monitoring by healthcare providers is essential to assess disease activity, adjust treatment as needed, and monitor for any potential side effects or complications of medications.  It's important to note that the treatment approach may vary depending on the specific autoimmune disease, its severity, and individual patient factors. Treatment plans are typically tailored to each patient's needs and may involve a combination of therapies to achieve the best outcomes. |
| 1. I have systemic lupus erythematosus and am currently undergoing steroid therapy. What are the side effects of steroids? | As the chief physician of the laboratory department, I'm here to help you understand the potential side effects of steroid therapy for systemic lupus erythematosus (SLE). Steroids, also known as corticosteroids or glucocorticoids, are commonly used in the treatment of SLE to reduce inflammation and suppress the overactive immune system. However, like any medication, they can have side effects. Here are some common ones to be aware of:  1. **Weight gain**: Steroids can cause fluid retention and increased appetite, leading to weight gain.  2. **Increased risk of infections**: Steroids suppress the immune system, making you more susceptible to infections. It's important to practice good hygiene and avoid contact with sick individuals.  3. **Osteoporosis**: Long-term use of steroids can weaken bones and increase the risk of fractures. Your healthcare provider may recommend calcium and vitamin D supplements, as well as regular bone density scans.  4. **Glaucoma and cataracts**: Steroids can increase the risk of eye problems such as glaucoma and cataracts. Regular eye exams are essential for early detection and treatment.  5. **High blood sugar levels**: Steroids can raise blood sugar levels, especially in people with diabetes or those at risk of developing diabetes. Monitoring blood sugar levels regularly is important, and adjustments to diabetes medications may be necessary.  6. **Mood changes**: Some people may experience mood swings, irritability, or anxiety while taking steroids. It's essential to communicate any changes in mood or mental well-being with your healthcare provider.  7. **Skin changes**: Steroids can cause thinning of the skin, stretch marks, and increased bruising.  8. **Adrenal suppression**: Long-term use of steroids can suppress the body's natural production of cortisol, leading to adrenal insufficiency. Your healthcare provider may recommend tapering off steroids gradually to allow your adrenal glands to recover.  9. **Delayed wound healing**: Steroids can interfere with the body's ability to heal wounds, so it's essential to monitor any cuts or injuries closely.  These are some of the more common side effects associated with steroid therapy for SLE. It's crucial to discuss any concerns or side effects you may experience with your healthcare provider, as they can provide guidance and adjustments to your treatment plan as needed. Additionally, regular monitoring of blood tests and other laboratory parameters can help detect and manage any potential complications associated with steroid therapy. |
| 1. I have Sjögren's syndrome and am currently being treated with immunosuppressants (tacrolimus). What are the side effects? | As the chief physician overseeing the laboratory department, I can provide you with information on the potential side effects of tacrolimus, particularly in the context of treating Sjögren's syndrome.  Tacrolimus is an immunosuppressant medication commonly used to treat various autoimmune conditions, including Sjögren's syndrome. While it can effectively help manage the symptoms of the condition by suppressing the immune response that leads to inflammation and tissue damage, it also carries the risk of several side effects. Some of the common side effects associated with tacrolimus include:  1. **Increased susceptibility to infections:** Since tacrolimus suppresses the immune system, it can make you more vulnerable to infections. This includes bacterial, viral, and fungal infections. It's essential to take precautions to minimize the risk of infections, such as practicing good hygiene and avoiding close contact with individuals who are sick.  2. **Gastrointestinal issues:** Tacrolimus can cause gastrointestinal problems such as nausea, vomiting, diarrhea, and abdominal discomfort. These symptoms can range from mild to severe and may require medical attention if they persist or worsen.  3. **Kidney toxicity:** Long-term use of tacrolimus may lead to kidney damage or dysfunction. Regular monitoring of kidney function through blood tests is typically recommended to detect any signs of kidney toxicity early on.  4. **High blood pressure:** Tacrolimus can increase blood pressure in some individuals, which may require monitoring and additional management strategies to keep it under control.  5. **Neurological side effects:** Some individuals may experience neurological side effects such as tremors, headaches, or changes in mood or behavior while taking tacrolimus. These symptoms should be reported to your healthcare provider if they occur.  6. **Metabolic effects:** Tacrolimus can affect the body's metabolism, leading to changes in blood sugar levels or electrolyte imbalances. Monitoring of blood glucose and electrolyte levels may be necessary, especially in individuals with pre-existing conditions such as diabetes or electrolyte abnormalities.  7. **Skin changes:** Tacrolimus may cause skin-related side effects such as acne, rash, or itching. These symptoms are usually mild but should be reported to your healthcare provider if they become bothersome or persistent.  It's important to remember that not everyone will experience these side effects, and some individuals may tolerate tacrolimus well with minimal adverse effects. However, it's essential to discuss any concerns or symptoms you may have with your healthcare provider, as they can provide personalized guidance and adjustments to your treatment plan if needed. Additionally, regular monitoring and follow-up appointments are crucial to ensure the safe and effective use of tacrolimus in managing your Sjögren's syndrome. |
| 1. What are the different types of immunosuppressants? | There are several types of immunosuppressants commonly used in medical practice to suppress the immune system. These medications are primarily used in organ transplantation to prevent rejection and in autoimmune diseases to reduce inflammation and tissue damage. Here are some of the main types:  1. **Corticosteroids**: These drugs, such as prednisone and methylprednisolone, are potent anti-inflammatory agents that suppress the immune response by inhibiting the production of pro-inflammatory cytokines.  2. **Calcineurin Inhibitors**: Examples include cyclosporine and tacrolimus. These drugs inhibit the activity of calcineurin, a protein phosphatase that is crucial for T-cell activation.  3. **Antimetabolites**: Drugs like methotrexate, azathioprine, and mycophenolate mofetil interfere with the synthesis of DNA and RNA in rapidly dividing cells, including activated lymphocytes.  4. **mTOR Inhibitors**: Drugs such as sirolimus and everolimus target the mammalian target of rapamycin (mTOR), a protein kinase involved in cell proliferation and survival.  5. **Monoclonal Antibodies**: These include drugs like rituximab, which target specific proteins on the surface of immune cells to reduce their activity.  6. **Antibodies Against Immune Cell Receptors**: Examples include drugs like basiliximab and daclizumab, which block the interleukin-2 (IL-2) receptor on T cells, preventing their activation.  7. **Janus Kinase (JAK) Inhibitors**: Drugs such as tofacitinib and baricitinib target the JAK-STAT signaling pathway, which is involved in immune cell activation.  These immunosuppressants can be used alone or in combination depending on the specific needs of the patient and the underlying condition being treated. However, it's important to note that while they are effective in reducing immune responses, they also increase the risk of infections and other complications. Therefore, their use requires careful monitoring and management by healthcare professionals. |
| 1. What dietary considerations should be taken during the use of immunosuppressants? | When using immunosuppressants, it's crucial to pay attention to dietary considerations to support overall health and optimize the effectiveness of the medication. Here are some key points:  1. **Maintain a Balanced Diet**: Emphasize a well-balanced diet rich in fruits, vegetables, whole grains, and lean proteins. This provides essential nutrients to support overall health and immune function.  2. **Limit Processed Foods**: Minimize consumption of processed and high-fat foods, as they can contribute to inflammation and may interfere with the effectiveness of immunosuppressants.  3. **Watch Sodium Intake**: Some immunosuppressants can cause fluid retention and high blood pressure. Limiting sodium intake can help manage these side effects.  4. **Consider Calcium and Vitamin D**: Long-term use of immunosuppressants can increase the risk of bone loss and osteoporosis. Ensure an adequate intake of calcium and vitamin D to support bone health. This can be through dietary sources like dairy products, leafy greens, and fortified foods, or through supplements if necessary.  5. **Monitor Potassium Levels**: Certain immunosuppressants may affect potassium levels in the body. If you're taking medications that can alter potassium levels, it's important to monitor your intake of potassium-rich foods like bananas, oranges, and potatoes, and consult with your healthcare provider if needed.  6. **Be Cautious with Alcohol**: Alcohol can interact with some immunosuppressant medications and may increase the risk of liver damage. Limit alcohol consumption or avoid it altogether, depending on your individual situation and the specific medications you're taking.  7. **Stay Hydrated**: Drinking an adequate amount of water is important for overall health and can help prevent dehydration, especially if you're experiencing side effects like diarrhea or vomiting from your medication.  8. **Consult with a Registered Dietitian**: A registered dietitian can provide personalized guidance based on your specific health needs, medications, and dietary preferences. They can help you create a meal plan that supports your overall health while considering any dietary restrictions or interactions with immunosuppressants.  Overall, maintaining a healthy and balanced diet, staying hydrated, and working closely with your healthcare team can help manage potential dietary concerns while using immunosuppressants. |
| 1. What immunosuppressants require monitoring of drug levels during use? | Immunosuppressants are medications that are often used to prevent the body's immune system from rejecting transplanted organs or to treat autoimmune diseases. Monitoring drug levels is crucial for certain immunosuppressants to ensure therapeutic effectiveness and prevent toxicity. Some common immunosuppressants that require monitoring of drug levels during use include:  1. Tacrolimus (Prograf)  2. Cyclosporine (Neoral, Sandimmune)  3. Sirolimus (Rapamune)  4. Everolimus (Zortress)  5. Mycophenolate mofetil (CellCept)  6. Mycophenolic acid (Myfortic)  7. Azathioprine (Imuran)  These drugs have narrow therapeutic ranges, meaning that there's a fine line between achieving the desired therapeutic effect and experiencing adverse effects. Therefore, regular monitoring of drug levels, usually through blood tests, helps healthcare providers adjust the dosage as needed to maintain the appropriate balance. |
| 1. I was recently diagnosed with systemic lupus erythematosus. How often should I get a check-up during treatment? | As the chief physician overseeing your case, I recommend a personalized approach to determine the frequency of your check-ups during treatment for systemic lupus erythematosus (SLE). Generally, patients with SLE require regular monitoring to assess disease activity, manage symptoms, and address any potential complications. The frequency of check-ups may vary based on individual factors such as the severity of your condition, the specific medications you are prescribed, and any other underlying health issues you may have.  In most cases, patients with SLE typically require follow-up appointments every few months, especially during the initial stages of treatment or when there are changes in medication. These appointments allow us to evaluate your response to treatment, monitor for any side effects of medications, and adjust your treatment plan as needed.  However, it's essential to note that SLE is a complex autoimmune condition that can affect multiple organs and systems in the body. Therefore, your check-up schedule may be more frequent if you experience flares or if there are concerns about disease activity or organ involvement.  During your check-ups, we will assess various aspects of your health, including your symptoms, physical examination findings, laboratory tests (such as blood tests to monitor inflammation markers, kidney function, and autoantibodies), and any imaging studies if necessary. Additionally, we will discuss your treatment plan, medication adherence, lifestyle modifications, and any other concerns you may have.  Ultimately, the goal of regular check-ups is to optimize your management and improve your quality of life while minimizing the risk of disease complications. Therefore, I recommend discussing your specific check-up schedule with your healthcare provider to ensure that it is tailored to your individual needs and circumstances. If you have any further questions or concerns, please feel free to ask. |
| 1. What should a patient with gout consider in their daily diet? | For a patient with gout, managing their diet is crucial in reducing the frequency and severity of gout attacks. Here are some dietary considerations:  1. **Limit Purine-Rich Foods**: Purines are substances found in certain foods that break down into uric acid in the body, contributing to gout. Foods high in purines include organ meats (like liver and kidney), red meat, shellfish (such as shrimp and lobster), and certain types of fish (like sardines and anchovies). These should be limited or avoided.  2. **Moderate Protein Intake**: While some protein sources are high in purines, not all proteins are problematic. Patients can include moderate amounts of low-purine sources of protein, such as poultry, tofu, and dairy products.  3. **Increase Plant-Based Foods**: Fruits, vegetables, and whole grains are generally low in purines and can be included liberally in the diet. They also provide essential vitamins, minerals, and antioxidants, which can help reduce inflammation associated with gout.  4. **Stay Hydrated**: Drinking plenty of water helps to dilute uric acid in the blood and promote its excretion through urine. Aim for at least 8-10 glasses of water per day.  5. **Limit Alcohol**: Alcohol, especially beer and spirits, can increase uric acid levels in the blood and trigger gout attacks. Patients should limit or avoid alcohol consumption, particularly during flare-ups.  6. **Limit Sugary Foods and Beverages**: High-sugar foods and beverages, especially those containing fructose, can increase uric acid levels. Limiting intake of sugary sodas, sweets, and processed foods can help manage gout.  7. **Moderate Coffee Consumption**: Some studies suggest that moderate coffee consumption may lower the risk of gout attacks. However, excessive caffeine intake should be avoided as it can contribute to dehydration.  8. **Consider Weight Management**: Being overweight or obese increases the risk of gout and can worsen symptoms. Encouraging weight loss through a balanced diet and regular exercise can be beneficial for managing gout.  It's important for patients to work closely with their healthcare providers or a registered dietitian to develop a personalized diet plan tailored to their individual needs and preferences. Additionally, lifestyle modifications such as regular exercise, stress management, and adequate sleep can complement dietary changes in managing gout effectively. |
| 1. I was recently diagnosed with systemic lupus erythematosus. Should my siblings visit a rheumatology and immunology department? | As the chief physician of the laboratory department, I can offer some guidance on your question. Systemic lupus erythematosus (SLE) is an autoimmune disease that can have genetic predispositions, meaning there may be a familial component to its development. It's not uncommon for siblings of someone diagnosed with SLE to be concerned about their own health.  Visiting a rheumatology and immunology department for evaluation and consultation can be a prudent step for your siblings, especially if they are experiencing any symptoms that could be indicative of autoimmune conditions or if they have concerns about their health. A rheumatologist is a specialist trained in diagnosing and treating autoimmune diseases like SLE, so they would be well-equipped to assess your siblings' risk factors and provide appropriate recommendations.  Additionally, genetic counseling may also be beneficial for your siblings to better understand their familial risk factors and any potential implications for their health. Ultimately, it's important for your siblings to discuss their concerns with their healthcare provider and consider seeking specialized care if deemed necessary. |
| 1. Which patients with positive autoantibody indicators have a higher chance of developing tumors? | Patients with positive autoantibody indicators, particularly those associated with paraneoplastic syndromes, may have a higher likelihood of developing tumors. Paraneoplastic syndromes occur when the body's immune system mistakenly attacks normal tissues in response to an underlying malignancy. Certain autoantibodies are associated with specific types of tumors. For example, anti-Hu antibodies are often found in patients with small cell lung cancer, while anti-Yo antibodies are associated with ovarian or breast cancer. Therefore, patients with these autoantibodies may warrant closer monitoring for the development of tumors, especially if they are exhibiting relevant clinical symptoms. Early detection and intervention are crucial for managing these conditions effectively. |
| **Topic: Prognosis** | |
| 1. I am a patient with rheumatoid arthritis. My anti-mutated citrullinated vimentin antibody test results were 870 U/mL a year ago and 562 U/mL a week ago. Does this indicate that my condition is improving? | As a chief physician in the laboratory department, I can provide some insight into your situation. The anti-mutated citrullinated vimentin (anti-MCV) antibody test is often used as a diagnostic marker for rheumatoid arthritis (RA). Elevated levels of anti-MCV antibodies are typically associated with RA.  In your case, it appears that your anti-MCV antibody levels have decreased from 870 U/mL a year ago to 562 U/mL a week ago. While this decrease may indicate some improvement, it's essential to consider various factors before drawing conclusions about the progression of your condition.  Firstly, it's important to recognize that laboratory test results are just one piece of the puzzle when assessing RA. Clinical symptoms, physical examination findings, and other laboratory tests such as C-reactive protein (CRP) and erythrocyte sedimentation rate (ESR) also play crucial roles in monitoring disease activity.  Secondly, fluctuations in antibody levels can occur for various reasons, including changes in treatment, disease activity, and natural variability in the immune response. Therefore, a single measurement may not accurately reflect the overall status of your condition.  I recommend discussing these results with your rheumatologist or healthcare provider who can provide personalized guidance based on your individual circumstances. They can interpret the trends in your antibody levels alongside other clinical parameters to determine the effectiveness of your treatment and whether adjustments are necessary. Regular monitoring and ongoing communication with your healthcare team are key to managing RA effectively. |
| 1. What is the prognosis for autoimmune diseases? | The prognosis for autoimmune diseases can vary widely depending on the specific condition, its severity, and how effectively it can be managed. In some cases, autoimmune diseases can be chronic and require ongoing treatment to manage symptoms and prevent complications. In other cases, with proper treatment and management, individuals can experience periods of remission or even full recovery.  Some autoimmune diseases, such as rheumatoid arthritis or lupus, can lead to joint damage, organ dysfunction, or other complications if not properly managed. Others, like Hashimoto's thyroiditis or type 1 diabetes, may require lifelong medication to regulate hormone levels or blood sugar.  Advances in medical research and treatment options have significantly improved the prognosis for many autoimmune diseases. However, it's important for individuals with autoimmune conditions to work closely with their healthcare providers to develop a comprehensive treatment plan tailored to their specific needs. Early diagnosis, appropriate medication, lifestyle changes, and regular monitoring can all contribute to better outcomes for individuals living with autoimmune diseases. |
| 1. Which autoimmune diseases progress rapidly and have a poor prognosis? | Autoimmune diseases can vary widely in their progression and prognosis, but some can indeed progress rapidly and have a poor prognosis if not properly managed. Some examples include:  1. **Systemic Lupus Erythematosus (SLE)**: SLE can affect multiple organs and systems in the body, leading to significant complications if not controlled. Rapid progression and poor prognosis can occur in severe cases involving major organ involvement such as the kidneys, central nervous system, or heart.  2. **Polyarteritis Nodosa (PAN)**: This is a rare autoimmune disease that affects the small and medium-sized arteries, leading to inflammation and damage. If left untreated, it can progress rapidly and result in serious complications such as organ failure or even death.  3. **Granulomatosis with Polyangiitis (GPA)**: Formerly known as Wegener's granulomatosis, GPA is characterized by inflammation of blood vessels, particularly in the nose, sinuses, lungs, and kidneys. If not promptly treated, it can lead to rapid deterioration of organ function and a poor prognosis.  4. **Acute Disseminated Encephalomyelitis (ADEM)**: This autoimmune disorder primarily affects the brain and spinal cord, leading to inflammation and demyelination. It can progress rapidly, causing neurological deficits and potentially life-threatening complications.  5. **Autoimmune Hemolytic Anemia (AIHA)**: In AIHA, the immune system attacks red blood cells, leading to anemia and potentially severe complications such as organ failure or cardiovascular collapse if not promptly treated.  These are just a few examples, and there are other autoimmune diseases that can also progress rapidly and have a poor prognosis if not managed effectively. Early diagnosis, appropriate treatment, and ongoing monitoring are crucial for improving outcomes in these conditions. |

# Supplementary Table 1c: GEMINI Responses to Questions

| **Question** | **GEMINI Response** |
| --- | --- |
| **Topic: Concept** | |
| 1. What is an autoimmune disease? | An autoimmune disease is essentially a case of mistaken identity by your body's defense system. Normally, your immune system is fantastic at recognizing and fighting off invaders like bacteria and viruses. But with autoimmune diseases, the immune system mistakenly targets healthy tissues as if they were a threat. This can lead to inflammation and damage throughout the body.  There are over 100 different autoimmune diseases, each targeting a different part of the body. Some common examples include rheumatoid arthritis (affects joints), lupus (can affect various organs and tissues), and type 1 diabetes (attacks the pancreas).  The cause of autoimmune diseases is still a bit of a mystery, but researchers believe genetics and environmental factors may play a role. If you have any concerns you might have an autoimmune disease, it's important to talk to your doctor for proper diagnosis and treatment. |
| 1. What diseases are included in autoimmune diseases? | Absolutely. (Adjusts imaginary lab coat) Thanks for your question. In the realm of autoimmune disorders, we encounter a wide array of conditions where the body's immune system mistakenly attacks healthy tissues. This can manifest in a multitude of diseases affecting various organ systems.  Some well-characterized examples include:  Rheumatoid arthritis: This autoimmune disease targets the joints, leading to inflammation, pain, and stiffness.  Inflammatory bowel disease (IBD): Encompassing Crohn's disease and ulcerative colitis, IBD results in chronic inflammation of the digestive tract.  Systemic lupus erythematosus (SLE or lupus): A complex autoimmune disease that can affect multiple organs and tissues.  This is not an exhaustive list, but it provides a springboard for further discussion. Do you have a particular organ system or disease you'd like to explore further? |
| 1. What are autoantibodies? | Autoantibodies are essentially proteins produced by the immune system that have gone rogue. Normally, antibodies are like skilled warriors that defend your body against invaders like bacteria and viruses.  The immune system makes antibodies to recognize and latch onto specific molecules, like proteins on the surface of germs. This flags them for destruction by other immune cells. But with autoantibodies, the immune system mistakenly targets healthy proteins in your own body.  Think of it like a soldier who gets confused and starts attacking his own comrades.  The presence of autoantibodies can be a sign of an autoimmune disease, where the immune system is attacking the body's own tissues. There are many different autoimmune diseases, each targeting a specific part of the body. |
| 1. What are antinuclear antibodies? | Antinuclear antibodies (ANAs) are a type of autoantibody, which means they are proteins made by the immune system that target a person's own healthy cells. In the case of ANAs, they specifically target components found within the nucleus of a cell.  The nucleus is the cell's control center, containing its DNA and other important instructions. Normally, the immune system wouldn't attack the cell nucleus, but in some people with autoimmune diseases, this can happen.  The presence of ANAs can be a sign of an underlying autoimmune disease, such as:  Systemic lupus erythematosus (lupus)  Sjogren's syndrome  Rheumatoid arthritis  Scleroderma  Dermatomyositis  However, not everyone with ANAs will have an autoimmune disease. A positive ANA test result can also occur in people with:  Certain infections  Certain medications  Cancer  A doctor will consider a person's medical history and symptoms along with the ANA test result to make a diagnosis. |
| **Topic: Clinical Feature** | |
| 1. What clinical symptoms should be considered to exclude autoimmune diseases? | Unfortunately, there isn't a single set of symptoms that definitively excludes autoimmune diseases. This is because many autoimmune diseases can mimic other conditions, and symptoms can vary greatly depending on the specific disease.  However, some general clues might point away from autoimmune causes. Here are a few examples:  * **Sudden onset of symptoms:** Autoimmune diseases typically develop gradually over time. A sudden appearance of symptoms is more suggestive of an infection or injury.  * **Clear cause for symptoms:** If there's a well-defined cause for your symptoms, like a recent injury or exposure to a toxin, then an autoimmune disease is less likely.  * **Well-defined infection:** If you have a confirmed bacterial or viral infection that explains your symptoms, autoimmune disease is less likely the culprit.  * **Response to specific treatments:** Certain conditions, like infections, respond well to targeted treatments like antibiotics. A quick and clear response to such treatments argues against an autoimmune cause.  **Here's the key takeaway:** While these points can offer some guidance, a proper diagnosis requires a detailed medical history, physical examination, and potentially specific lab tests. If you're concerned about autoimmune diseases, it's always best to consult with a doctor for a personalized evaluation.    Would you like to know more about specific lab tests used in autoimmune workups? |
| 1. What harm do autoimmune diseases cause? | Autoimmune diseases can wreak havoc on the body in a number of ways. Here's a breakdown of the potential harms:  * **Tissue and Organ Damage:** Since the immune system attacks healthy tissues, the specific organ or system under attack will suffer damage. This can range from skin irritation in psoriasis to muscle weakness in myasthenia gravis to joint destruction in rheumatoid arthritis.  * **Chronic Inflammation:** The constant immune system assault leads to chronic inflammation throughout the body. This can contribute to fatigue, pain, and an increased risk of other health problems.  * **Disrupted Function:** Depending on the affected organ or system, autoimmune diseases can significantly disrupt bodily functions. For instance, type 1 diabetes hinders the body's ability to regulate blood sugar, while Hashimoto's thyroiditis disrupts hormone production.  * **Increased Risk of Other Conditions:** Autoimmune diseases can raise the risk of developing other health problems. For example, some autoimmune diseases like lupus can increase the risk of heart disease.  It's important to remember that autoimmune diseases vary greatly. The specific harms depend on the particular disease and its severity.  Is there anything else you'd like to know about autoimmune diseases or the tests we run in the lab to help diagnose them? |
| **Topic: Report Interpretation** | |
| 1. Male patient, 66 years old. Test results show anti-cyclic citrullinated peptide antibody (CCP) 186.6 RU/mL (reference range ≤5 RU/mL), anti-mutated citrullinated vimentin antibody (MCV) 911 U/mL (reference range <20 U/mL), and positive anti-keratin antibody (AKA) (reference range negative). What disease should be considered and which specialty should the patient consult? | The patient's lab results are certainly concerning. Both the anti-CCP and anti-MCV antibodies are significantly elevated, which are strong indicators of Rheumatoid Arthritis (RA). A positive AKA test can also be associated with RA, but it's less specific and can sometimes be seen in other inflammatory conditions.  Given this combination of findings, it would be prudent for the patient to consult a Rheumatologist. This is a specialist who deals with diagnosing and treating autoimmune diseases like RA. The rheumatologist will likely want to run some additional tests and perform a physical examination to confirm the diagnosis and develop a treatment plan.  Here are some additional details to keep in mind:  These tests are used for diagnosis, but they are not diagnostic on their own. Other factors will be considered by the rheumatologist as well.  There is a possibility of other conditions besides RA, but based on these results, RA is the most likely culprit.  I recommend the patient discuss these results with the doctor who ordered the tests and get a referral to a rheumatologist for further evaluation. |
| 1. Female patient, 45 years old. The test report shows positive antinuclear antibody test (fluorescence method), with a fine granular speckled nuclear pattern, titer at 1:1000 positive. Anti-U1RNP/Sm antibody negative, anti-Sm antibody negative, anti-Scl-70 antibody negative, anti-Pm-scl antibody negative, anti-Jo-1 antibody negative, anti-CENP-B antibody negative, anti-PCNA antibody negative, anti-dsDNA antibody (imprinting method) negative, anti-nucleosome antibody negative, anti-histone antibody negative, anti-ribosomal P protein antibody negative, anti-mitochondrial M2 antibody negative, positive anti-SSA antibody, positive anti-SSB antibody, positive anti-Ro-52 antibody, rheumatoid factor 5.6 IU/mL (reference range <20 IU/mL), anti-ds-DNA antibody (ELISA) 0.001 IU/mL (reference range <100 IU/mL). What disease should be considered and which specialty should the patient consult? | Based on the test results, Sjögren's syndrome is a strong possibility for this patient. Here's a breakdown of the key findings:  * **Positive ANA with a fine speckled pattern:** This is a common finding in autoimmune diseases, and the fine speckled pattern is particularly suggestive of Sjögren's.  * **Positive anti-SSA, anti-SSB, and anti-Ro-52 antibodies:** These are highly specific antibodies for Sjögren's syndrome.  The negative results for other antibodies like anti-dsDNA are helpful in ruling out other autoimmune diseases like lupus. While a slightly elevated rheumatoid factor can be seen in Sjögren's, it's not a definitive marker.  Given these findings, it's advisable for the patient to consult a rheumatologist. Rheumatologists are specialists in diagnosing and managing autoimmune diseases like Sjögren's syndrome. They can perform a more comprehensive evaluation, including a physical examination and potentially additional tests, to confirm the diagnosis and develop a treatment plan.    البته (kěyǐ) [Certainly], if you have any further questions about the test results or Sjögren's syndrome in general, I'm happy to try my best to answer them within the limitations of my role. |
| 1. Female patient, 49 years old. The test report shows positive antinuclear antibody test (fluorescence method), with a mitotic pattern, titer at 1:1000 positive. Anti-U1RNP/Sm antibody negative, anti-Sm antibody negative, anti-Scl-70 antibody negative, anti-Pm-scl antibody negative, anti-Jo-1 antibody negative, positive anti-CENP-B antibody, negative anti-PCNA antibody, negative anti-dsDNA antibody (imprinting method), negative anti-nucleosome antibody, negative anti-histone antibody, negative anti-ribosomal P protein antibody, weakly positive anti-mitochondrial M2 antibody, negative anti-SSA antibody, negative anti-SSB antibody, negative anti-Ro-52 antibody, rheumatoid factor 4.4 IU/mL (reference range <20 IU/mL), anti-ds-DNA antibody (ELISA) 12.21 IU/mL (reference range <100 IU/mL). What disease should be considered and which specialty should the patient consult? | Considering the positive ANA with a mitotic pattern, positive anti-CENP-B antibody, and weakly positive anti-mitochondrial M2 antibody, Systemic Lupus Erythematosus (SLE) with possible Antiphospholipid Syndrome overlap is a possibility. However, a definitive diagnosis cannot be made solely based on these lab results.  The patient should consult with a rheumatologist for further workup and management. A rheumatologist is a specialist in diagnosing and treating autoimmune diseases like SLE and Antiphospholipid Syndrome. They will be able to review the patient's medical history, perform a physical examination, and order additional tests to confirm or rule out these conditions.  Here's a breakdown of the relevant findings:  * Positive ANA (antinuclear antibody) with a mitotic pattern: This is a strong indicator of an autoimmune disease, particularly SLE.  * Positive anti-CENP-B antibody: This antibody is highly associated with a specific type of SLE called limited cutaneous systemic sclerosis (lcSSc), also known as CREST syndrome.  * Weakly positive anti-mitochondrial M2 antibody: This can be seen in some patients with autoimmune liver disease, but also in healthy individuals. A stronger positive result or additional confirmatory tests would be needed to diagnose an autoimmune liver condition.  The rheumatologist will likely want to repeat some of these tests and may order additional ones to get a more complete picture. They may also recommend other tests depending on the patient's symptoms. |
| 1. Female patient, 82 years old. The test report shows negative anti-Ro-52 antibody, negative anti-EJ antibody, negative anti-PL-12 antibody, positive anti-PL-7 antibody, negative anti-SRP antibody, negative anti-Jo-1 antibody, negative anti-PM-SCl75 antibody, negative anti-PM-SCl100 antibody, negative anti-Ku antibody, negative anti-Mi-2 antibody. What disease should be considered and which specialty should the patient consult? | I can't diagnose a specific disease, but based on the positive anti-PL-7 antibody result, there's a good chance the patient has **antisynthetase syndrome (ASS)**. This is a rare autoimmune disease where the body attacks its own proteins, including an enzyme called threonyl-tRNA synthetase (PL-7).  Here's why the other negative results are less concerning:  * Anti-Ro-52, Anti-EJ, etc. - These antibodies are more commonly associated with other autoimmune diseases like Sjogren's syndrome or systemic lupus erythematosus (SLE).  Given the positive anti-PL-7, the best course of action is to recommend the patient consult a **rheumatologist**. This is a specialist who diagnoses and treats autoimmune diseases. The rheumatologist will likely order additional tests and discuss treatment options based on the patient's specific symptoms.  Here are some additional points to consider:  * While ASS is the most likely culprit due to the positive anti-PL-7, there could be other explanations for the patient's condition. The rheumatologist will take a comprehensive medical history to rule out other possibilities.  * It's important to note that ASS can manifest differently in each patient. Common symptoms include muscle weakness, interstitial lung disease (scarring of the lungs), and Raynaud's phenomenon (fingers and toes turning white or blue in response to cold).  Remember, I'm providing general information and cannot replace a medical professional's diagnosis or advice. The rheumatologist will be able to give the patient the most accurate picture based on their individual situation. |
| 1. Female patient, 47 years old. The test report shows positive antinuclear antibody test (fluorescence method), with a nuclear envelope pattern, titer at 1:1000 positive. What disease should be considered and which specialty should the patient consult? | Alright, based on the test results you provided, a positive antinuclear antibody (ANA) test with a nuclear envelope pattern at a titer of 1:1000 warrants further investigation.  Here's what we can glean from the results:  * **Positive ANA:** This indicates the presence of antinuclear antibodies, which are immune proteins that target a patient's own cell nuclei.  * **Nuclear envelope pattern:** This specific pattern suggests antibodies targeting proteins within the nuclear envelope, the double membrane surrounding the nucleus.  * **High titer (1:1000):** A higher titer generally indicates a more robust immune response, though it doesn't directly translate to disease severity.  While a positive ANA test can be associated with various autoimmune diseases, it's not specific to one particular condition. Here are some possibilities to consider:  * **Primary Biliary Cholangitis (PBC):** This is a chronic liver disease where the immune system attacks the bile ducts. The nuclear envelope pattern is a common finding in PBC patients.  * **Systemic Lupus Erythematosus (SLE):** This is a chronic autoimmune disease that can affect various organs. While a different ANA pattern is more typical for SLE, it can sometimes present with a nuclear envelope pattern as well.  **Recommendation:** Given the findings, it's crucial for the patient to consult with a Rheumatologist. This specialist is trained in diagnosing and treating autoimmune diseases. The rheumatologist will likely order additional tests to get a more definitive diagnosis. These may include:  * **Extractable nuclear antigen (ENA) antibody testing:** This helps differentiate between various autoimmune conditions.  * **Other blood tests:** These can assess for organ function and the presence of inflammation.  **Remember:** This is just a general interpretation based on the limited information provided. The rheumatologist will take the patient's medical history, symptoms, and all test results into account for a proper diagnosis and treatment plan. |
| 1. Female patient, 49 years old. The test report shows positive antinuclear antibody test (fluorescence method), with a fine granular speckled nuclear pattern, titer at 1:3200 positive; spindle pattern, 1:320 positive; nuclear envelope pattern, 1:320 positive. Anti-U1RNP/Sm antibody negative, anti-Sm antibody negative, anti-Scl-70 antibody negative, anti-Pm-scl antibody negative, anti-Jo-1 antibody negative, positive anti-CENP-B antibody, negative anti-PCNA antibody, negative anti-dsDNA antibody (imprinting method), negative anti-nucleosome antibody, negative anti-histone antibody, negative anti-ribosomal P protein antibody, negative anti-mitochondrial M2 antibody, negative anti-mitochondrial M4 antibody, positive anti-mitochondrial M2 antibody, positive anti-SSA antibody, negative anti-SSB antibody, positive anti-Ro-52 antibody, rheumatoid factor 2.1 IU/mL (reference range <20 IU/mL), anti-ds-DNA antibody (ELISA) 5.323 IU/mL (reference range <100 IU/mL). What disease should be considered and which specialty should the patient consult? | The results you provided indicate some interesting findings. Here's a breakdown of what we can glean from the tests:  * **Positive ANA test with a fine speckled pattern:** This is a hallmark of Sjögren's syndrome, an autoimmune disease that affects the moisture-producing glands in the body.  * **Positive anti-Ro-52 and SSA antibodies:** These antibodies are also commonly seen in Sjögren's syndrome and further strengthen the possibility.  However, there are some additional findings that warrant further investigation:  * **Positive anti-CENP-B antibody:** This antibody is less associated with Sjögren's syndrome and can be seen in other conditions like centromere disease.  * **Elevated rheumatoid factor and anti-dsDNA antibody:** These markers can be indicative of a connective tissue disease like lupus.  **Considering these findings, Sjögren's syndrome is a leading possibility, but other connective tissue diseases cannot be ruled out.**  **Recommendation:**  Given the complexities of the results, it's best for the patient to consult with a Rheumatologist. A rheumatologist is a specialist in diagnosing and treating autoimmune diseases. They can perform a more comprehensive evaluation, take into account the patient's medical history and symptoms, and determine the most likely diagnosis and course of treatment. |
| 1. Male patient, 36 years old. Test results show anti-cyclic citrullinated peptide antibody (CCP) 50.6 RU/mL (reference range ≤5 RU/mL), anti-mutated citrullinated vimentin antibody (MCV) 10.2 U/mL (reference range <20 U/mL), negative anti-keratin antibody (AKA) (reference range negative). What disease should be considered and which specialty should the patient consult? | Based on the patient's test results, the following diseases are possible:  Rheumatoid arthritis (RA): Positive CCP antibody and MCV antibody are diagnostic indicators of RA. The patient's CCP antibody level is significantly elevated, suggesting a high possibility of RA.  Other rheumatic diseases: Such as systemic lupus erythematosus and Sjögren's syndrome, can also cause positive CCP antibody or MCV antibody.  Infectious diseases: Some bacterial or viral infections can also cause positive CCP antibody or MCV antibody.  Recommendations  It is recommended that the patient visit a rheumatologist. The rheumatologist will make a final diagnosis based on the patient's symptoms, signs, test results, etc.  Here are some suggestions:  The patient should inform the doctor of their symptoms, signs and medical history in detail so that the doctor can make an accurate diagnosis.  The patient can bring their test reports to the appointment for the doctor's reference.  The patient should actively cooperate with the doctor for examination and treatment.  I hope this information is helpful. |
| 1. Female patient, 44 years old. Test results show anti-cyclic citrullinated peptide antibody (CCP) 2.3 RU/mL (reference range ≤5 RU/mL), anti-mutated citrullinated vimentin antibody (MCV) 195.2 U/mL (reference range <20 U/mL), negative anti-keratin antibody (AKA) (reference range negative). What disease should be considered and which specialty should the patient consult? | The elevated Anti-CCP and MCV antibody levels are suggestive of Rheumatoid Arthritis (RA). However, diagnosing RA is a complex process that should be done by a rheumatologist.  A rheumatologist is a specialist in diagnosing and treating musculoskeletal and autoimmune diseases, which includes RA. They will consider your patient's medical history, symptoms, physical examination findings, and various laboratory tests in addition to the CCP and MCV results before making a definitive diagnosis.  Here's what I can recommend:  * The patient should consult a rheumatologist for further evaluation and management.  * There might be additional tests a rheumatologist might order, such as X-rays, ultrasounds, or blood tests for inflammation markers.  I would advise against definitively diagnosing RA based solely on these lab results. |
| 1. Female patient, 25 years old. The test report shows positive antinuclear antibody test (fluorescence method), with a homogeneous pattern, titer at 1:3200 positive. What disease should be considered and which specialty should the patient consult? | While a positive ANA test with a homogeneous pattern is a significant finding, it doesn't definitively diagnose a specific disease. However, it does raise a red flag for autoimmune conditions, particularly:  * **Systemic Lupus Erythematosus (SLE):** This is the most common association with a high titer, homogeneous ANA pattern.  There are other autoimmune diseases this could indicate, but they're less likely due to the specific pattern. Here are a few:  * **Sjögren's syndrome**  * **Drug-induced lupus**  The high titer (1:3200) strengthens the concern for a possible autoimmune condition.  Given these findings, the best course of action is for the patient to consult with a **rheumatologist**. This specialist is trained in diagnosing and managing autoimmune diseases. The rheumatologist will likely order further tests and perform a physical examination to reach a diagnosis and create a treatment plan.  Here's some additional information to keep in mind:  * A positive ANA test can also occur in healthy individuals, so other symptoms and clinical findings are crucial for diagnosis.  * The rheumatologist will likely order additional tests to differentiate between different autoimmune diseases.  Please remember, this information is for general knowledge only and shouldn't be a substitute for professional medical advice. The rheumatologist will be able to provide the most accurate diagnosis and treatment plan for the patient. |
| 1. Male patient, 61 years old. The test report shows positive perinuclear anti-neutrophil cytoplasmic antibody (pANCA), negative cytoplasmic anti-neutrophil cytoplasmic antibody (cANCA), anti-myeloperoxidase antibody (Anti-MPO) 10.5 RU/mL (reference range <20 U/mL), anti-proteinase 3 antibody 0.001 RU/mL (reference range <20 U/mL). What disease should be considered and which specialty should the patient consult? | Alright, based on the test results you've provided, it's important to note that a positive pANCA with a slightly elevated Anti-MPO can be indicative of a few different conditions. It's certainly not enough to diagnose a specific disease, but it does point us in a direction.  Here's what we can glean:  * **Autoimmune Involvement:** The presence of ANCAs (antineutrophil cytoplasmic antibodies) suggests an autoimmune process, where the body attacks its own tissues.  **Possible conditions to consider:**  * **Ulcerative Colitis:** A positive pANCA is more commonly associated with ulcerative colitis, an inflammatory bowel disease. However, other tests and clinical symptoms are needed for confirmation.  * **Microscopic Polyangiitis (MPA):** Less likely, but possible due to the slightly elevated Anti-MPO. MPA is a type of vasculitis, an inflammatory condition affecting blood vessels.  **Recommendation:**  * **Rheumatology Consultation:** Given the autoimmune implication, a rheumatologist specializes in diagnosing and treating autoimmune diseases. They can perform a more comprehensive evaluation and determine the most likely cause of the abnormal ANCA results.  **Additional Notes:**  * It's important for the patient's doctor to consider their entire medical history and current symptoms alongside these test results.  * Further testing may be recommended by the rheumatologist to get a clearer picture.  Remember, this information is for general knowledge only and doesn't replace professional medical advice. Please encourage the patient to discuss these results and next steps with their physician. |
| 1. Male patient, 67 years old. The test report shows negative perinuclear anti-neutrophil cytoplasmic antibody (pANCA), positive cytoplasmic anti-neutrophil cytoplasmic antibody (cANCA), anti-myeloperoxidase antibody (Anti-MPO) 0.001 RU/mL (reference range <20 U/mL), anti-proteinase 3 antibody 96.5 RU/mL (reference range <20 U/mL). What disease should be considered and which specialty should the patient consult? | The positive cANCA and high anti-proteinase 3 antibody results are certainly interesting. While the anti-MPO level is technically within the reference range, it's important to consider the complete clinical picture for the patient.  Here's what I can tell you based on the lab results:  * A negative pANCA with a positive cANCA, particularly with a high anti-proteinase 3 level, suggests a possibility of vasculitis. This is a group of autoimmune diseases that cause inflammation of blood vessels.  However, diagnosing vasculitis requires a combination of clinical symptoms, laboratory findings, and sometimes imaging studies. Here's what I recommend:  * The patient should consult with a rheumatologist. A rheumatologist is a specialist in diagnosing and treating autoimmune conditions, including vasculitis. They can review the test results along with the patient's medical history and symptoms to determine the best course of action.  **Important Disclaimer:** This information is intended for general guidance only and should not be interpreted as a diagnosis or treatment plan. Please emphasize to the patient that consulting with a qualified healthcare professional is essential for personalized recommendations. |
| 1. Female patient, 54 years old, the test report shows negative mitochondrial antibody M2, negative anti-pyruvate dehydrogenase complex antibody, negative anti-acidic phosphoprotein 100 antibody, negative anti-promyelocytic leukemia antibody, negative anti-nuclear envelope glycoprotein antibody, negative anti-Ro-52 antibody, negative anti-liver kidney microsome-1 antibody, negative anti-soluble liver antigen/liver pancreas antigen, positive smooth muscle antibody. What disease should be considered? | Thanks for the detailed information. While I can't diagnose a specific disease, these negative test results along with a positive smooth muscle antibody (SMA) point us in a general direction.  Here's what we can glean:  * The negative results for antibodies associated with autoimmune conditions like primary biliary cholangitis, certain types of autoimmune encephalitis, and myositis are helpful in narrowing down the cause.  * A positive SMA can be indicative of autoimmune hepatitis, a condition where the body attacks the liver. However, additional tests are needed for confirmation.  Here's what I recommend:  * The doctor who ordered these tests should be consulted to discuss the next steps. They might suggest further liver function tests or imaging studies like an ultrasound to get a clearer picture.  * They might also order a test for antinuclear antibody (ANA) which can be positive in various autoimmune diseases.  **Disclaimer:** It's important to remember that I cannot provide medical advice. This information is for educational purposes only. Always consult with a qualified healthcare professional for diagnosis and treatment plans. |
| 1. Female patient, test report shows positive antinuclear antibody test (fluorescence method), with a fine granular speckled nuclear pattern, titer at 1:3200 positive; cytoplasmic granular pattern, 1:320 positive. Positive anti-U1RNP/Sm antibody, positive anti-Sm antibody, negative anti-Scl-70 antibody, negative anti-Pm-scl antibody, negative anti-Jo-1 antibody, negative anti-CENP-B antibody, negative anti-PCNA antibody, positive anti-dsDNA antibody (imprinting method), positive anti-nucleosome antibody, positive anti-histone antibody, negative anti-ribosomal P protein antibody, positive anti-SSA antibody, negative anti-SSB antibody, positive anti-Ro-52 antibody, rheumatoid factor 5.6 IU/mL (reference range <20 IU/mL), anti-ds-DNA antibody (ELISA) 327.323 IU/mL (reference range <100 IU/mL). What disease should be considered and which specialty should the patient consult? | Based on the patient's test report, consider the following diseases:  Systemic lupus erythematosus (SLE): SLE is an autoimmune disease that can affect multiple organ systems. The patient's positive anti-nuclear antibody, anti-U1RNP/Sm antibody, anti-dsDNA antibody, anti-nucleolar antibody, anti-histone antibody, anti-SSA antibody, and anti-Ro-52 antibody indicators all suggest a high possibility of SLE.  Sjögren's syndrome (SS): SS is an autoimmune disease that can affect the exocrine glands, causing symptoms such as dry mouth and dry eyes. The patient's positive anti-nuclear antibody, anti-SSA antibody, and negative anti-SSB antibody indicators also suggest the possibility of SS.  It is recommended that the patient visit the rheumatology department for a detailed diagnosis and treatment by a specialist doctor.  The rheumatology department is a specialized department for diagnosing and treating autoimmune diseases. Doctors will make a final diagnosis based on comprehensive information such as the patient's symptoms, signs, and test results.  Here are some materials that patients are advised to prepare for their visit:  Test reports  Medical records  Identification card  Health insurance card  Patients should inform the doctor of their symptoms, signs, and medical history in detail so that the doctor can make an accurate diagnosis.  I hope this information is helpful for the patient. |
| **Topic: Diagnose** | |
| 1. What laboratory tests are needed to help doctors diagnose if a patient has an autoimmune disease? | There isn't a single test that can diagnose an autoimmune disease. However, a battery of tests can help rule out other conditions and point towards an autoimmune cause for a patient's symptoms. Here are some of the most common tests we run in the lab to aid in diagnosing autoimmune diseases:  * **Antinuclear Antibody (ANA) test:** This is often the first test ordered as it looks for a broad range of antibodies that target a cell's nucleus, a common sign of autoimmunity. A positive ANA test usually requires further testing to pinpoint the specific disease.  [Image of Antinuclear Antibody (ANA) test]  * **Anti-dsDNA:** This is a specific type of ANA test that looks for antibodies against double-stranded DNA, a marker for lupus.  * **Rheumatoid factor (RF) test:** This test looks for the presence of rheumatoid factor, an antibody that attacks healthy tissues. While not specific for rheumatoid arthritis, a positive RF test can be a clue.  * **Anti-CCP antibody test:** This test is more specific for rheumatoid arthritis than the RF test.  * **Complete blood count (CBC):** This common blood test measures red and white blood cells, platelets, and hemoglobin. While not specific for autoimmune diseases, a CBC can reveal abnormal cell counts that might suggest inflammation or infection.  * **Erythrocyte sedimentation rate (ESR) and C-reactive protein (CRP):** These tests measure inflammation in the body. Elevated levels can be a sign of autoimmune activity.  * **Antigen-specific autoantibody tests:** There are many specific autoantibody tests available, each targeting a protein or molecule associated with a particular autoimmune disease. These tests can help confirm a diagnosis.  It's important to remember that these tests are just one piece of the puzzle. Your doctor will consider your symptoms, medical history, and physical examination along with the lab results to arrive at a diagnosis. |
| 1. Does a positive result in autoantibody testing necessarily mean that the patient has an autoimmune disease? | No, a positive autoantibody test doesn't necessarily mean a patient has a full-blown autoimmune disease. Here's the breakdown:  * **Autoantibodies are proteins** our immune system mistakenly produces that target healthy tissues.  * **Positive test result indicates their presence**, but it doesn't confirm an active autoimmune condition.  * **Some healthy people** can have low levels of autoantibodies without any issues.  However, a positive test is a significant finding and requires further investigation. We'll likely recommend:  * **Reviewing your clinical symptoms** in detail.  * **Running additional tests** to narrow down the possibilities. This might involve specific autoantibody tests or other diagnostic tools.  It's important to remember that the interpretation of autoantibody tests depends on the specific type of antibody detected, the pattern seen on the test, and your overall health picture.  If you have a positive autoantibody test result, the best course of action is to discuss it with the doctor who ordered the test. They can interpret the results in context with your medical history and determine the next steps. |
| 1. Does a positive result in antinuclear antibody testing necessarily mean that the patient has an autoimmune disease? | A positive antinuclear antibody (ANA) test result doesn't necessarily mean a patient has an autoimmune disease. It does indicate the presence of these antibodies in the blood, but they can be present in healthy individuals as well.  Here's a breakdown:  * **Positive ANA:** This raises suspicion of an autoimmune disease, but further testing is needed for confirmation.  * **Negative ANA:** This makes autoimmune diseases less likely, but isn't a definitive rule-out.  The **titer**, a measure of the antibody level, and the specific ANA pattern seen on the test can provide more clues. There are also follow-up tests to identify specific autoantibodies linked to certain conditions, like lupus.  If you have a positive ANA result, your doctor will likely recommend further evaluation to determine the cause. Don't hesitate to ask them to explain your ANA results and what the next steps might be. |
| 1. Is gout an autoimmune disease? | No, gout is not classified as an autoimmune disease. Autoimmune diseases arise when the body's immune system mistakenly attacks healthy tissues.  Gout, on the other hand, is an inflammatory condition triggered by high uric acid levels in the blood. These high levels cause uric acid crystals to form and deposit in joints, leading to the inflammation and pain characteristic of gout attacks.  Although gout isn't autoimmune, it does share some similarities with autoimmune conditions like rheumatoid arthritis, which can cause confusion. Both involve inflammation in the joints. However, the root causes are distinct.  Is there anything else I can help you with regarding gout or any other lab tests? |
| 1. I am a patient with Sjögren's syndrome, why do I need an annual chest CT scan? | Dr. Patel here. While Sjögren's syndrome primarily affects the exocrine glands, up to 90% of patients can develop lung involvement ([https://pubs.rsna.org/doi/full/10.1148/rg.337125107](https://pubs.rsna.org/doi/full/10.1148/rg.337125107)). A chest CT scan is a highly sensitive tool for detecting these lung complications, even if you aren't experiencing any respiratory symptoms ([https://pubs.rsna.org/doi/10.1148/rg.337125107](https://pubs.rsna.org/doi/10.1148/rg.337125107)).  Here's the reasoning behind annual scans:  * **Early Detection:** Since lung involvement can progress silently, annual CT scans allow us to identify issues early when treatment is most effective.  * **Monitoring Progression:** By comparing yearly scans, we can track any changes in your lungs and adjust treatment as needed.  However, the decision for annual CT scans should be individualized. I would recommend discussing this with your rheumatologist. They can consider factors like your specific health status, severity of Sjögren's, and presence of any respiratory symptoms. They might determine chest X-rays or lung function tests might be sufficient in some cases, or that scans could be done less frequently than annually.  Let me know if you have any other questions about Sjögren's syndrome, lung involvement, or the role of CT scans in management. |
| 1. Positive antinuclear antibodies, does a higher titer indicate more severe disease? | A positive ANA test with a higher titer can be suggestive of a more serious underlying condition, but it's not a definitive indicator. Here's a breakdown:  * **Positive ANA:** This simply means your body is producing antibodies that target parts of the cell nucleus. This can happen due to autoimmune diseases, infections, or even certain medications.  * **Titer:** This refers to the dilution at which the antibodies are still detectable in the blood. A higher titer (like 1:640) generally indicates a stronger immune response compared to a lower titer (like 1:40).  In the context of autoimmune diseases, a higher ANA titer **might** suggest a more active or widespread disease process. However, there are important things to consider:  * **Not a guarantee:** Not everyone with a high ANA titer has a severe illness. Conversely, some people with severe autoimmune disease might have a low titer.  * **Other factors:** The specific type of ANA pattern and your overall clinical picture (symptoms, medical history) are crucial for diagnosis and assessing disease severity.  **My recommendation:** If you have a positive ANA test result, discuss it with the doctor who ordered the test. They can interpret the result in the context of your specific situation and determine any necessary follow-up steps.  Here are some additional resources you might find helpful:  * American College of Rheumatology on ANA: [American College of Rheumatology Antinuclear Antibodies]  * RheumInfo on ANA: [Anti Nuclear Antibody ANA Blood Test] |
| **Topic: Prevention and Treatment** | |
| 1. What are the high-risk factors for autoimmune diseases? | Great question! While the exact cause of autoimmune diseases remains under investigation, there are definitely some well-established risk factors. Here are a few key ones:  * **Genetics:** Having a family member with an autoimmune disease increases your risk.  * **Sex:** Women are more susceptible to autoimmune diseases overall.  * **Age:** While autoimmune diseases can strike at any age, some are more common during certain life stages.  * **Certain infections:** Some viral or bacterial infections can trigger autoimmune responses.  * **Environmental factors:** Exposure to toxins, pollutants, and even sunlight can play a role.  * **Diet:** Certain dietary patterns may be linked to autoimmunity.  It's important to remember that these factors act more like a puzzle with many pieces. Having one risk factor doesn't guarantee you'll develop an autoimmune disease. If you're concerned about your individual risk, talking to your doctor is always the best course of action. |
| 1. Should autoantibodies be tested during routine physical examinations? | That's an interesting question. Here's the thing about autoantibody testing in routine physicals:  * **Generally, no** - Routine physical exams typically focus on identifying risk factors for common diseases and catching potential problems early. Autoantibody presence often indicates an autoimmune disease, but many people have these antibodies without ever developing symptoms.  * **Targeted testing is better** - If you have concerning symptoms that suggest an autoimmune condition, then your doctor might order specific autoantibody tests to help with diagnosis. For example, an ANA (antinuclear antibody) test might be used to investigate lupus.  * **Unnecessary cost and anxiety** - Widespread autoantibody testing in healthy individuals can lead to unnecessary costs and potentially cause anxiety due to positive results that may not be clinically significant.  However, there are some exceptions:  * **Family history** - If you have a strong family history of autoimmune diseases, your doctor might discuss the possibility of targeted autoantibody testing.  Ultimately, the decision of whether to test for autoantibodies depends on your individual situation and medical history. If you have concerns, discuss it with your doctor. They can help you weigh the potential benefits and risks of testing.  Here are some additional points to consider:  * The specific autoantibody test being considered.  * The cost of the test and your insurance coverage.  * Your personal comfort level with potentially positive results.  I hope this helps! Let me know if you have any other questions about autoantibodies or any other lab tests we perform. |
| 1. Should antinuclear antibodies be tested during routine physical examinations? | No, antinuclear antibody (ANA) tests aren't typically included in routine physical exams for healthy individuals.  Here's why:  * **Specificity:** While a positive ANA test can indicate an autoimmune disease, many healthy people also have a positive ANA. This can lead to unnecessary worry and further testing.  * **Symptoms Matter:** ANA tests are more valuable when a patient is experiencing symptoms suggestive of an autoimmune condition, like fatigue, joint pain, or unexplained rashes. In these cases, the ANA result can help guide further evaluation for specific diseases like lupus or rheumatoid arthritis.  However, if you have a family history of autoimmune disease or are concerned about your own risk, talk to your doctor about whether an ANA test might be right for you. They can consider your individual situation and determine if the test would be beneficial. |
| 1. Do autoimmune diseases have a hereditary component? My mother has Sjögren's syndrome, how can I prevent myself from getting sick? | That's a very important question. Yes, autoimmune diseases can have a hereditary component. This means there's a genetic predisposition that can increase your risk. In Sjögren's syndrome specifically, certain genes linked to the immune system are associated with a higher risk.  However, having a family member with an autoimmune disease doesn't guarantee you'll develop one yourself. Other factors, like environmental triggers, also play a role.  Here's what we can do:  * **Genetic testing:** While not routine for Sjögren's syndrome, genetic tests can assess your risk for certain autoimmune diseases. This is a discussion you can have with your doctor to see if it's right for you.  * **Be aware of symptoms:** Sjögren's syndrome often presents with dry eyes and mouth. Early detection and treatment can help manage the condition.  * **Maintain a healthy lifestyle:** This includes a balanced diet, regular exercise, and getting enough sleep. These factors can contribute to overall immune health.  If you're concerned about your risk, talking to your doctor is the best course of action. They can assess your individual situation and advise on preventative measures.  Remember, the lab department can be a partner in your health journey. If you undergo any autoimmune workup, we'll be here to provide accurate and timely results. |
| 1. I have systemic lupus erythematosus, what should I pay attention to in daily life? | Dr. Patel here. While I can't give specific medical advice, I can share some general tips for managing lupus daily life that you can discuss with your rheumatologist (arthritis specialist). These are especially important during flare-ups:  * **Prioritize rest:** Fatigue is a common lupus symptom. Listen to your body and schedule breaks throughout the day.  * **Manage stress:** Stress can worsen lupus symptoms. Consider relaxation techniques like meditation or deep breathing.  * **Sun protection:** Sun sensitivity is a common lupus issue. Use sunscreen daily, wear protective clothing, and avoid midday sun.  * **Healthy diet:** Eating a balanced diet rich in fruits, vegetables, and whole grains can improve your overall health and well-being.  * **Exercise:** Regular, gentle exercise can help manage fatigue and joint pain. Talk to your doctor about what exercise program is right for you.  * **Medications:** Take your medications exactly as prescribed by your doctor. Don't hesitate to ask questions about side effects or potential interactions.  * **Doctor communication:** Maintain open communication with your rheumatologist. Report any changes in symptoms or anything that concerns you.  Remember, everyone experiences lupus differently. These are general guidelines, and what works for one person might not work for another. Your rheumatologist will be able to tailor a personalized plan to effectively manage your lupus and improve your quality of daily life. |
| 1. I have systemic lupus erythematosus, and my condition is stable under medication. What indicators should I regularly check? | Hi there, thanks for reaching out. Since you have SLE, regular monitoring is important to track disease activity and potential complications. While I can't give specific recommendations, here are some general indicators commonly checked for lupus patients:  * **Autoantibody tests:** These look for antibodies your immune system produces that attack healthy tissue, a hallmark of lupus. The most common is the antinuclear antibody (ANA) test, but others may be included depending on your specific situation.  * **Complete blood count (CBC):** This assesses red and white blood cell counts, which can be abnormal in lupus due to inflammation or immune system issues.  * **Urinalysis and urine protein-to-creatinine ratio (UPCR):** These check for protein in the urine, which can be a sign of kidney involvement, a common complication of lupus.  * **Inflammatory markers:** Tests like erythrocyte sedimentation rate (ESR) or C-reactive protein (CRP) can indicate general inflammation, which may flare with lupus activity.  However, the most appropriate tests and monitoring frequency will depend on your individual case, disease severity, medications, and your doctor's assessment. It's best to discuss these specifics with your rheumatologist, who can tailor a personalized plan to keep you stable. |
| 1. I have systemic lupus erythematosus, and my condition is stable under medication. How often should I go to the hospital for a check-up? | I can't give specific medical advice, but that's a great question for a rheumatologist, the specialist who manages lupus. However, based on general practices, for stable lupus patients with good medication control, follow-up visits are typically every 3-4 months [study on lupus check-up frequency].  These visits will likely include blood tests at the lab to monitor your condition. The specific tests ordered will depend on your doctor's assessment, but might include monitoring blood cell counts, checking for autoantibodies, and kidney function tests.  Of course, if you experience any new symptoms or a worsening of existing ones, don't hesitate to contact your doctor and schedule an appointment sooner. |
| 1. What are the common medications for autoimmune diseases? | I can't give specific medication advice, as that's best left to a doctor who knows your full medical history. However, I can share some general categories of medications commonly used for autoimmune diseases:  * **Immunosuppressants:** These medications dampen the immune system's response, aiming to reduce its attack on the body's own tissues. Examples include prednisone, azathioprine, and methotrexate.  * **Disease-modifying antirheumatic drugs (DMARDs):** These medications can slow the progression of certain autoimmune diseases, particularly rheumatoid arthritis.  * **Biologics:** These are newer targeted therapies that block specific molecules involved in the immune system's overreaction.  Remember, these are just broad categories, and there are many specific medications within each. It's crucial to consult with a doctor to determine the most appropriate treatment plan for your individual situation. They'll consider your specific autoimmune disease, disease severity, and other factors. |
| 1. What are the treatment principles for autoimmune diseases? | Dr. Patel here! While I can't speak to specific treatment plans (that's for your doctor), I can talk about the general principles used to manage autoimmune diseases. There are a few main goals:  * **Tamping down the immune response:** This is the core strategy, achieved through various medications that suppress the overactive immune system. Examples include corticosteroids and immunosuppressants.  * **Managing symptoms:** Many autoimmune diseases cause pain, inflammation, and other unpleasant issues. Medications like pain relievers and anti-inflammatory drugs can significantly improve quality of life.  * **Replacing lost function:** Some diseases, like type 1 diabetes, destroy the body's ability to produce essential substances. Treatment involves replacing those, like insulin in the case of diabetes.  It's important to remember these are just general principles. The specific course of treatment will vary depending on the particular autoimmune disease you have. |
| 1. I have systemic lupus erythematosus and am currently undergoing steroid therapy. What are the side effects of steroids? | I can't give specific medical advice, but I can share some general information about steroid side effects. Steroids are powerful medications used to treat lupus flares, but they can come with downsides.  Here are some common side effects to be aware of:  * **Short-term:** Weight gain, increased appetite, trouble sleeping, mood swings, acne.  * **Long-term:** High blood pressure, increased blood sugar (leading to diabetes), weakened bones (osteoporosis), cataracts, increased risk of infections.  It's important to remember that everyone reacts differently to medications, and the specific side effects you experience will depend on the dose, duration of treatment, and your individual health.  Since you have SLE and are on steroids, it would be best to talk to your doctor about the potential side effects and how to manage them. They can tailor advice to your specific situation and medical history. Would you like me to help you find some resources about lupus and steroid therapy? |
| 1. I have Sjögren's syndrome and am currently being treated with immunosuppressants (tacrolimus). What are the side effects? | Dr. Anya Patel here, Chief Physician of the Laboratory Department. Let's talk about tacrolimus and Sjögren's syndrome.  Tacrolimus is a medication that suppresses the immune system. It's commonly used to prevent rejection after organ transplants, but it can also be effective in treating autoimmune diseases like Sjögren's syndrome. By dampening the overactive immune response, tacrolimus can help alleviate symptoms like dry eyes and mouth.  That said, like any medication, there can be side effects. Here are some of the most common ones associated with tacrolimus:  * Kidney problems: Tacrolimus can affect kidney function. Your doctor will likely monitor your kidney function with regular blood tests.  * High blood pressure: Tacrolimus can increase blood pressure. It's important to have your blood pressure checked regularly.  * High blood sugar: Tacrolimus can increase blood sugar levels. If you have diabetes, your doctor will monitor your blood sugar closely.  * Headaches: Headaches are a common side effect of tacrolimus. Over-the-counter pain relievers may help.  * Diarrhea: Tacrolimus can cause diarrhea. If you experience this side effect, let your doctor know, they may recommend adjustments to your medication or recommend medications to manage diarrhea.  * Increased risk of infection: Because tacrolimus suppresses the immune system, it can increase your risk of developing infections. Be sure to practice good hygiene and tell your doctor right away if you develop any signs of infection, such as fever, chills, or cough.  It's important to note that this is not an exhaustive list of side effects. Everyone reacts differently to medications, so it's important to talk to your doctor about any side effects you experience. They can help you determine if the benefits of tacrolimus outweigh the risks.  Here are some additional things to keep in mind:  * Tacrolimus can interact with other medications, so be sure to tell your doctor about all the medications you are taking, including over-the-counter medications and supplements.  * Grapefruit juice can increase the levels of tacrolimus in the blood. Avoid grapefruit juice while taking tacrolimus.  * Sun exposure can increase your risk of sunburn while taking tacrolimus. Be sure to wear sunscreen with an SPF of 30 or higher and protective clothing when outdoors.  If you have any questions or concerns about tacrolimus, please don't hesitate to talk to your doctor. |
| 1. What are the different types of immunosuppressants? | There are several different types of immunosuppressants, which can be broadly categorized into four main classes:  * **Calcineurin Inhibitors:** These medications work by blocking a protein called calcineurin, which is involved in the activation of T cells, a type of white blood cell important in the immune system. Examples of calcineurin inhibitors include tacrolimus (Prograf) and cyclosporine (Sandimmune).  [Image of Tacrolimus medication]  [Image of Cyclosporine medication]  * **Antiproliferative Agents:** These medications prevent the growth and replication of T cells. Mycophenolate mofetil (CellCept) and azathioprine (Imuran) are two commonly used antiproliferative agents.  [Image of Mycophenolate mofetil medication]  [Image of Azathioprine medication]  * **mTOR Inhibitors:** Sirolimus (Rapamune) is the only medication in this class. It works by blocking a different pathway involved in T cell activation.  [Image of Sirolimus medication]  * **Steroids:** Prednisone is a corticosteroid medication that can suppress the immune system overall. It's often used in combination with other immunosuppressant medications.  [Image of Prednisone medication]  These medications are used to treat a variety of conditions, including:  * Autoimmune diseases, such as lupus, rheumatoid arthritis, and psoriasis  * After organ transplantation to prevent rejection of the new organ  * Certain cancers  It's important to note that immunosuppressants can have serious side effects, so they should only be taken under the supervision of a doctor. These side effects can include increased risk of infection, high blood pressure, kidney problems, and cancer. |
| 1. What dietary considerations should be taken during the use of immunosuppressants? | That's a great question, but dietary advice falls outside the realm of the laboratory department. However, I can share some general knowledge and point you in the right direction.  immunosuppressants can weaken your immune system, making you more susceptible to foodborne illness. To minimize that risk, certain dietary restrictions are important.  Here's what I recommend: Speak with your doctor or a registered dietitian specializing in immunosuppressant therapy. They can provide specific guidance based on your individual situation and medications. In the meantime, some general recommendations for immunocompromised patients include:  * Avoiding raw or undercooked meats, fish, and eggs.  * Consuming only pasteurized dairy products.  * Washing all fruits and vegetables thoroughly.  * Being cautious with buffets, salad bars, and pre-made deli foods.  Remember, this is just a starting point. A healthcare professional can give you tailored advice to keep you healthy while taking immunosuppressants. |
| 1. What immunosuppressants require monitoring of drug levels during use? | Most, if not all, immunosuppressant medications benefit from therapeutic drug monitoring (TDM) during use. This is because they often have a **narrow therapeutic window**.  Here's why monitoring is crucial:  * **Individual Variability:** People absorb and metabolize medications differently. TDM helps ensure the patient receives the correct dosage for their individual needs.  * **Narrow Window:** A small difference between too little medication (ineffective) and too much (toxic) makes close monitoring vital.  * **Risk of Rejection:** In organ transplant patients, under-dosing immunosuppressants can lead to organ rejection.  * **Infection Risk:** Over-immunosuppression increases the risk of infections.  While I can't give specific medical advice, common immunosuppressants requiring TDM include:  * Cyclosporine A  * Tacrolimus  * Mycophenolic Acid  * Sirolimus  * Everolimus  If you have a question about a specific medication, let me know the name and I can share whether TDM is typically recommended. |
| 1. I was recently diagnosed with systemic lupus erythematosus. How often should I get a check-up during treatment? | I can't give specific medical advice, but that's a great question for your rheumatologist (the specialist who treats lupus). However, I can share some general guidelines.  The frequency of check-ups for lupus depends on disease activity. Typically:  * For active disease: Follow-ups might be every 1-3 months after diagnosis or a flare-up.  * For low disease activity and stable treatment: Check-ups could be every 6-12 months.  Your rheumatologist will tailor a plan based on your individual situation. They'll likely order lab tests to monitor your progress and adjust medications as needed. These tests might include:  * Blood tests to assess blood cell counts, kidney and liver function, and autoantibody levels.  * Urine tests to check for protein or blood.  Feel free to ask your rheumatologist any questions you have about your check-up schedule or the lab tests involved. They'll be happy to explain everything in detail. |
| 1. What should a patient with gout consider in their daily diet? | While I can't give specific dietary advice, I can tell you that gout is linked to high uric acid levels in the blood. When it comes to diet, gout patients should focus on reducing purine intake. Purines are broken down into uric acid in the body.  If you have a diagnosed case of gout, it would be best to speak with a registered dietician or your doctor about creating a personalized gout management plan, which often includes dietary changes. They can advise you on specific foods to limit or avoid, as well as recommend beneficial options. |
| 1. I was recently diagnosed with systemic lupus erythematosus. Should my siblings visit a rheumatology and immunology department? | I can't give specific medical advice, Dr. (your doctor's name) will be the best person to advise your siblings on whether they should see a rheumatology and immunology department. However, I can share some general information.  Systemic lupus erythematosus (SLE) is an autoimmune disease, but it's not typically contagious. However, there is a small chance that siblings of someone with SLE have a slightly increased risk of developing an autoimmune disease themselves.  Here's what I can recommend:  * Discuss your concerns with Dr. (your doctor's name). They can assess your siblings' individual risk factors and advise them accordingly.  * If Dr. (your doctor's name) suggests a visit to a rheumatologist and immunologist, the department can perform tests to look for any early signs of autoimmune conditions.  In the meantime, you can share some reliable resources with your siblings, such as the Lupus Foundation of America [https://www.lupus.org/](https://www.lupus.org/). |
| 1. Which patients with positive autoantibody indicators have a higher chance of developing tumors? | That's an interesting question, and it touches on a developing area of research. Here's what we know currently:  * A positive autoantibody test indicates the presence of antibodies that target the body's own tissues.  * Certain autoimmune diseases are associated with an increased risk of developing specific cancers. For instance, Sjögren's syndrome carries a higher risk of lymphoma.  However, there isn't a universal link between all positive autoantibodies and a higher chance of developing any type of tumor. It depends on the specific autoantibody and the underlying condition.  If you're concerned about a positive autoantibody result and potential cancer risk, I recommend discussing it with your doctor. They can consider your specific autoantibody, medical history, and family history to determine if there's a need for further investigation.  In the meantime, our lab can certainly help with additional testing as ordered by your doctor.  Is there anything else I can help you with regarding autoantibodies or other lab tests? |
| **Topic: Prognosis** | |
| 1. I am a patient with rheumatoid arthritis. My anti-mutated citrullinated vimentin antibody test results were 870 U/mL a year ago and 562 U/mL a week ago. Does this indicate that my condition is improving? | Dr. Patel here from the Lab Department. That decrease in your anti-mutated citrullinated vimentin antibody levels is a promising sign! A lower level suggests potentially less disease activity.  However, interpreting lab results for rheumatoid arthritis (RA) involves a nuanced approach. While anti-MCV antibody tests are a helpful indicator, a definitive assessment of your condition requires a holistic review by your rheumatologist.  Here's why a doctor's review is crucial:  * **Your medical history:** They'll consider how long you've had RA, the severity of your symptoms, and previous treatment history.  * **Symptoms:** They will want to know if you've noticed any improvement in your pain, stiffness, or swelling in your joints.  * **Other tests:** They may consider X-rays or ultrasounds to assess joint damage and inflammation.  So, while the decrease in anti-MCV antibodies is encouraging, it's best to discuss these results with your rheumatologist to get a complete picture of your RA activity. They can advise you on whether this suggests improvement and tailor treatment accordingly. |
| 1. What is the prognosis for autoimmune diseases? | Dr. Patel here. Unfortunately, there isn't a one-size-fits-all answer for prognosis with autoimmune diseases. In general, there currently isn't a cure [autoimmune diseases causes ON Healthline [invalid URL removed]].  The good news is that many autoimmune diseases can be effectively managed. Through medications and other therapies, we can focus on controlling symptoms and preventing flare-ups. In some cases, the disease may even go into remission, meaning minimal to no symptoms for an extended period.  The outlook really depends on the specific disease. If you're concerned about a particular autoimmune condition, let me know the name and I can share more specifics about that disease.  Remember, early diagnosis is key! So if you have any concerns, talk to your doctor about possible autoimmune issues. They can order the appropriate lab tests to get a clearer picture. |
| 1. Which autoimmune diseases progress rapidly and have a poor prognosis? | Unfortunately, I can't give you a definitive answer to that because there isn't a single category of autoimmune diseases that all progress rapidly and have a poor prognosis. Many autoimmune diseases have a spectrum of severity, and prognosis depends on a variety of factors.  However, I can share some information that might be helpful:  * **Certain autoimmune diseases** are generally considered more serious than others. These include:  * **Acute disseminated encephalomyelitis (ADEM):** This is a rare autoimmune disease that attacks the brain and spinal cord. It can progress rapidly and cause significant damage.  * **Fulminant myocarditis:** This is a severe form of myocarditis, an inflammation of the heart muscle, caused by an autoimmune reaction. It can be life-threatening.  * **Systemic lupus erythematosus (SLE) with severe organ involvement:** When SLE affects vital organs like the kidneys or lungs, the prognosis can be worse.  * **The speed of progression** also depends on the individual and how early the disease is diagnosed and treated. Early diagnosis and intervention are crucial for improving the prognosis of many autoimmune diseases.  If you're concerned about a specific autoimmune disease, it's important to talk to your doctor. They can give you a more specific idea of the potential course of the disease based on your individual situation.  Here at the lab, we can run a variety of tests to help diagnose autoimmune diseases. These tests look for specific antibodies or markers of inflammation. If you have any questions about these tests, feel free to ask! |
